# Elucidating the Mechanism of Emodin in Treating Post‐Stroke Depression Through Network Pharmacology and Animal Experiments

**DOI:** 10.1111/cns.70581

**Published:** 2025-09-24

**Authors:** Xiaoju Liu, Jie Gao, Kai Yang, Weiming Zhu, Ming Su, Zichun Liu, Yaxin Yuan, Linya Cao, Tong Wu, Wei Liu

**Affiliations:** ^1^ Shandong University of Traditional Chinese Medicine Jinan China; ^2^ Shandong Polytechnic Colloge Jining China; ^3^ Affiliated Hospital of Shandong University of Traditional Chinese Medicine Jinan China; ^4^ Shandong Academy of Chinese Medicine Jinan China; ^5^ Shandong University of Traditional Chinese Medicine Second Affiliated Hospital Jinan China

**Keywords:** emodin, mBDNF, molecular docking, network pharmacology, post‐stroke depression, proBDNF

## Abstract

**Objective:**

Evaluate the mechanism of Emodin therapy for Post‐stroke depression (PSD) using network pharmacology and animal experiments.

**Methods:**

Firstly, the effectiveness of Em in treating PSD was confirmed by constructing a PSD rat model. Then, network pharmacology and molecular docking techniques were used to analyze the potential signaling pathways and targets of Em therapy for PSD. Further exploration and validation were conducted using the PSD rat model. Finally, the expressions of tissue plasminogen activator (tPA), matrix metallopeptidase‐9 (MMP9), furin, and proprotein convertases (PC) in the hippocampus and medial prefrontal cortex (mPFC) were further detected.

**Results:**

Em exhibited significant neuroprotective and antidepressant effects on PSD. Network pharmacology analysis revealed that Em may exert pharmacological effects on PSD through 47 core targets. These targets were involved in multiple signaling pathways. Molecular docking studies demonstrated that Em had a strong binding affinity for core targets. Animal experiments further indicated that Em could regulate the expression of precursor brain‐derived neurotrophic factor (proBDNF) and mature BDNF (mBDNF) in the hippocampus and mPFC, ameliorating apoptosis. In addition, Em could upregulate the expression of tPA gene and protein in the hippocampus and mPFC, as well as upregulate the expression of Furin gene and protein in the mPFC. This confirmed that the balance regulation of proBDNF/mBDNF depends on tPA and Furin.

**Conclusion:**

The therapeutic effects of Em on PSD are multi‐targets and multi‐pathways. Em may exert therapeutic effects on PSD by binding to the core target, BDNF. The mechanism may be to regulate the balance proBDNF/mBDNF via tPA and Furin, improving apoptosis.

## Graphical abstract

Em could alleviate the neurological impairment and depression behavior of PSD. Network pharmacology and molecular docking were used to explore the multiple mechanisms of Em therapy for PSD. The mechanism of Em in treating PSD may be achieved by regulating the proBDNF/mBDNF balance through tPA and Furin, improving cell apoptosis.

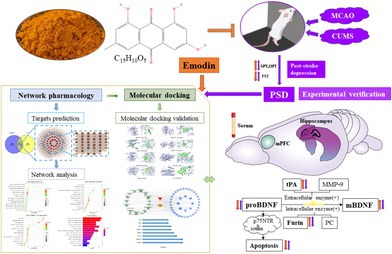

**Abbreviations**
5‐HT: Serotonin
BDNF: Brain‐Derived Neurotrophic Factor
cDNA: complementary DNA
CUMS: Chronic Unpredictable Mild Stress
Em: Emodin
FST: Forced Swimming Test
GO: Gene Ontology
KEGG: Kyoto Encyclopedia of Genes and Genomes
MAOIs: Monoamine Oxidase Inhibitors
mBDNF: mature Brain‐Derived Neurotrophic Factor
MCAO: Middle Cerebral Artery Occlusion
MMP9: Matrix Metalloproteinase 9
mPFC: medial Prefrontal Cortex
NIH: National Institutes of Health
OFT: Open Field Test
PC: Pyruvate Carboxylase
PMT: *Polygonum Multiflorum Thunb*.
PPI: Protein–Protein Interaction
proBDNF: precursor Brain‐Derived Neurotrophic Factor
PSD: Post‐Stroke Depression
qRT‐PCR: quantitative Reverse Transcription‐Polymerase Chain Reaction
SD: Sprague Dawley
SI: Social Isolation
SP: Sucrose Preference
SPT: Sucrose Preference Test
SSRIs: Selective Serotonin Reuptake Inhibitors
TCAs: Tricyclic Antidepressants
TCM: Traditional Chinese Medicine
tPA: tissue Plasminogen Activator
TTC: Triphenyltetrazole Chloride
TTD: Therapeutic Target Database
WB: Western Blot

## 1 Introduction

Post‐stroke depression (PSD) is a common neurological and psychiatric complication of stroke that affects approximately one‐third of stroke survivors. It not only leads to poor functional recovery and decreased daily living abilities in patients with stroke but is also the main cause of increased disability and mortality rates in patients with stroke [1]. Patients with early onset PSD (within 3 months after stroke) have a higher risk of persistent depression [2, 3]. Persistent depression can lower the quality of life, hinder recovery, and increase the risk of relapse [4, 5].

PSD pathogenesis is complex and may result from the joint action of neurobiological and sociopsychological mechanisms. The current hypotheses mainly include neurotrophic, monoamine transmitter, and inflammatory factor hypotheses. PSD is secondary to stroke and is associated with its pathological states. Clinical reports have demonstrated that low levels of brain‐derived neurotrophic factor (BDNF) may be present before the onset of stroke, which is prone to manifest as depression. Stroke triggers a hypoxic environment, and BDNF expression in the brain may be downregulated [6]. BDNF exists in two states in the brain: precursor (proBDNF) and mature (mBDNF). ProBDNF and mBDNF depend on each other, although their effects are opposite to each other. An imbalance in mBDNF/proBDNF homeostasis leads to neuronal death and decreased synaptic plasticity under pathological conditions. An altered mBDNF/proBDNF ratio has been observed in the plasma and cerebrospinal fluid of patients with depression and neurodegenerative diseases [7, 8]. Studies have found that the relative levels of proBDNF and mBDNF in the ischemic prefrontal cortex (PFC) and hippocampus of rats with PSD are imbalanced [9]. ProBDNF can promote the apoptosis of PFC neuronal cells and inhibit synapse regeneration [10], and exercise can increase the ratio of mBDNF/proBDNF in the ischemic hippocampus, enhance neuroplasticity, and alleviate PSD‐related depression‐like behaviors [11].

Studies suggest that abnormal digestion and transformation of proBDNF may reflect the negative biological effects of proBDNF and trigger neurotoxicity [12]. The key to BDNF balance regulation is mediated by the action of intracellular Furin and/or proprotein convertases (PC), plasmin activated by tissue plasminogen activator (tPA), and matrix metalloproteinase [13, 14, 15, 16, 17]. Whether BDNF cleavage enzymes regulate the balance between proBDNF and mBDNF by BDNF cleavage enzymes to participate in PSD is one of the mechanisms discussed in this study.

Currently, antidepressants are mainly used in clinical practice to treat PSD, alleviate depressive states, improve the quality of life, and prevent disease recurrence. Selective serotonin reuptake inhibitors, including fluoxetine, can improve depressive states; however, their clinical effects may take several weeks to be observed [18]. The efficacy of sertraline and Deanxit is non‐significant [19]. Monoamine oxidase inhibitors have significant side effects [20]. Tricyclic antidepressants also have a high risk of adverse reactions [21]. Inappropriate use of antidepressants may increase mortality rates [22]. To date, no specific drugs have been developed for treating PSD. In recent decades, traditional Chinese medicine (TCM), including natural products, extracts, and formulations, has been increasingly used to treat PSD [2, 23]. Research in ethnic pharmacology has reported that various natural products, such as echinacoside [24], morroniside [25], catapol [26], resveratrol [27], paeoniflorin [28], and aloe‐emodin [23] have potential benefits in treating PSD. Although progress has been made in developing drugs targeting PSD, there is an urgent need for safer and more effective anti‐PSD drugs.

*Polygonum multiflorum Thunb*. (*Pleuropterus multiflorus (Thunb.) Nakai*, PMT), a TCM with a long history, is widely used in the clinical treatment of various chronic diseases and has been given the reputation of “rejuvenating and prolonging life” in many places [29]. For its clinical application, raw and prepared PMT are distinguished, and both are listed in the 2020 Chinese Pharmacopeia. According to reports, raw PMT detoxifies, eliminates carbuncles, and relaxes the intestines, whereas prepared PMT tonifies the liver and kidney, aids blood essence, strengthens tendons and bones, and removes and reduces blood stasis. PMT is often used in preparing compounds such as Shouwu pills, Yangxue Anshen tablets, and Yangxue Shengfa capsules [29]. These preparations and health products containing PMT can be sold in both Chinese and foreign markets. Modern pharmacological studies have demonstrated that PMT has a wide range of clinical pharmacological effects [30], including anti‐inflammatory, antioxidant, and brain injury protection effects in rats [31], reduction of infarct volume induced by cerebral ischemia in gerbils [32], and improvement of functional outcomes after focal cerebral ischemia in mice [33]. PMT contains many components, including anthraquinones, stilbene glycosides, flavonoids, phospholipids, and phenolic compounds. Among them, Emodin (Em) an anthraquinone compound, is mainly responsible for the biological activity of PMT. Em, an indicator component of the medicinal value of PMT in the Pharmacopeia of the People's Republic of China (2020), exhibits a wide range of pharmacological activities, including nerve and heart protection [34, 35], prevention of atherosclerosis [36], anti‐cancer [37, 38], anti‐inflammatory, and antioxidant [39, 40] activities. Em can inhibit depressive like behavior, and upregulate the levels of glucocorticoid receptors and BDNF in the hippocampus of mice [41]. Em prevents depression in rats by targeting miR‐139‐5p/5‐lipoxygenase [42].

In addition, Em alleviates ischemic stroke by inhibiting aquaporin 4‐mediated swelling and neuroinflammation in rats [39]. Em exerts neuroprotective effects against ischemia/reperfusion injury by activating the ERK‐1/2 signaling pathway in rats [43]. Studies have suggested that Em relieves depression, improves neurological deficits in ischemic stroke, and plays a neuroprotective role in the brain. However, whether Em plays a significant role in treating PSD and its specific antidepressant and neuroprotective mechanisms remains unknown. Consequently, this study used a comprehensive pharmacological method combining network pharmacology and molecular docking, along with animal experimental verification, to explore the protective effect of Em against PSD and its underlying mechanisms.

**FIGURE 1:**
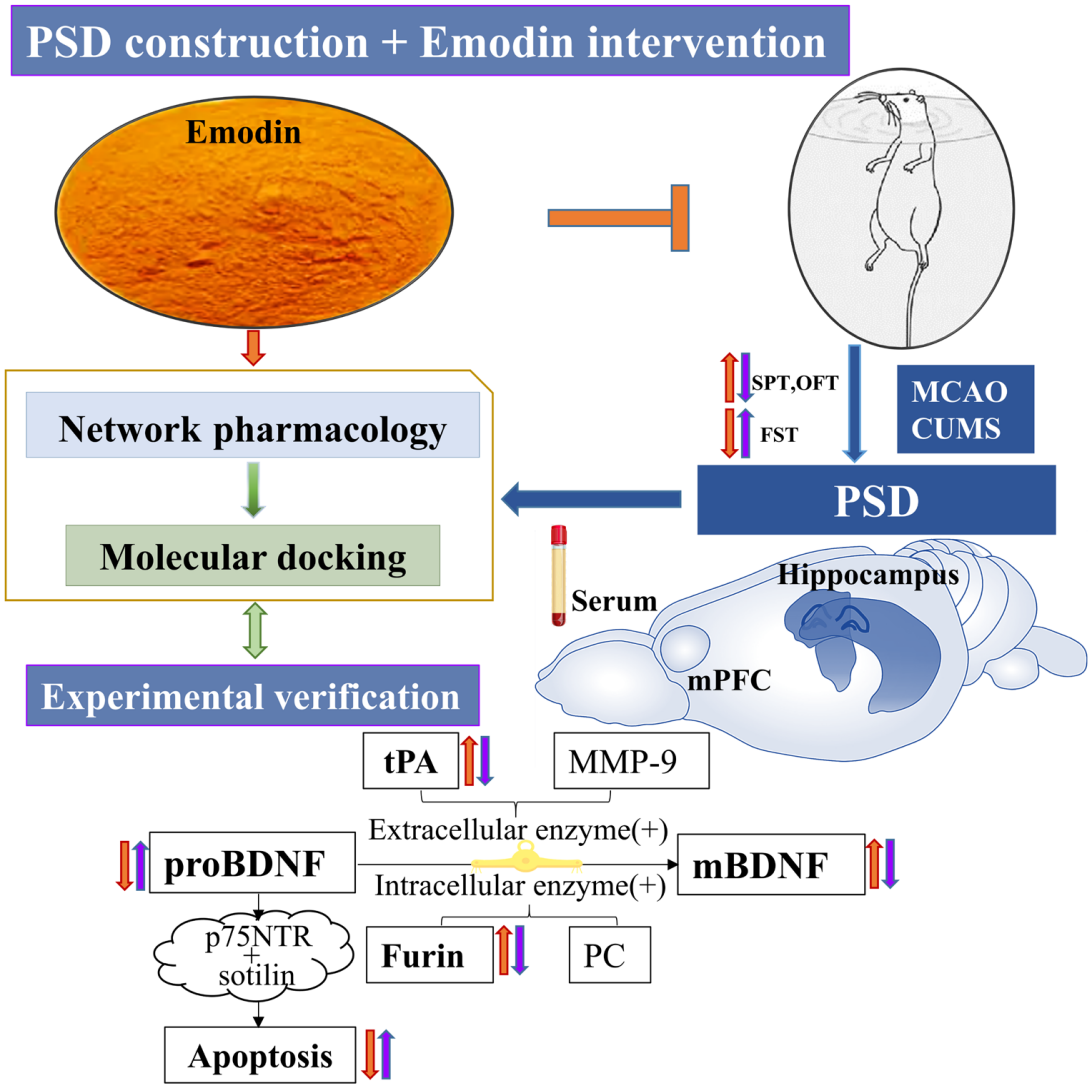
The flow chart of the proBDNF/mBDNF balance regulation mechanism of Em treatment for PSD using comprehensive network‐based pharmacology study. CUMS, chronic unpredictable mild stress; FST, forced swimming test; mBDNF, brain derived neurotrophic factor mature; MCAO, middle cerebral artery occlusion; MMP9, matrix metallprotenaine 9; mPFC, medial prefrontal cortex; OFT, open field test; proBDNF, brain derived neurotrophic factor precursor; PSD, post stroke depression; SPT, sucrose preference test; tPA, tissue plasminogen activator.

This study provides new insights and scientific evidence advocating Em as a potential therapeutic candidate for PSD. The design process of this study is illustrated in Figure 1. A PSD rat model was established using middle cerebral artery occlusion (MCAO) + social isolation (SI) + chronic unpredictable mild stress (CUMS), and it was found that EM could alleviate the neurological impairment and depression behavior of PSD. Network pharmacology and molecular docking were used to explore the multiple mechanisms of Em therapy for PSD, and the key signaling pathways and targets were predicted and analyzed. Finally, the effects and mechanisms of Em on PSD were confirmed using animal experiments. This study suggests that the mechanism of Em in treating PSD may be achieved by regulating the proBDNF/mBDNF balance through tPA and Furin, improving cell apoptosis, and exerting therapeutic effects.

## 2 Materials and Method

### 2.1 Chemicals and Reagents

Fluoxetine dispersible tablets (Cat# H20050463) were obtained from Lilly Suzhou Pharmaceutical Co. Ltd. (Suzhou, China). Em (purity above 95%, Cat# S30748) and carboxymethyl cellulose sodium (Cat# 14016) were sourced from Shanghai Yuanye Biotechnology Co. Ltd. (Shanghai, China). 2,3,5‐triphenyltetrazole chloride (TTC, Cat# T8877) was obtained from Sigma (USA). The TUNEL staining kit (Cat# E‐CK‐A321) was sourced from Wuhan Elabscience Biotechnology Co. Ltd. (Wuhan, China). TRIzol reagent (Cat# R711), complementary DNA (cDNA) synthesis kit (Hiscript) III RT SuperMix (Cat# R323), and ChamQ SYBR qPCR Master Mix (Cat# Q311) were procured from Vazyme Biotech Co. Ltd. (Nanjing, China). Primary antibodies against mBDNF (Cat# ab108319, 1:1000), matrix metallopeptidase‐9 (MMP‐9, Cat# ab76003, 1:500), and tPA (Cat# ab157469, 1:1000) were obtained from Abcam (Toronto, Ontario, Canada). The primary antibody against proBDNF (Cat# sc‐65,514, 1:500) was sourced from Santa Cruz Biotechnology (California, USA). Primary antibodies against Furin (Cat# 70393, 1:1000) and PC2 (Cat# 14013, 1:1000) were obtained from Cell Signaling Technology (Danvers, MA, USA). β‐actin antibody (Cat# R1207‐1, 1:10,000), Goal anti‐Rabbit antibody (Cat# HA1001, 1:50,000) were procured from Hua'an Biotech (Hangzhou, China), and Goal anti‐Mouse antibody (Cat# RGAM001, 1:3000) was obtained from Proteintech (Wuhan, China). Anhydrous ethanol (Cat# 10009259) was acquired from China National Pharmaceutical Chemical Reagent Co. Ltd. (Beijing, China). All reagents and compounds were standardized and obtained from the market.

### 2.2 Experimental Animal

Seventy‐two male Sprague Dawley (SD) rats aged 8 weeks (SPF, 280 ± 20 g) were sourced from Beijing Weitonglihua Experimental Animal Technology Co. Ltd. The rats were maintained on a 12‐h light/dark cycle at 21°C ± 1°C. All animal experimental procedures complied with the “Guidelines for the Care and Use of Laboratory Animals” issued by the National Institutes of Health (NIH) in the United States and were approved by the Experimental Animal Ethics Committee of the Shandong University of TCM (Approval Number: SDUTCM20230529002). Prior to the beginning of the experiment, the rats were allowed to feed ad libitum and acclimatized to the environment for 7 days.

### 2.3 Network Pharmacology

Em‐related targets were obtained from Swiss Target Predictions, PharmMapper, and the Comparative Toxicogenomics Database. We integrated and removed duplicate Em genes obtained from the above three databases and determined the remaining genes as the final target genes. PSD‐related targets were obtained from GeneCards, DrugBank, DisGeNET, and the Therapeutic Target Database. Common targets of Em and PSD were identified using a Venn diagram. Protein–protein interaction analysis of the targets was performed using the STRING database. The Metascape database was used for gene ontology (GO) functional enrichment and Kyoto Encyclopedia of Genes and Genomes (KEGG) pathway analysis. These analyses identified potential pathways for using Em to treat PSD. Subsequently, an Em‐PSD Targets Pathways network was constructed using Cytoscape software (version 3.9.0), and key therapeutic targets were identified.

### 2.4 Molecular Docking Validation

AutoDockTools software (version 1.5.7) was used for molecular docking to study the interactions between Em and key targets. The docking results were visualized using PyMOL software (version 2.3.4). The 3D structures of the core components were downloaded from the PubChem database. The entry numbers of the top five targets, ranked by their degree values, were searched in the UniProt database and input into the PDB database. Protein crystal structures with the source “ *Homo sapiens* ,” the detection method of “X‐ray diffraction,” and uncertainty ≤ 2 were downloaded. AutoDockTools software (version 1.5.7) was used for the hydrogenation and charge removal of target proteins and small molecules, which were saved in the pdbqt format. AutoDock software (version 4) was used to dock the ligands and receptors, and the target name, PDB ID, structure, binding energy, and other information are summarized in Table 3. Finally, PyMOL software (version 2.3.4) was used to visualize the results. The interaction in molecular docking is the core of molecular docking, and its essence is the binding force between the ligand and receptor through various non‐covalent bonds and spatial complementation. Generally, the binding energy is selected to reflect the role of a project in quantifying the molecular docking.

The binding energy is interpreted as follows:

- Binding energy > 0: Unfavorable binding;
- 0 to −5 kcal/mol: Weak binding, low affinity, and probably no biological activity;
−5 ~ −8 kcal/mol: Moderate binding, observable binding, but may need optimization;
−8 ~ −12 kcal/mol: Strong binding, typical drug‐target interaction.

Binding energy < −12 kcal/mol: Extremely strong binding; may be overfitted or contain errors.

Basic information on the databases used for screening data on Em treatment for PSD is presented in Table 1.

**TABLE 1:** Basic information of the database used for screening data on emodin treatment for PSD.

Name	URL
PubChem	https://pubchem.ncbi.nlm.nIh.gov/
SwissTarget Prediction	http://www.swisstargetprediction.ch/
Uniprot	http://www.uniprot.org/
STITCH	http://stitch.embl.de/
GeneCards	https://www.genecards.org/
OMIM	http://omim.org/
DisGeNET	https://www.disgenet.org/
Venny 2.1	https://bioinfogp.cnb.csic.es/tools/venny/index.html
STRING	https://string‐db.org/
SEA	http://sea.edbc.org
CTD	https://ctdbase.org/
pharmmapper	http://www.lilab‐ecust.cn/pharmmapper/
Bioinformatics	http://www.bioinformatics.com.cn/
RCSB PDB	http://www.rcsb.org/

### 2.5 Animal Grouping and Management

The animals were randomly divided into four groups using a random number table method: Sham, PSD, PSD + Fl, and PSD + Em.

1. *Sham group*: 9 rats were included, 3 rats/cage, standard bedding. Rats were fed normally and subjected to grasp stimulation for 1 week. Following the MCAO model replication process, grasping, fixation, anesthesia, vascular separation, and suturing were performed without inserting the thread.
2. *PSD group*: 9 rats were included. After 3 days of MCAO + SI + CUMS, 0.03% carboxymethyl cellulose sodium (1 mL/100 g) was given by intragastric administration.
3. *PSD + Fl group*: 9 rats were included. After 3 days of MCAO + SI + CUMS, Fl solution was given by intragastric administration (as a positive control drug, fluoxetine dissolved in 0.03% carboxymethyl cellulose sodium, 2.7 mg/kg/day, 1 mL/100 g) [44, 45].
4. *PSD + Em group*: 9 rats were included. After 3 days of MCAO + SI + CUMS, Em solution (Em dissolved in 0.03% carboxymethyl cellulose sodium, 80 mg/kg/day, 1 mL/100 g) was given by intragastric administration [41, 46, 47].

Starting from the first day of postoperative CUMS, the medication was given by intragastric administration daily for 21 consecutive days.

### 2.6 PSD Model and Experimental Procedures

**FIGURE 2:**
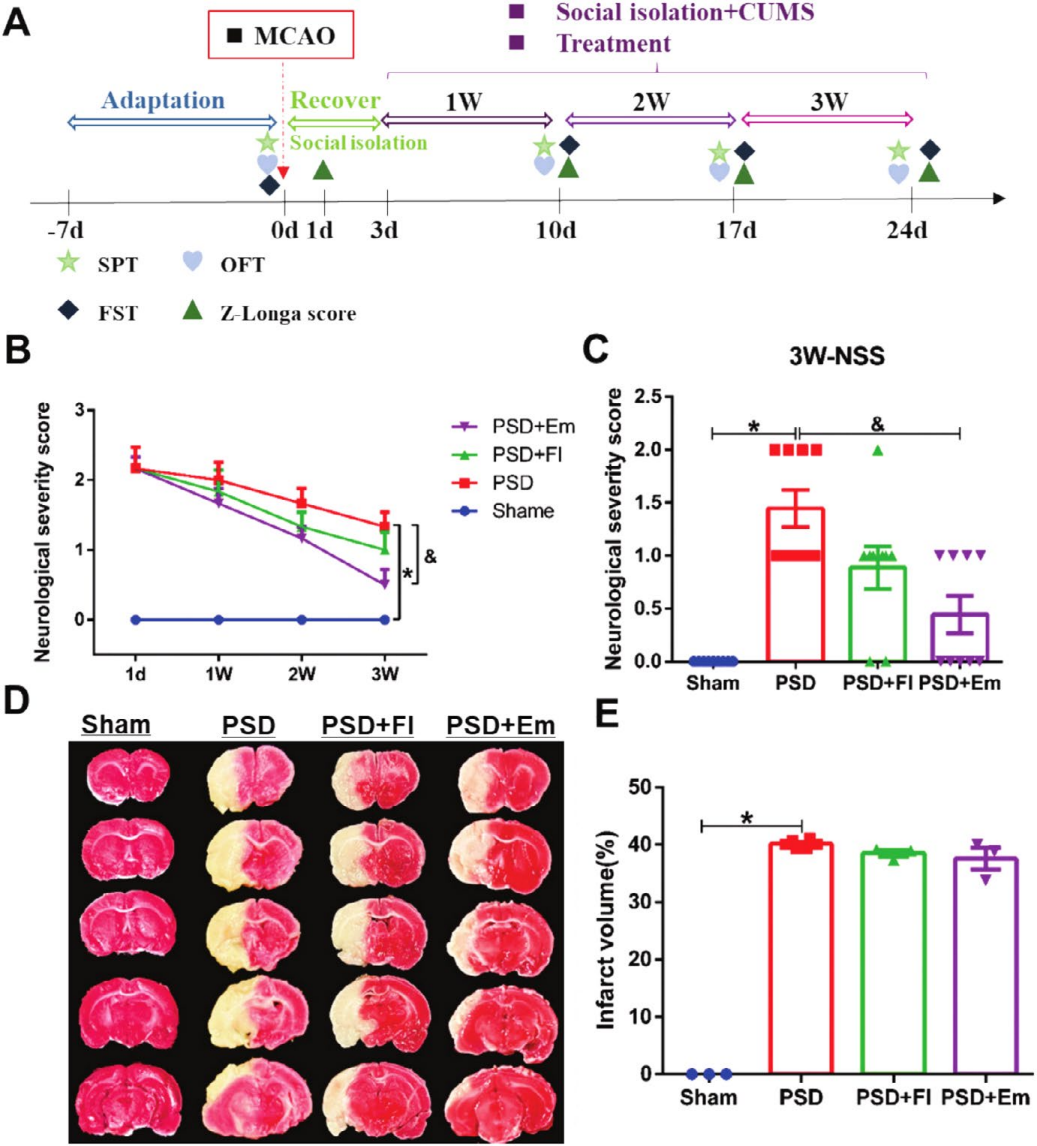
Animal experiment design process and neuroprotective effects of emodin on PSD rats. (A) Experimental design process for animal studies. (B) The effect of emodin on neurological deficit scores in PSD rats over time. *n* = 9. (C) The impact of emodin on neurological deficit scores in PSD rats at the end of the third week. *n* = 9. (D) Representative images of TTC staining. *n* = 3. (E) The influence of emodin on the infarct volume in PSD rats. *n* = 3. * comparisons between the PSD group and the Sham group, # comparisons between the PSD + Fl group and the PSD group, & comparisons between the PSD + Em group and the PSD group. *p* < 0.05.

The MCAO + SI + CUMS method is currently the most widely used classical modeling method for cerebral ischemia combined with depression [28, 29, 48]. The animal experimental design is illustrated in Figure 2A. The Z‐Longa score was used to evaluate neurological deficits in rats, and the depression status of rats was assessed using the sucrose preference test (SPT), open field test (OFT), and forced swimming test (FST). Rats without neurological deficits or depression‐like behavior were excluded (Table 2).

**TABLE 2:** Primer information in qRT‐PCR.

Primer name	Primer sequence (5′–3′)
MMP9‐F	ACTTCGACGCTGACAAGAAGTG
MMP9‐R	AATGATCTAAGCCCAGCGCATG
PC‐F	AAAGATTGCACCCTACGTTGCC
PC‐R	ACTCATACAAGAAGCGCATGGC
Furin‐F	TGACAACAGGCACGGCACTC
Furin‐R	TACACCTACACCACAGACACCATTG
BDNF‐F	TGGAACTCGCAATGCCGAACTAC
BDNF‐R	TCCTTATGAACCGCCAGCCAATTC
tPA‐F	GTTGTGCGTCCTGCTGCTTTG
tPA‐R	GCTCCTCTTCTGAACCTCCTGTG

### 2.7 MCAO Rat

A rat model of MCAO was established using the suture method described by Li et al. [2]. Briefly, isoflurane was used to anesthetize the rats, and an incision was made along the midline of the neck to expose the left common carotid artery. The proximal end of the common carotid artery was then ligated. A suture (Cat# 2636‐5‐A4, Xinong, Beijing, China) was gently inserted from the left common carotid artery lumen into the internal carotid artery and fixed. The body temperature of the rats was maintained at 37°C ± 0.3°C. The following day, a neurological deficit assessment was performed.

### 2.8 Neurological Function Assessment

The Z‐Longa score was used for neurological function assessment [49]. 0 = no neurological deficit; when a rat was lifted by its tail, its right front paw was bent, suggesting a mild neurological deficit; 2 = spontaneously turned right while walking, suggesting a moderate neurological deficit; 3 = tilted to the right, suggesting a severe neurological deficit; 4 = no spontaneous movement and low level of consciousness, suggesting an endangered status. Rats with neurological scores ≥ 1 but < 4 were included in the experiment. There were 5 rats with score 0, 12 rats with score 1, 21 rats with score 2, 16 rats with score 3, and 9 rats with score 4. Five rats with a score of 0 were excluded, and 21 rats eventually died after modeling, and 10 rats died during cums stimulation, and 27 rats were successfully modeled and survived. Initially, 9 rats were included in each group, and all animals successfully completed modeling and neurological function assessment. In the behavioral testing (SPT/OFT/FST), all 9 animals were included in the analysis.

### 2.9 CUMS

The depression rat model was replicated using SI and CUMS [50, 51], and the CUMS program was executed as previously described [29] with slight modifications. SI is to place experimental animals (such as rats or mice) in cages alone (i.e., single cage feeding) and completely isolate them from their peers. Social isolation is a commonly used means of stress, which is used to study the behavioral and neurobiological changes related to mental diseases such as loneliness, depression, anxiety, etc. In the PSD model, social isolation may be used as an additional stress factor to simulate the depressive symptoms aggravated by the lack of mobility and social contact after human stroke. Starting from the third day after MCAO combined with isolation, a 21‐day CUMS was performed in rats. Several different stressors were randomly assigned, including tail clipping (the rats were clamped 1 cm away from the tail root and relaxed for 10 s every minute for a total of 3 min), swimming in cold water (4°C) for 5 min, heat stress (45°C) for 5 min, restraint for 3 h, intermittent noise (90 dB) for 12 h, night lighting for 12 h, strobe light exposure for 12 h, dirty cage for 24 h, tilted cage for 24 h (45°C), water shortage for 21 h, followed by a SPT, food deprivation for 24 h, and forced swimming experiment. The rats were randomly exposed to different stressors (1–2 types/day, short‐ + long‐term stimulation, 21 days in total), making it difficult for animals to predict the stimuli and preventing the continuous use of the same stressor. Except for the sham group, all other rats were exposed to the same type, number, order, time, and degree of stimulation every day.

### 2.10 SPT

SPT was conducted as previously described [28], and the animals were given adaptive feeding to enhance their adaptation to drinking sucrose water. Initially, a bottle containing sucrose water and another containing purified water were provided before the testing began. After 24 h of domestication, the rats were limited to drinking water for 21 h during the test period. Each rat was provided with a bottle of 1% sucrose solution and a bottle of purified water. After 3 h, the bottles were weighed to calculate sucrose preference (SP).

SP = sucrose water consumption/total liquid consumption × 100%.

### 2.11 OFT

The OFT was conducted as described previously [28]. The open field box (50 × 50 × 50 cm) was divided into 16 compartments, with the central area containing 4 compartments. The movement trajectory of the rats within 5 min was recorded, and the following behavioral changes were analyzed: total and central distances, central retention time, along with the number of upright movements. Before formal testing, the rats were given 30 min to adapt to the environment, and the testing room was cleaned with 75% alcohol to eliminate odors that might affect further testing.

### 2.12 FST

FST was conducted as described previously [23] with slight adjustments. Rats were placed in a transparent cylinder (40 cm high and 20 cm diameter) with a water temperature of 23°C and a water depth of 30 cm, and the cylinder was placed in a forced swimming tail box to block external interference. The Smart software (version 3.0) animal behavior analysis system was used to capture and record the suspension time of rats in the last 5 min of the 6 min experiment. The water was changed every 2–3 rats to ensure cleanliness and reduce the interference from feces, odors, and other factors on the experimental results. After swimming, the rats were dried and returned to their original cages.

### 2.13 Sampling Method

After the behavioral test, all rats were euthanized, 5 mL of blood was collected from the abdominal aorta, and the brain was quickly decapitated and rinsed with ice‐ cold PBS. The arterial blood was centrifuged at 4000 rpm for 10 min at 4°C, and the upper serum was collected, stored at −80°C for a long time and −20°C for a short time; the brain tissue used for TTC staining was placed at −20°C for temporary storage, and then TTC staining was performed. Brain tissue for TUNEL staining was fixed in 4% paraformaldehyde at 4°C for 24 h and then dehydrated in 10%, 20%, and 30% sucrose solution at 4°C. The brain tissues used for QRT‐PCR and WB detection were quickly removed from the mPFC and hippocampal brain regions on ice and stored at −80°C for a long time and −20°C for a short time.

### 2.14 Enzyme Immunoassay of Serum proBDNF and mBDNF

After sampling, the levels of proBDNF and mBDNF in the serum were measured according to the guidelines provided by the manufacturer of each enzyme‐linked immunosorbent assay kit.

1. Preprocessing: Place the sample on ice and slowly melt it; 4°C. Centrifuge at 3000 rpm for 5 min to remove sediment. Incubate the reagent at room temperature for 1 h, prepare a washing solution (concentrated washing solution: distilled water = 1:20) and a substrate mixture (substrate A: substrate B = 1:1).
2. Operating procedure:
  - Take flat noodles, blank hole: no liquid, other holes: 50 μL; standard well: six different concentration standard samples, 0‐value well: sample dilution, sample well: test sample (six samples per group); two replicates.
  - Blank well: no liquid added, 0‐value well, standard well, and sample well: horseradish peroxidase (HRP) labeled detection antibody, 100 μL.
  - Cover with a sealing film, incubate at 37°C for 1 h, and avoid light.
  - Uncover the sealing film, discard the liquid, pat dry, fill with detergent, let it stand for 20 s, shake off the detergent, pat dry, and repeat this process five times.
  - Add 100 μL of substrate mixture. Cover with a sealing film, incubate at 37°C for 15 min, and avoid light.
  - All wells: Termination solution, 50 μL. ELISA reader (Multiskan Sky, Thermo, USA): 450 nm wavelength, read OD value.
  - Calculation: Fit the standard curve (four parameter logical fitting) with 6 standard concentrations (X) and OD values (Y) to calculate the concentrations of proBDNF and mBDNF in the sample.

### 2.15 TTC Staining

After the behavioral experiment, the rats were deeply anesthetized, the brains were removed on ice, placed in the rat brain mold, and frozen at −20°C for 30 min until completely frozen. Seven blades were inserted into the brain mold at the same time, and they were cut into 2 mm coronal sections for 5. Immediately put into 2% TTC and avoid light for 30 min at 37°C water bath. After staining, the brain sections were put into 4% paraformaldehyde, fixed at 4°C for 24 h, and then photographed for storage. The cerebral infarction volume was calculated. The infarct volume was calculated using the formula described by Li et al. [52].

### 2.16 TUNEL Staining

After the experiment, the ischemic hippocampus and medial prefrontal cortex (mPFC) of rats were collected, and the apoptosis of neuronal cells in the ischemic hippocampus and mPFC was detected according to the guidelines provided by the manufacturer's instructions for the TUNEL staining kit.

1. Fixation and permeabilization: take out the frozen section and equilibrate it to room temperature; immerse in the fixative for 30 min at room temperature; rinse twice with PBS for 5 min; add 1 × proteinase K working solution (about 100 μL/sample), 37°C, 16 min; rinse with PBS for 3 times × 5 min.
2. Preparation of marking working fluid (fully mixed, ready to use and ready to prepare): TdT Equilibration Buffer:Labeling solution:TdT Enzyme = 7:2:1.
3. Marking steps: add 100 μL TDT equilibration buffer dropwise and wet box at 37°C for 20 min; after blotting, add the labeled working solution (50 μL/sample) dropwise, and keep the wet box away from light at 37°C for 2 h; rinse with PBS for 3 times × 5 min; blot dry, add DAPI working solution dropwise for 5 min (keep away from light at room temperature); rinse with PBS for 4 times × 5 min; blot, sealing agent sealing.
4. Detection: fluorescence microscope observation.
5. TUNEL^+^ cells were quantified in five random fields (40×) per sample using ImageJ, with the apoptosis rate calculated as (TUNEL^+^/DAPI^+^) × 100. Data were analyzed by one‐way ANOVA with Tukey's test (GraphPad Prism 10.1) and presented as mean ± SEM (*n* = 3/group).

### 2.17 RNA Extraction and Quantitative Reverse Transcription‐Polymerase Chain Reaction (qRT‐PCR)

The expression of target genes BDNF and splicing enzymes tPA, Furin, MMP‐9, and PC2 in the ischemic hippocampus and mPFC was detected using qRT‐PCR. Total RNA was extracted from the hippocampus and mPFC of three rats in each group using the TRIzol reagent, and its integrity was confirmed by RIN > 7.0 (Agilent 2100). RNA concentration was measured using an ultra‐thin nucleic acid analyzer (Allsheng Nano‐100; China). For qPCR, 1000 ng RNA was reverse transcribed (Hiscript) III RT SuperMix, Cat# R323. Each reaction contained 50 ng cDNA, ChamQ SYBR qPCR Master mix (Cat # Q311, Vazyme), and gene‐specific primers (efficiency: 95%–105%, *R* ^2^ > 0.99). The samples were processed three times using a real‐time PCR system (Thermo PikoReal 96; USA) and normalized to β‐actin. The comparison between NTC and NRT showed no pollution. The mRNA levels of the target gene were expressed as 2^−ΔΔ*Ct*^ (ΔΔ*C*_*t*_ = *ct*_target_ − *ct*_reference_). All qRT‐PCR primers were custom‐synthesized by Shenggong Bioengineering Co. Ltd. (Shanghai, China) and validated for specificity and efficiency in this study. The complete sequences of all primers are listed in Table 2.

### 2.18 Western Blot (WB)

WB was used to detect protein expressions of proBDNF, mBDNF, and the cleavage enzymes tPA, Furin, MMP‐9, and PC2 in the hippocampus and mPFC of rats. mPFC tissue was dissected and separated on ice using Paxinos & Watson coordinates (Bregma +2.7 to +3.2 mm), rapidly frozen, and continuously lysed in RIPA buffer for 30 min, and homogenized using ultrasound. The lysate was centrifuged at 12,000 rpm for 10 min to obtain the supernatant and tissue proteins. Protein concentration was determined using the BCA method. Then, a 5× loading buffer was added to the sample, and the sample was boiled. The target proteins (25 μ g/lane) were separated on 10% sodium dodecyl sulfate‐polyacrylamide gel electrophoresis (120 V, 80 min), transferred to a PVDF membrane (400 mA, 90 min), and sealed with 5% skimmed milk for 1 h. The primary antibodies proBDNF (1:500), mBDNF (1:1000), tPA (1:1000), Furin (1:1000), PC2 (1:1000), and MMP9 (1:500) were incubated overnight at 4°C, and the HRP coupled secondary antibodies (1:50,000 or 1:3000) were incubated for 1 h, and ultrasensitive ECL reagent and gel imaging system (Fusion Solo S, Vilber Bio Imaging) were used to detect the signal. ImageJ software (version 2.0) was used to measure the quantitative band strength, with data presented as mean ± SEM (*n* = 3 biological replicates). The complete uncut imprint image is provided in File S1. Crop is only used to delete unused lanes and does not splice non‐contiguous lanes. The sample size used for WB analysis is relatively small, so the Mann Whitney *U* test was added during data analysis to evaluate WB gray value and verify the reliability of the results. The results are detailed in Tables S1 and S2.

### 2.19 Statistical Analysis

All data were analyzed using GraphPad Prism software (version 10.1, San Diego, CA, USA), and normality tests were performed on all quantitative data using the Shapiro Wilk test (sample size *n* < 50) or the Kolmogorov Smirnov test (*n* ≥ 50). The data conformed to a normal distribution and satisfied homogeneity of variance. One‐way analysis of variance (ANOVA) was used for intergroup comparisons, and Tukey's test was used for further pairwise comparisons. Repeated measurement data, such as the SP coefficient, were analyzed using two‐way ANOVA, and quantitative data were expressed as mean ± standard error of the mean, with a statistical significance level set at *p* < 0.05.

## 3 Results

### 3.1 Neuroprotection of Em on Rats With PSD

As depicted in Figure 2B,C, the neurological deficit scores in rats with PSD were significantly elevated (*p* < 0.001), whereas Em significantly reduced these scores (*p* < 0.05), and Fl did not produce a similar effect (*p* > 0.05). The infarct volume in the brains of rats with PSD was markedly increased (*p* < 0.001), but none of the drug interventions ameliorated this condition (*p* > 0.05) (Figure 2D,E). This suggests that the neuroprotective effects of Fl on PSD may be limited. Conversely, Em appears to have neuroprotective potential and ameliorates neurological deficits without altering the infarct volume. This indicates that Em may exert its neuroprotective effects on PSD via mechanisms other than altering the infarct volume.

### 3.2 Mitigating Effect of Em on Depression‐Like Behavior in Rats With PSD

**FIGURE 3:**
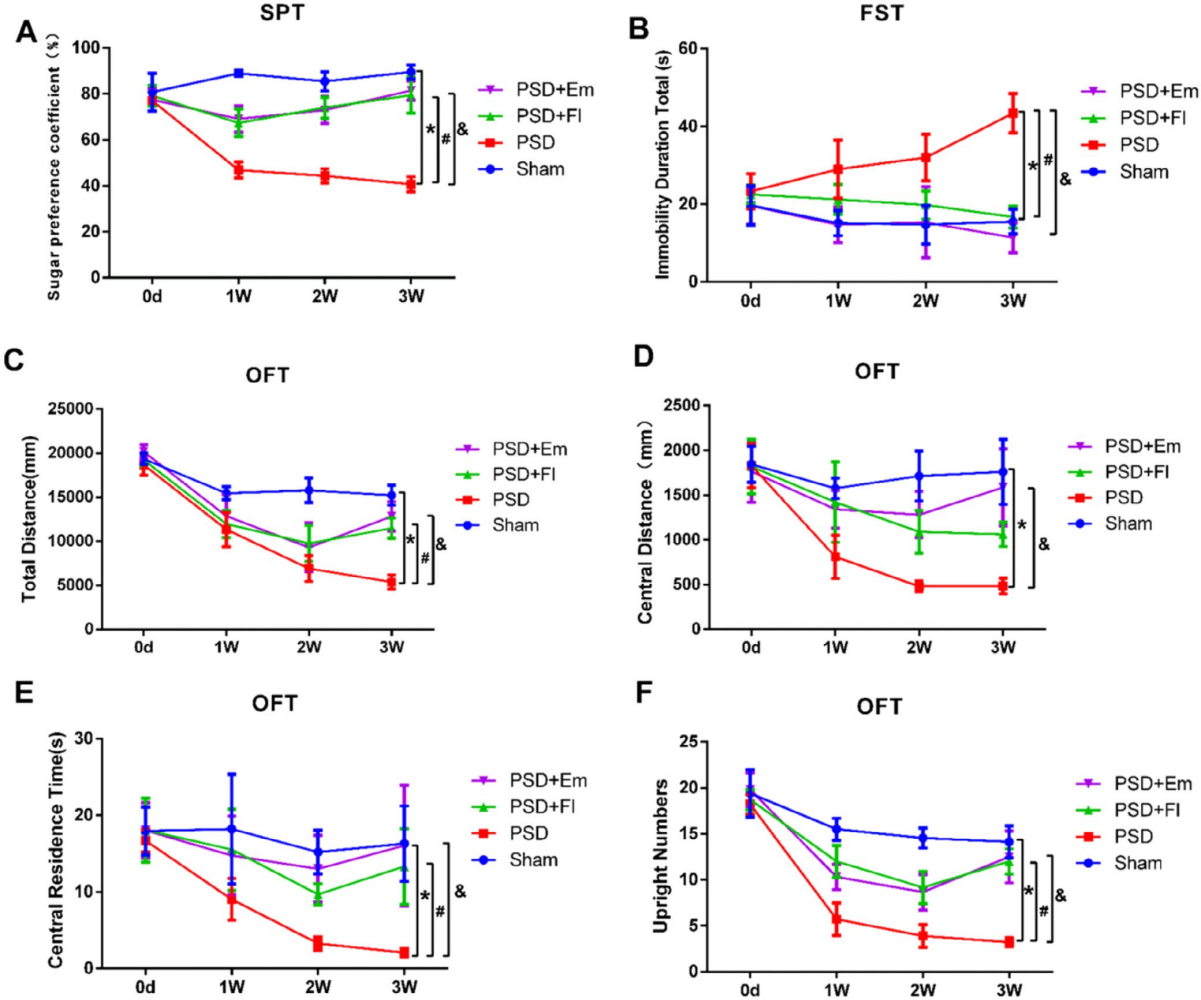
The mitigating effect of Em on depression like behavior in PSD rats over time. (A) The effects of emodin on SPT in PSD rats. (B) The effects of emodin on FST in PSD rats. (C–F) The effects of emodin on OFT in PSD rats. (C) Changes in total travel distance. (D) Distance traveled in the Central Area. (E) Time spent in the Central Area. (F) Frequency of rearing. * comparisons between the PSD group and the Sham group, # comparisons between the PSD + Fl group and the PSD group, & comparisons between the PSD + Em group and the PSD group. *p* < 0.05. *n* = 9.

**FIGURE 4:**
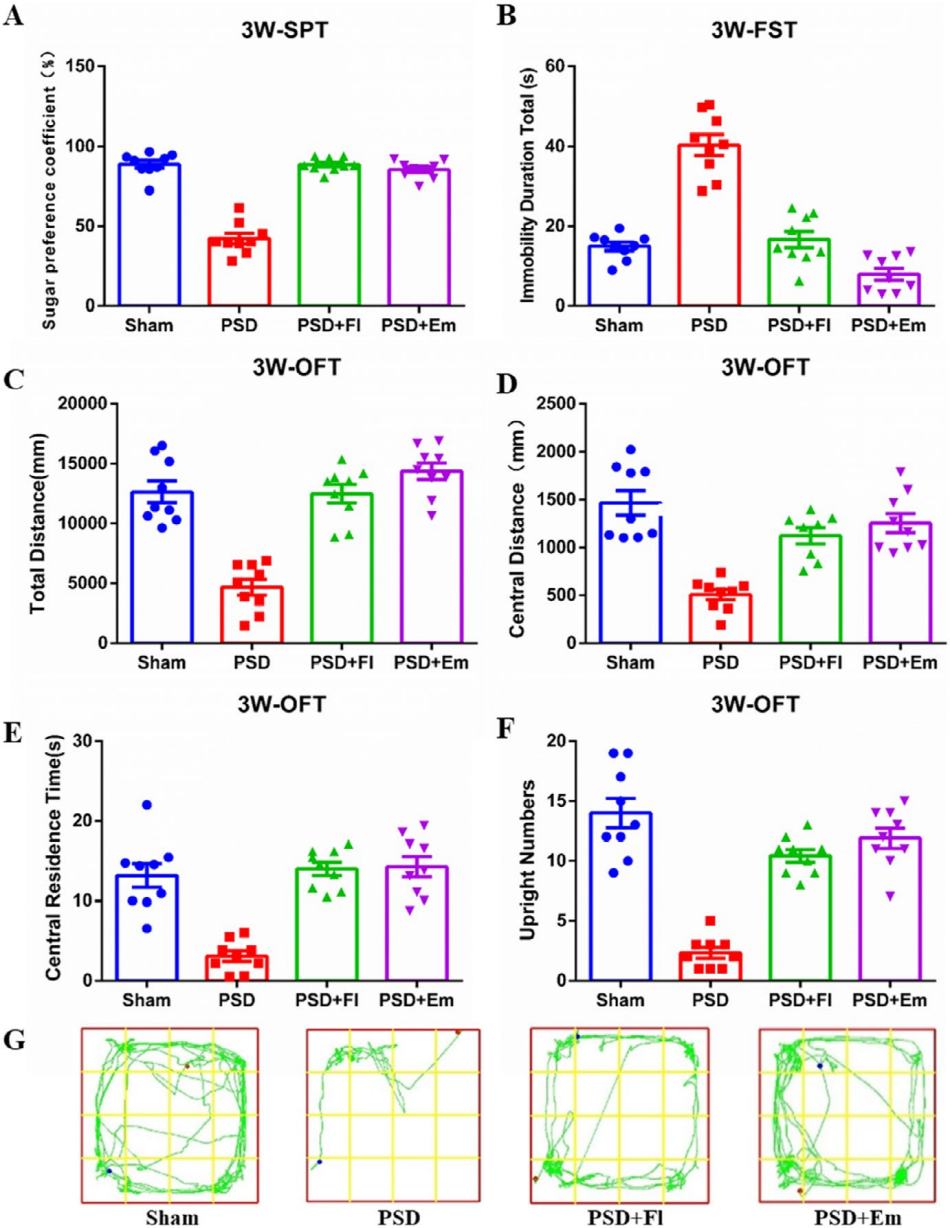
The mitigating effect of Em on depression like behavior in PSD rats at the end of the third week. (A) The effects of emodin on SPT in PSD rats. (B) The effects of emodin on FST in PSD Rats. (C–F) The effects of emodin on OFT in PSD rats. (C) Changes in total travel distance. (D) Distance traveled in the Central Area. (E) Time spent in the Central Area. (F) Frequency of rearing. (G) Trajectory of open field experiment for each group of rats. * comparisons between the PSD group and the Sham group, # comparisons between the PSD + Fl group and the PSD group, & comparisons between the PSD + Em group and the PSD group. *p* < 0.05. *n* = 9.

In the SPT, rats with PSD exhibited a significantly diminished preference for sucrose compared to the Sham group (*p* < 0.001). The OFT revealed that, relative to the Sham group, rats with PSD experienced a reduction in total and central distances, along with central residence time and number of upright postures (*p* < 0.01). The FST results indicated a prolonged immobility time in the model group compared to the Sham group (*p* < 0.05), confirming the successful establishment of the depression model. However, Em intervention significantly counteracted these effects (*p* > 0.05), demonstrating its strong antidepressant efficacy (Figures 3 and 4).

### 3.3 Network Pharmacology and Molecular Docking Predicted Potential Targets and Mechanisms of Action for Em Therapy in PSD

**FIGURE 5:**
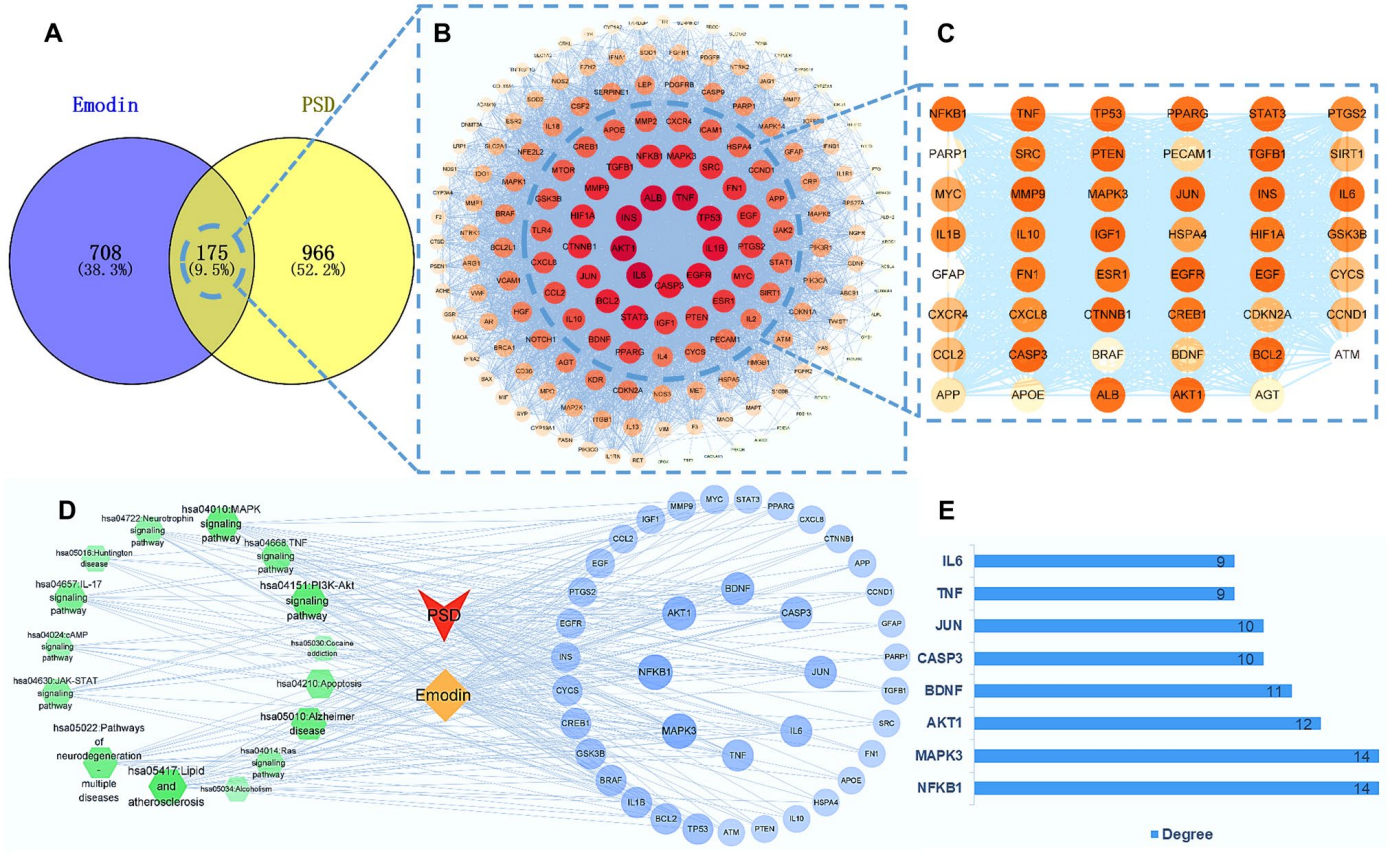
Potential and core targets of Em anti PSD. (A) Venn diagram of potential therapeutic targets. (B) The protein–protein interaction network of Em targeted therapy for PSD. The color of nodes reflects the degree of connection, with more red indicating a higher degree of connection. (C) Key targets identified through PPI network screening. (D) The “component disease target pathway” network. The orange diamond represents Em. The red star represents PSD. The green hexagon on the left represents the signal pathway. The blue circle represents the target on the right. (E) The top 8 core targets in terms of degree.

**FIGURE 6:**
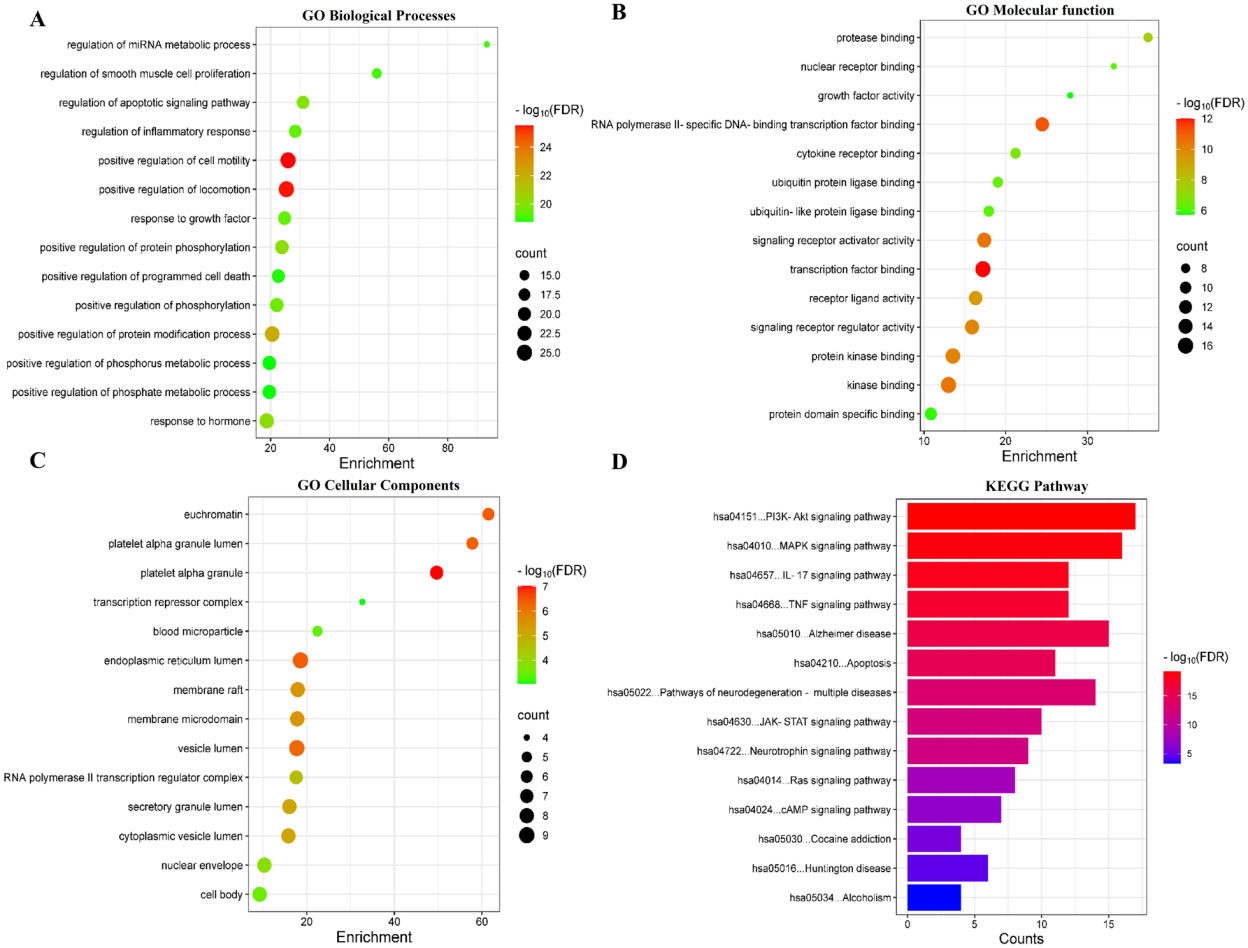
Potential mechanism prediction of Em treatment for PSD. Bubble chart of the top 15 of the enriched (A) biological processes, (B) molecular functions and (C) cellular components linked to the therapeutic effect of Em on PSD. (D) Bar chart of the top 15 of KEGG pathway analysis of the therapeutic targets of Em on PSD.

To further explore the molecular mechanisms by which Em treats PSD, 883 Em‐related targets and 1041 PSD‐related targets were retrieved from relevant databases. Through cross‐analysis, 175 potential targets of Em for the treatment of PSD were identified (Figure 5A). Subsequently, these 175 targets were uploaded to the STRING database to obtain the corresponding protein–protein interaction information (Figure 5B), and 47 key targets were screened (Figure 5C). Subsequently, GO enrichment analysis revealed that these targets are involved in biological processes such as the positive regulation of cell migration, cell motility, locomotion, protein modification processes, protein phosphorylation, and regulation of the apoptotic signaling pathway (Figure 6A).

In terms of molecular function, these targets were primarily associated with DNA‐binding transcription factor binding, transcription factor binding, RNA polymerase II‐specific DNA‐binding transcription factor binding, and kinase binding (Figure 6B). Regarding cellular components, the targets were predominantly enriched in the transcription regulator complex, platelet alpha granule, euchromatin, endoplasmic reticulum lumen, vesicle lumen, and membrane rafts (Figure 6C). KEGG enrichment analysis revealed that these targets are closely related to lipid and atherosclerosis, the PI3K‐Akt, MAPK, IL‐17, and the TNF signaling pathways, Alzheimer's disease, apoptosis, pathways of neurodegeneration involving multiple diseases, and the neurotrophin signaling pathway (Figure 6D).

**FIGURE 7:**
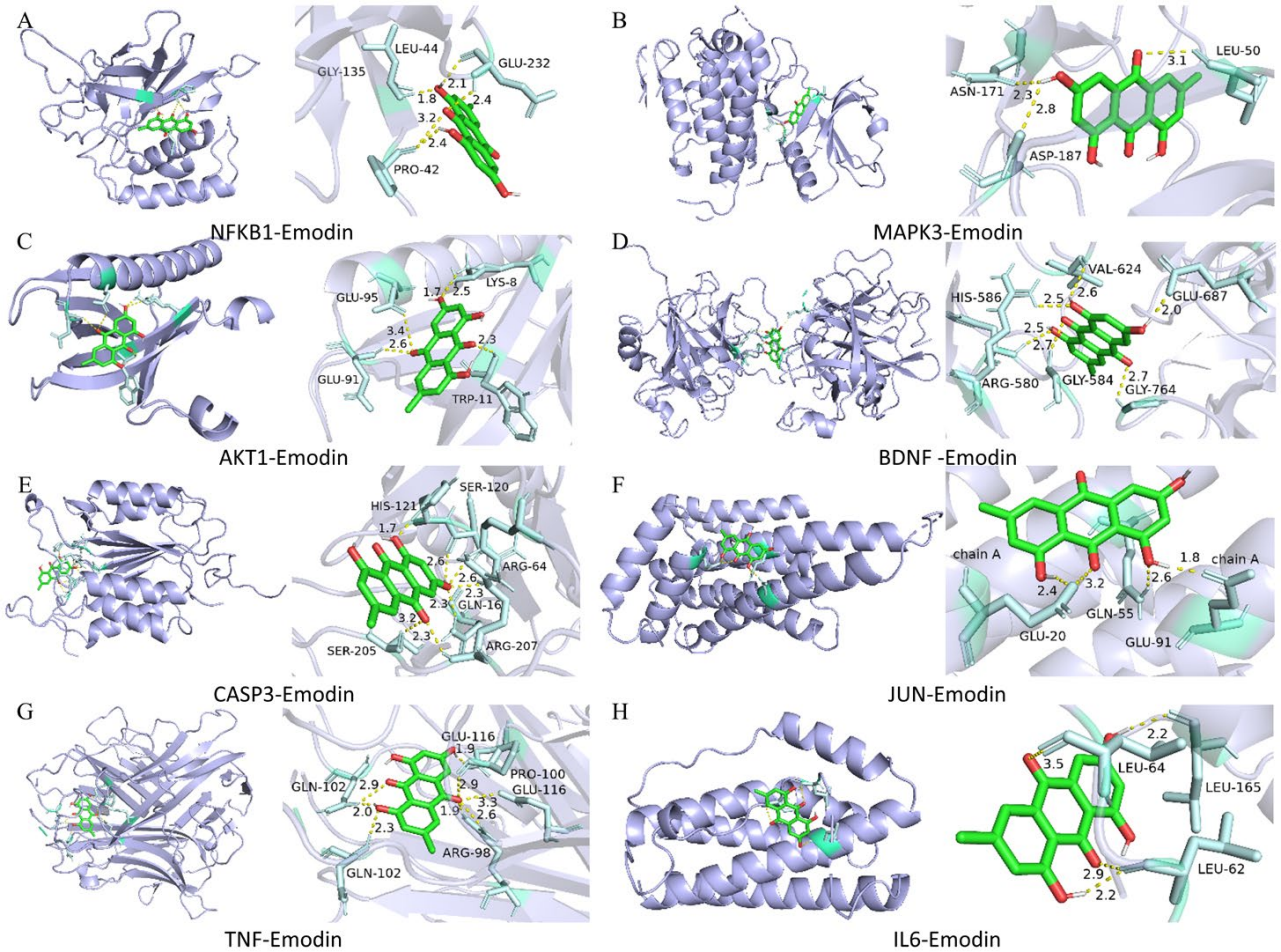
Verification of molecular docking. (A–H) Three‐dimensional map of the binding site between Em and the target protein. Em is displayed in green. The target protein is represented in blue. The location where Em connects to the target protein is represented in blue, indicating a specific docking site between Em and the target protein.

To further investigate the interactions between Em and key targets, the top 15 enriched signaling pathways were selected for study, and Cytoscape software (version 3.9.0) was used to construct the Em‐PSD‐target‐pathway network (Figure 5D). After removing duplicates, 39 potential drug targets were identified (Figure 5D), and the top 8 core targets with the highest degree of enrichment were identified (Figure 5E). Additionally, using AutoDock Tools 1.5.7, the binding capabilities of Em to target proteins were predicted, and the docking models with the lowest binding energies were recorded (Figure 7). The results indicated that Em exhibited a strong and stable binding affinity for NFKB1, MAPK3, AKT1, BDNF, CASP3, JUN, tumor necrosis factor (TNF), and interleukin‐6 (IL‐6). Figure 7A–H display the specific docking sites of Em on the target proteins. All receptor‐ligand binding energies were less than −5 kcal/mol, suggesting good binding activity between the compound and the targets, with BDNF displaying the highest binding affinity (Table 3).

**TABLE 3:** Molecular docking information and binding energy.

Active ingredient	Mol id	Target name	PDB id	Structure	Binding energy (Kcal/mol)
Emodin	MOL000472	NFKB1	8TQD	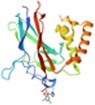	−4.68
		MAPK3	7NRB	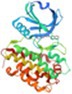	−6.22
		AKT1	1UNQ	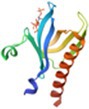	−4.79
		BDNF	8F7U	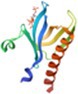	−6.45
		CASP3	2CJX	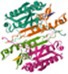	−5.08
		JUN	6Y3V	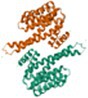	−4.61
		TNF	2E7A	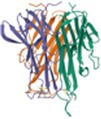	−5.62
		IL6	1ALU	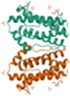	−4.43

### 3.4 Protective Effect of Em on Apoptosis in Hippocampus and mPFC of Rats With PSD

**FIGURE 8:**
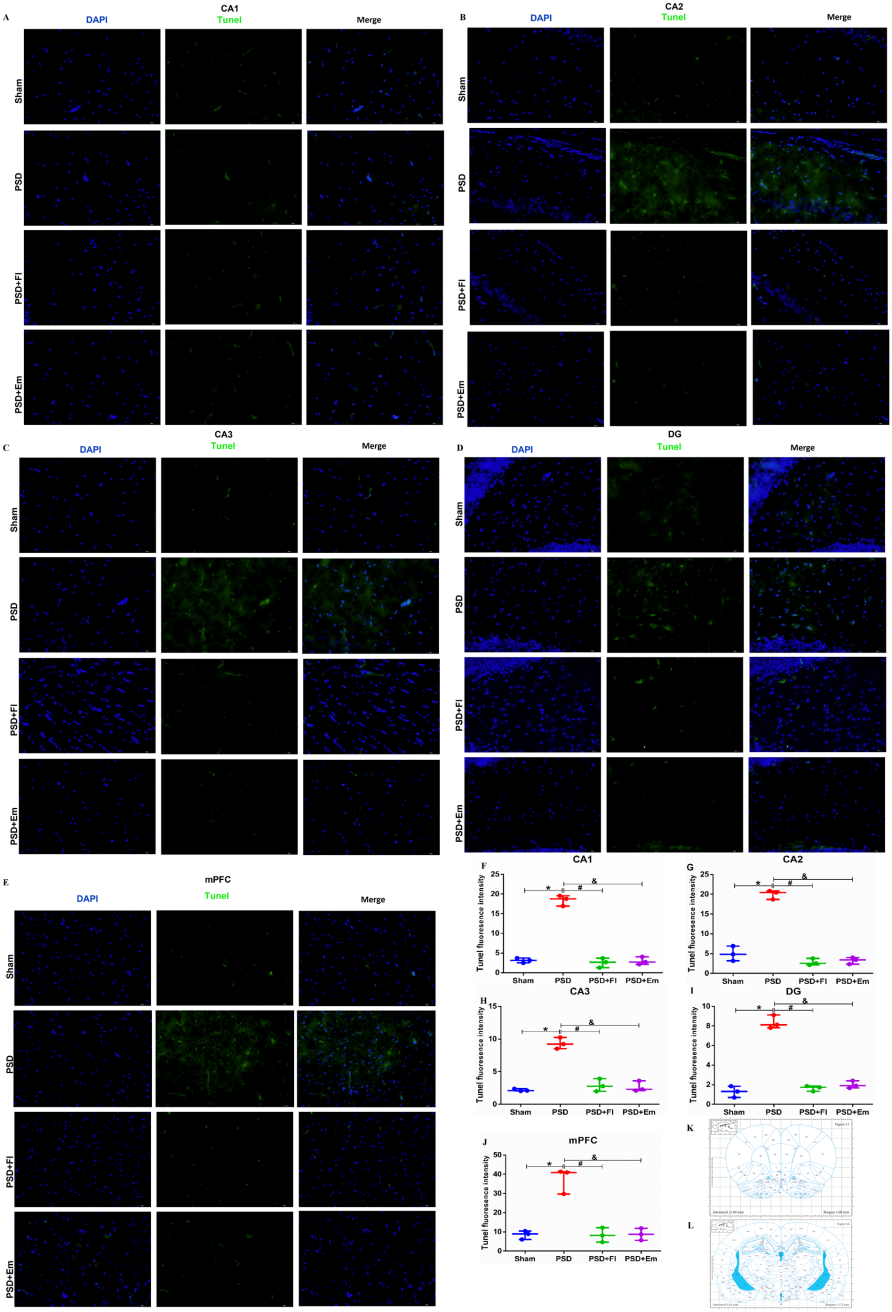
Protective effect of Em on apoptosis in hippocampus and mPFC of PSD rats. (A) Tunel‐stained fluorescence images of the hippocampal CA1 region. (B) Tunel‐stained fluorescence images of the hippocampal CA2 region. (C) Tunel‐stained fluorescence images of the hippocampal CA3 region. (D) Tunel‐stained fluorescence images of the hippocampal DG region. (E) Tunel‐stained fluorescence images of the mPFC. (F) Tunel‐stained fluorescence intensity in the hippocampal CA1 region. (G) Tunel‐stained fluorescence intensity in the hippocampal CA2 region. (H) Tunel‐stained fluorescence intensity in the hippocampal CA3 region. (I) Tunel‐stained fluorescence intensity in the hippocampal DG region. (J) Tunel‐stained fluorescence intensity in the mPFC. Blue fluorescence represents DAPI staining, green fluorescence represents TUNEL staining. (K) Brain atlas of hippocampal in rats. (L) Brain atlas of mPFC in rats.* comparisons between the PSD group and the Sham group, # comparisons between the PSD + Fl group and the PSD group, & comparisons between the PSD + Em group and the PSD group. *p* < 0.05. *n* = 3.

Based on network pharmacology predictions, we selected the apoptosis and neurotrophic factor pathways for further investigation. TUNEL staining was used to detect cellular apoptosis, and the results (Figure 8) displayed that compared with the Sham group, the TUNEL fluorescence intensity in the hippocampal CA1, CA2, CA3, DG, and mPFC of the model group was significantly increased (*p* < 0.01/*p* < 0.05/*p* < 0.05/*p* < 0.01/*p* < 0.001). However, pretreatment with Fl and Em significantly reduced this intensity (*p* < 0.01/*p* < 0.05/*p* < 0.05/*p* < 0.05/*p* < 0.01), restoring it to near‐normal levels. These findings suggest that Em exerts a protective effect against apoptosis in the hippocampus and mPFC of rats with PSD.

### 3.5 Effects of Em on the Expression of PSD Target Gene BDNF and Its Isoforms proBDNF and mBDNF

**FIGURE 9:**
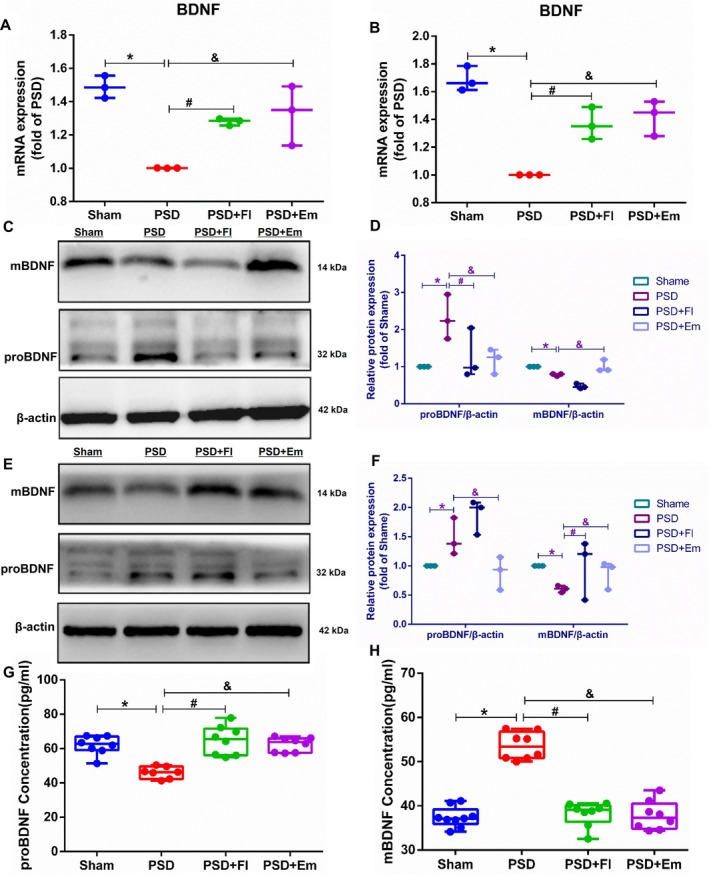
Effects of Em on the expression of PSD target gene BDNF and its isoforms proBDNF and mBDNF. (A) The impact of Emodin on BDNF mRNA expression in the hippocampal region of PSD rats, *n* = 3. (B) The effect of Emodin on BDNF mRNA expression in the mPFC of PSD rats, *n* = 3. (C, D) The influence of Emodin on the protein expression of proBDNF and mBDNF in the hippocampal region of PSD rats, *n* = 3. (E, F) The effect of Emodin on the protein expression of proBDNF and mBDNF in the mPFC of PSD rats. (G, H) The influence of Emodin on the serum levels of proBDNF and mBDNF in PSD rats, *n* = 8. * comparisons between the PSD group and the Sham group, # comparisons between the PSD + Fl group and the PSD group, & comparisons between the PSD + Em group and the PSD group. *p* < 0.05.

The key target protein with the strongest binding energy, BDNF, was selected, and qRT‐PCR was used to assess its mRNA levels in the hippocampus (*p* < 0.001) and mPFC (*p* < 0.001). Compared with the Sham group, the mRNA levels in the hippocampus and mPFC of the PSD group were significantly reduced but markedly elevated following pretreatment with Fl or Em (*p* < 0.05) (Figure 9A,B). BDNF exists in two forms: ProBDNF and mBDNF. Accordingly, we used WB to detect the protein expression of proBDNF and mBDNF in the hippocampus and mPFC of the rats. Relative to the Sham group, the PSD group exhibited significantly increased proBDNF protein expression (*p* < 0.001/*p* < 0.001) and decreased mBDNF protein expression (*p* < 0.001/*p* < 0.001) in the hippocampus and mPFC. Pretreatment with Em led to a significant reduction in proBDNF protein expression (*p* < 0.05/*p* < 0.05) and a corresponding increase in mBDNF protein expression (*p* < 0.05/*p* < 0.05) in the hippocampus and mPFC (Figure 9C–F). The serum levels of proBDNF and mBDNF mirrored the protein expression trends observed in the hippocampus (Figure 9G,H). These findings suggest an imbalance in the proBDNF/mBDNF ratio in the hippocampus and mPFC of rats with PSD, which can be modulated and restored to equilibrium by Em.

### 3.6 Effects of Em on the Expression of Key Cleavage Enzymes tPA, MMP‐9, Furin, and PC Genes and Proteins in Regulating proBDNF/mBDNF Balance in the Hippocampus and mPFC of Rats With PSD

**FIGURE 10:**
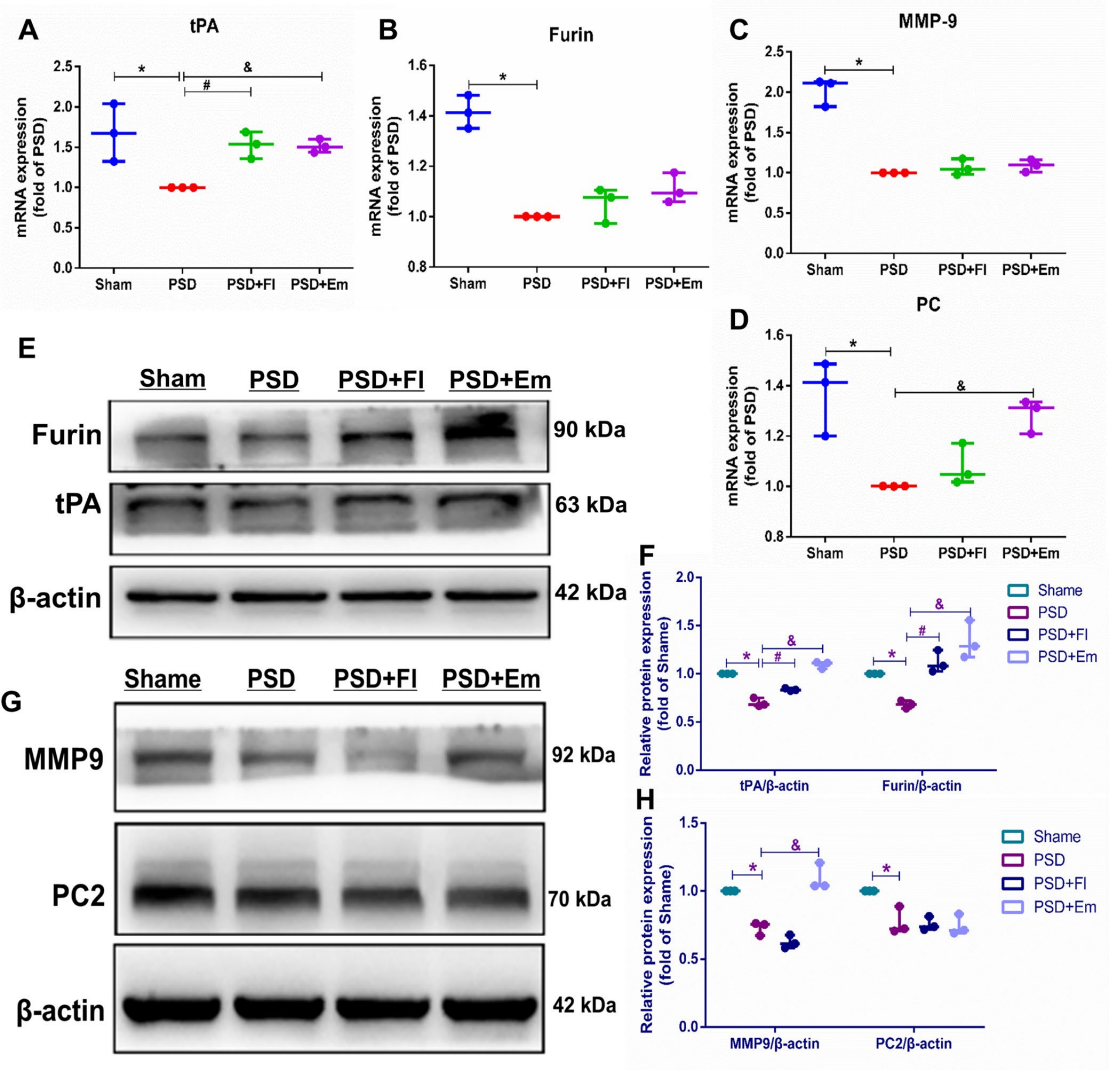
Effects of emodin on the expression of tPA, MMP9, furin, and PC genes and proteins in the hippocampus of PSD rats. (A–D) Changes in tPA, MMP9, furin, and PC mRNA levels in the rat hippocampus. (E–H) Alterations in the expression of tPA, MMP9, furin, and PC proteins in the hippocampus. * comparisons between the PSD group and the Sham group, # comparisons between the PSD + Fl group and the PSD group, & comparisons between the PSD + Em group and the PSD group. *p* < 0.05. *n* = 3.

**FIGURE 11:**
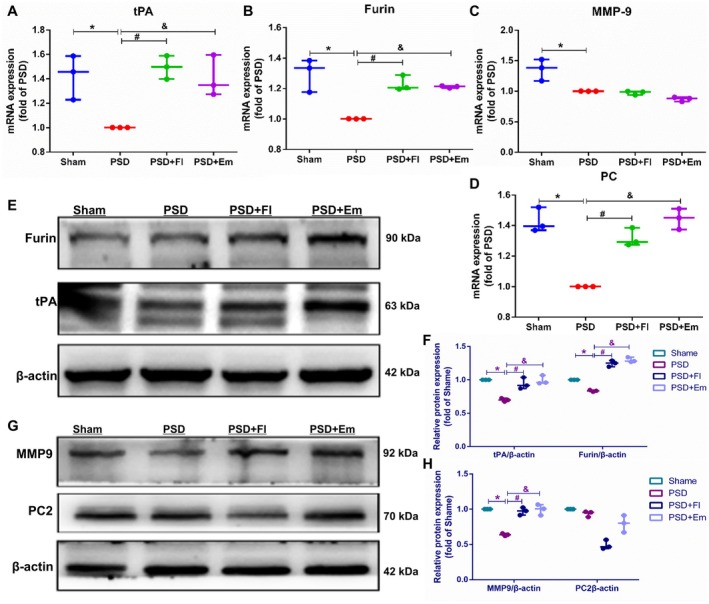
Effects of emodin on the expression of tPA, MMP9, furin, and PC genes and proteins in the mPFC of PSD rats. (A–D) Changes in mRNA levels of tPA, MMP9, furin, and PC in the mPFC. (E–H) Alterations in the expression of tPA, MMP9, furin, and PC proteins in the mPFC. * comparisons between the PSD group and the Sham group, # comparisons between the PSD + Fl group and the PSD group, & comparisons between the PSD + Em group and the PSD group. *p* < 0.05. *n* = 3.

To further demonstrate the mechanisms by which Em regulates the proBDNF/mBDNF balance in rats with PSD, we employed qRT‐PCR and WB to assess the impact of Em on the expression of key cleavage enzymes tPA, MMP‐9, Furin, and PC genes and proteins in the hippocampus and mPFC. The results revealed that, compared to the Sham group, the PSD group exhibited significantly reduced expressions of tPA, MMP‐9, Furin, and PC mRNA and proteins in the hippocampus and mPFC [mRNA in hippocampus: (*p* < 0.05/*p* < 0.001/*p* < 0.05/*p* < 0.01); protein in hippocampus: (*p* < 0.01/*p* < 0.01/*p* < 0.01/*p* < 0.01); mRNA in mPFC: (*p* < 0.05/*p* < 0.05/*p* < 0.01/*p* < 0.001); protein in mPFC: (*p* < 0.01/*p* < 0.001/*p* < 0.001)], with a less pronounced decrease in PC2 protein expression (*p* > 0.05) in the mPFC. Following the Em intervention, there was a significant increase in the expression of tPA and PC mRNA in both the hippocampus (*p* < 0.05/*p* < 0.05) and mPFC (*p* < 0.05/*p* < 0.05), as well as a marked elevation in Furin mRNA expression in the mPFC (*p* < 0.05). However, no significant differences were observed in the expression of MMP‐9 and Furin mRNA in the hippocampus or MMP‐9 mRNA in the mPFC (*p* > 0.05/*p* > 0.05/*p* > 0.05) (Figures 10A–D and 11A–D).

Em treatment also resulted in a significant increase in the expression of tPA, MMP‐9, and Furin proteins in the hippocampus (*p* < 0.05/*p* < 0.05/*p* < 0.05) and mPFC (*p* < 0.001/*p* < 0.001/*p* < 0.001), whereas PC protein levels remained unchanged (*p* > 0.05) (Figures 10E–H and 11E–H). These findings indicate that tPA, MMP‐9, Furin, and PC mRNA and protein expression levels were markedly reduced in the hippocampus and mPFC of rats with PSD. Em can concomitantly upregulate the expression of tPA genes and proteins in the hippocampus and mPFC, as well as enhance Furin gene and protein expression in the mPFC, although its regulatory effects on other cleavage enzymes at gene and protein levels were inconsistent. As a result, we speculate that Em may promote the conversion of proBDNF to mBDNF by augmenting tPA expression in the hippocampus and tPA and Furin in the mPFC, thereby restoring the proBDNF/mBDNF balance, reducing apoptosis, and ameliorating PSD states.

## 4 Discussion

In this study, we established a PSD rat model using MCAO combined with SI and CUMS, demonstrating that Em has a definitive therapeutic efficacy against PSD. Network pharmacology and molecular docking were employed to explore the multifaceted mechanisms by which Em exerts anti‐PSD effects and to predict and analyze key signaling pathways and targets. Finally, the effects and mechanisms of Em on PSD were validated in animal experiments. The research workflow is illustrated in Figure 1. This study suggests that the therapeutic mechanism of Em in PSD may involve regulating the proBDNF/mBDNF balance through tPA and Furin, thereby reducing apoptosis and exerting an antidepressant effect.

PSD is a leading cause of poor recovery, diminished quality of life, and suboptimal rehabilitation outcomes in patients [53]. The quality of life of patients with PSD is significantly lower than that of patients with a simple stroke, particularly in the domains of cognition, emotion, limb function, and social functioning [54]. Currently, despite the drawbacks of drug withdrawal and side effects, pharmacotherapy remains the primary treatment approach for PSD [55].

Em, a member of the anthraquinone class of quinones, is richly present in species from three distinct plant families: Fabaceae (*Cassia* spp.), Polygonaceae (*Rheum, Rumex*, and *Polygonum* spp.), and Rhamnaceae (*Rhamnus, Frangula*, and *Ventilago* spp.). Numerous studies have demonstrated that Em provides therapeutic support for both cerebral ischemia and hemorrhage through various interconnected mechanisms and has been identified as a promising lead compound for the development of antidepressant drugs [56]. However, the effects and mechanisms of Em in PSD treatment have not yet been fully understood.

Animal models have been extensively used to investigate behavioral and emotional changes [57]. The combined method of MCAO, SI, and CUMS is a classical paradigm for PSD models that effectively simulates the post‐stroke depressive states observed in clinical settings. This study discovered that this paradigm can successfully establish a PSD rat model, and for the first time, it has been demonstrated that Em, Fl can significantly enhance neural function and alleviate depressive symptoms in PSD, and the improvement effect of Em seems to be even better. In addition, Em can also improve neurological damage, which Fl does not have. But none of them improved the volume of cerebral infarction. Previous studies have also shown that Fl can improve depressive‐like behavior [50, 51], while Em can alleviate depressive‐like behavior [44] and improve neurological damage [43].

Although Em improved neurological function, it did not reduce infarct volume, suggesting that its therapeutic effects may target post‐ischemic neuronal reorganization rather than acute necrosis. This is supported by: (i) Emodin promotes axonal regeneration via BDNF upregulation, potentially underpinning its role in stroke recovery [11]; (ii) The critical window for infarct volume determination occurs within 24–48 h post‐ischemia, whereas functional/behavioral recovery relies on neuroplasticity—a process spanning weeks and often independent of acute infarct volume. Notably, [58] Em exhibits neuroprotection by suppressing oxidative stress and apoptosis [41] Em reduces serum corticosterone levels and improves behavioral performance in depressed mice. Furthermore, it upregulates BDNF and glucocorticoid receptor (Santarelli et al.) expression while alleviating anhedonia (as evidenced by increased sucrose preference).

While both emodin and fluoxetine improve depressive behaviors, their mechanisms likely differ. Fluoxetine, as a selective serotonin reuptake inhibitor (SSRI), primarily increases synaptic serotonin levels by blocking SERT [59]. In contrast, our data suggest emodin's effects are mediated through tPA/Furin‐regulated proBDNF/mBDNF balance (Figures 3 and 4), representing a pathway independent of monoaminergic neurotransmission. This aligns with previous studies showing that natural anthraquinones like emodin modulate neurotrophic signaling, exerting antidepressant effects through the tPA‐BDNF–TrkB pathway rather than directly targeting the monoamine system [60, 61, 62]. Key studies demonstrating tPA/plasmin system regulation of proBDNF to mBDNF conversion and its impact on synaptic plasticity are cited [63].

The mechanistic differences may explain why emodin demonstrated faster onset than fluoxetine in our behavioral tests. While fluoxetine typically requires weeks to achieve therapeutic effects through synaptic adaptation (the rapid synaptic 5‐HT increase by SSRIs shows a temporal mismatch with behavioral improvement (2–4 weeks) [64]), and needs chronic administration to induce hippocampal neurogenesis [65], emodin rapidly upregulates Furin and mBDNF expression, enhancing synaptic plasticity within 24 h [66]. In CUMS models, emodin improved depressive behaviors within 3 days, compared to 14 days for fluoxetine [60]. This suggests emodin's potential utility in treatment‐resistant depression cases where SSRIs prove ineffective.

Network pharmacology is an emerging discipline that combines systems biology, multidirectional pharmacology, computational biology and network science. It aims to study the interaction between drugs and biological systems from a holistic and systematic perspective. It reveals the complex mechanism of drug action by building a multi‐dimensional network of “drug target disease gene”, and provides new ideas for drug research and development, new use of old drugs and personalized treatment. Recently, network pharmacology and molecular docking methods have been widely employed to predict the targets and mechanisms of pharmacological substances in TCM [67]. Based on the premise that Em possesses anti‐post‐stroke depressive effects, we utilized network pharmacology and molecular docking techniques to predict core targets, including NFKB1, MAPK3, AKT1, BDNF, CASP3, JUN, TNF, and IL6, with BDNF being identified as the target with the highest binding affinity. Potential pathways were also predicted, including those involved in lipid metabolism, atherosclerosis, PI3K‐Akt signaling, MAPK, IL‐17, TNF, neurotrophin signaling, apoptosis, Alzheimer's disease, and other neurodegenerative diseases. NFKB1 plays a pivotal role in inflammatory diseases and cancer. MAPK3 may be a critical target for spinal cord ischemia–reperfusion injury [68].

CASP3 functions as a central executor of apoptosis, and its gene expression is positively correlated with both the duration of illness and frequency of depressive episodes [69]. The transcription factor c‐JUN has been demonstrated to either induce or inhibit apoptosis [70] and may participate in the repair of neural injuries [71]. The expression of BDNF and MAPK3 plays a significant role in the therapeutic approach for PSD. Notably, PI3K/AKT and MAPK pathways are important biomarkers for PSD and can be targeted for therapeutic interventions through acupuncture [72]. In patients with PSD, inflammatory markers such as IL‐6 and TNF‐α are markedly elevated in the bloodstream [73], and the combined detection of these biomarkers can effectively predict the onset of PSD.

Based on our findings, we hypothesize that Em influences inflammatory regulation by modulating the expression of inflammatory factors, such as NFKB1, IL‐6, and TNF‐α, thereby impacting the inflammation‐associated signaling pathways of PI3K‐Akt, MAPK, IL‐17, and TNF. Furthermore, it may regulate the expressions of CASP3 and c‐JUN in the endoplasmic reticulum and vesicular compartments, thereby influencing apoptotic signaling pathways and processes related to apoptosis and neural injuries. Additionally, Em may affect the expression of BDNF on membrane rafts, thereby influencing protein modification processes and neurotrophin signaling pathways, ultimately contributing to neuroprotection and ameliorating PSD. The molecular docking verification results further demonstrated that Em exhibited good binding ability with the aforementioned core targets, with the strongest binding observed with BDNF, suggesting that Em can stably bind to the receptor to exert its anti‐PSD effects. Finally, this study selected BDNF as the target to verify the expression of Em before and after the PSD intervention. The results demonstrated that SMY enhanced BDNF gene expression and proBDNF/mBDNF protein expression in rats with PSD. Consequently, this study specifically investigated the neurotrophic signaling pathways and apoptosis associated with BDNF.

PSD and poor neurological prognosis may be directly linked to neuronal damage or apoptosis after stroke. Clinically, frontal lobe infarction is the most common type of stroke associated with PSD [74]. This correlation likely arises from the close relationship between the frontal lobe and critical functions such as emotion, cognition, and memory. The frontal lobe plays a pivotal role in emotional processing and maintains extensive neural connections with various brain regions, including the thalamus and hippocampus [75]. Research has demonstrated a reduction in neural innervation in the mPFC and hippocampus of mice with PSD [76]. In our study, we observed a significant increase in apoptotic cells in the hippocampus and mPFC of rats with PSD, whereas Em, Fl significantly reduced the number of apoptotic cells, suggesting a protective effect against apoptosis in these brain regions.

BDNF is the most abundant and widely distributed neurotrophin in the central nervous system and is predominantly expressed in the hippocampus and cerebral cortex [77]. Neurotrophins play a crucial role in mediating behavioral responses to antidepressants [78], and enhancing BDNF expression in the brain may alleviate PSD symptoms [48]. Our research demonstrated a significant downregulation of BDNF mRNA levels in the hippocampus and mPFC of rats with PSD, while Em, Fl effectively corrected the abnormally low BDNF mRNA levels in these brain regions. BDNF is primarily synthesized in neurons and glial cells and exists in two forms: ProBDNF and mBDNF. Our findings demonstrated a significant upregulation of proBDNF protein expression and a notable downregulation of mBDNF protein expression in the hippocampus, mPFC, and serum of rats with PSD, leading to an imbalance between proBDNF and mBDNF levels. Em, Fl successfully rectified this imbalance and restored homeostasis. Previous studies have indicated that a significant reduction in the mBDNF/proBDNF ratio in the ischemic PFC and hippocampus plays a key role in PSD [9]. High‐intensity interval training has been found to more effectively increase the mBDNF/proBDNF ratio in the ischemic hippocampus, enhancing neural plasticity and improving depression‐like behaviors in rats with PSD [11]. These findings align with the results of the present study, suggesting that PSD may be associated with an imbalance between proBDNF and mBDNF and that Em may exert its anti‐PSD effects by restoring the equilibrium between these two forms.

The product of the BDNF gene is initially proBDNF, which, upon release, can be catalyzed into mBDNF by intracellular proteases, such as Furin or PC. This cleavage process allows mBDNF to be secreted extracellularly and exert its effects. Alternatively, proBDNF can be cleaved into mBDNF by extracellular proteases, such as matrix MMP‐9 or tPA [79]. Although mBDNF is generated through the cleavage of proBDNF, these two forms exert vastly different effects when they bind to different receptors [80]. mBDNF selectively associates with the TrkB receptor, activating signaling pathways such as PI3K/Akt, MAPK/ERK, and PLCγ/CaMK, which promote axonal growth, synaptic remodeling, and neuronal protection. In contrast, proBDNF preferentially forms a complex with p75NTR and sortilin, leading to neuronal apoptosis and the collapse of their outgrowth [81]. Consequently, this study suggests that the regulatory influence of BDNF on neuronal morphology and function is ultimately determined by alterations in the proBDNF/mBDNF ratio, which is modulated by proteolytic enzymes. To further investigate this, we assessed the gene and protein levels of Furin, PC, tPA, and MMP‐9 in the hippocampus and mPFC of rat brains.

Our findings revealed a significant downregulation of tPA and Furin protein and gene expression in the hippocampus and mPFC of rats with PSD, whereas Em and Fl notably upregulated both tPA and Furin expressions. Fl did not induce the reversal of MMP9 protein expression and PC mRNA expression in the hippocampus like Em. Previous research has indicated that the proBDNF/mBDNF ratio is elevated in the hippocampus of depressed rats, accompanied by decreased tPA levels and cognitive decline [82]. Additionally, studies have demonstrated that PSD induces a reduction in tPA, mBDNF, and TrkB expression, with the protective effects of acupuncture against depression‐like behaviors induced by PSD being counteracted by the tPA‐specific inhibitor PAI‐1 [83]. These findings are consistent with those of the present study. Notably, no prior research has reported the involvement of Furin, MMP‐9, or PC2 in PSD. This study is the first to demonstrate a significant downregulation of both Furin gene and protein expression in the hippocampus and mPFC of rats with PSD, with Em enhancing Furin and tPA expression in these regions. In conclusion, Em may exert its anti‐PSD effects by upregulating Furin and tPA expression in the hippocampus and mPFC, thereby regulating the proBDNF/mBDNF balance.

Although our data suggests that the antidepressant effect of emodin involves tPA/Furin mediated pre‐BDNF cleavage, further research is needed to determine whether emodin directly binds to these enzymes or regulates their transcription/activity. Biochemical assays (such as recombinase kinetics) and conditional knockout models will help establish causal relationships. This study aims to explore the behavioral effects of emodin and its correlation with the tPA/Furin BDNF pathway, rather than analyzing specific molecular interactions. The correlation analysis between behavior and biochemical markers is a reasonable starting point for the mechanism hypothesis [84]. Preliminary research can prioritize the discovery of effects, and specific details of enzyme activity regulation and signal transduction mechanisms need to be validated through gene knockout or in vitro enzymatic experiments in the future.

This study represents the first application of a comprehensive pharmacological strategy to explore the protective effects and mechanisms of Em on PSD. The integrated pharmacological approach combines network pharmacology, molecular docking, and experimental verification. However, our study has certain limitations. We preliminarily verified the potential targets of Em in treating PSD and the possible pathways of action based on the regulation of the proBDNF/mBDNF balance using network pharmacology and initial experimental validation. Research is needed to further demonstrate the specific mechanisms underlying its anti‐PSD effects. Additionally, the results of this study do not provide guidance on the optimal Em dosage for clinical applications. Future pharmacological research is needed to ascertain the ideal dosage and bioavailability of Em for treating PSD. Our study did not evaluate long‐term infarct evolution (e.g., glial scar formation) or dynamic changes in peri‐infarct connectivity (e.g., via fMRI). Future work should employ longitudinal imaging and electrophysiology to assess whether emodin's functional benefits correlate with white matter integrity or network reorganization. A limitation of this study is the lack of direct molecular comparisons (e.g., serotonin vs. BDNF pathway analysis). Future experiments using monoamine depletion models or SSRI‐resistant animals could further elucidate these differential mechanisms. Although the sample size is limited, small sample studies are applicable for preliminary research, and there are plans to expand cohort validation in the future.

## 5 Conclusion

In summary, the process of neuronal apoptosis induced by PSD is complex and multifaceted. This study preliminarily revealed that the protective mechanism of Em on PSD is multi‐target and involves several pathways. Em exerts its therapeutic effects by binding to the core target BDNF. Its specific mechanism of action appears to involve the regulation of proBDNF/mBDNF balance through tPA and Furin, which mitigates apoptosis and exerts therapeutic effects. These findings highlight the critical role of proBDNF/mBDNF balance in PSD progression and provide important evidence that Em may be a promising candidate for treating PSD.

## Author Contributions

**Wei Liu:** writing – review and editing, validation, supervision, funding acquisition, conceptualization. **Xiaoju Liu:** writing – original draft, methodology, formal analysis, data curation. **Jie Gao:** writing – review and editing, supervision, conceptualization. **Kai Yang:** methodology, investigation, formal analysis, data curation. **Weiming Zhu:** visualization, software. **Ming Su:** methodology, investigation. **Zichun Liu:** visualization, validation, software; **Yaxin Yuan:** validation, resources, methodology. **Linya Cao:** methodology, investigation, data curation. **Tong Wu:** validation, methodology.

## Ethics Statement

This study and its experimental procedures have been approved by the Ethics Review Committee for Animal Welfare of Shandong University of Traditional Chinese Medicine (Approval No.: SDUTCM20230314001). All animal housing and experiments were conducted in strict accordance with the regulations of the Ethics Review Committee for Animal Welfare at Shandong University of Traditional Chinese Medicine.

## Conflicts of Interest

The authors declare no conflicts of interest.

## Supporting information


**Data S1:** cns70581‐sup‐0001‐Supinfo.pdf.

## Data Availability

The data that support the findings of this study are available from the corresponding author upon reasonable request.
