# Supplementary material for: Elucidating the Mechanism of Emodin in Treating Post‐Stroke Depression Through Network Pharmacology and Animal Experiments
Source: CNS Neurosci Ther. 2025 Sep 24;31(9):e70581. doi: 10.1111/cns.70581 (PMC12460929; doi:10.1111/cns.70581)

Figure.9 C proBDNF、mBDNF proteins in the Hippocampus

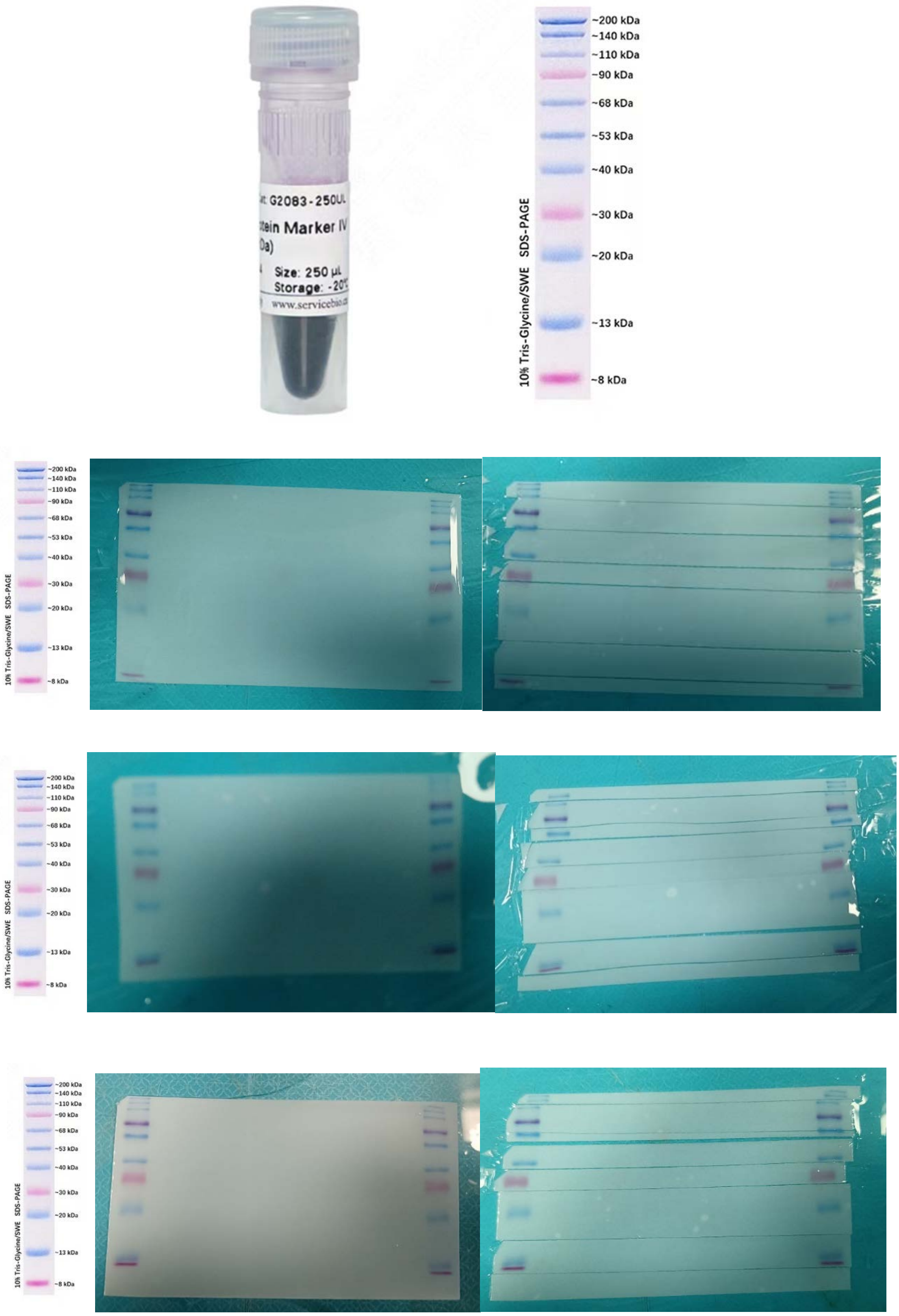

Figure 9C Complete unedited gel/imprint

proBDNF-1

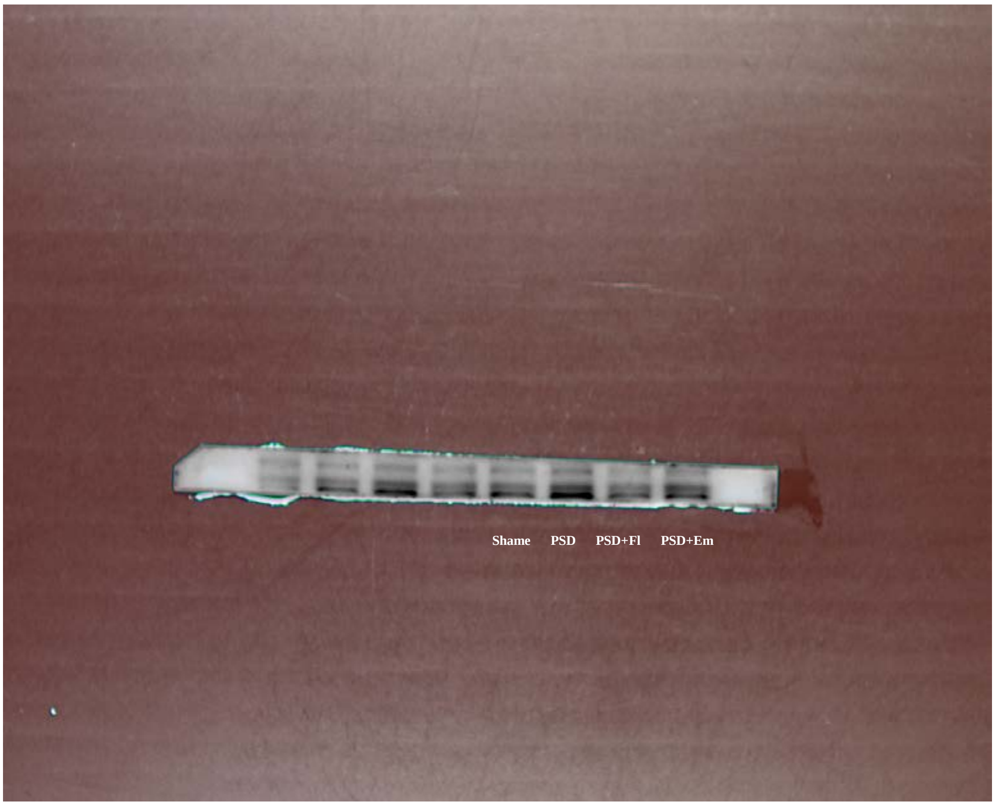

proBDNF-1'

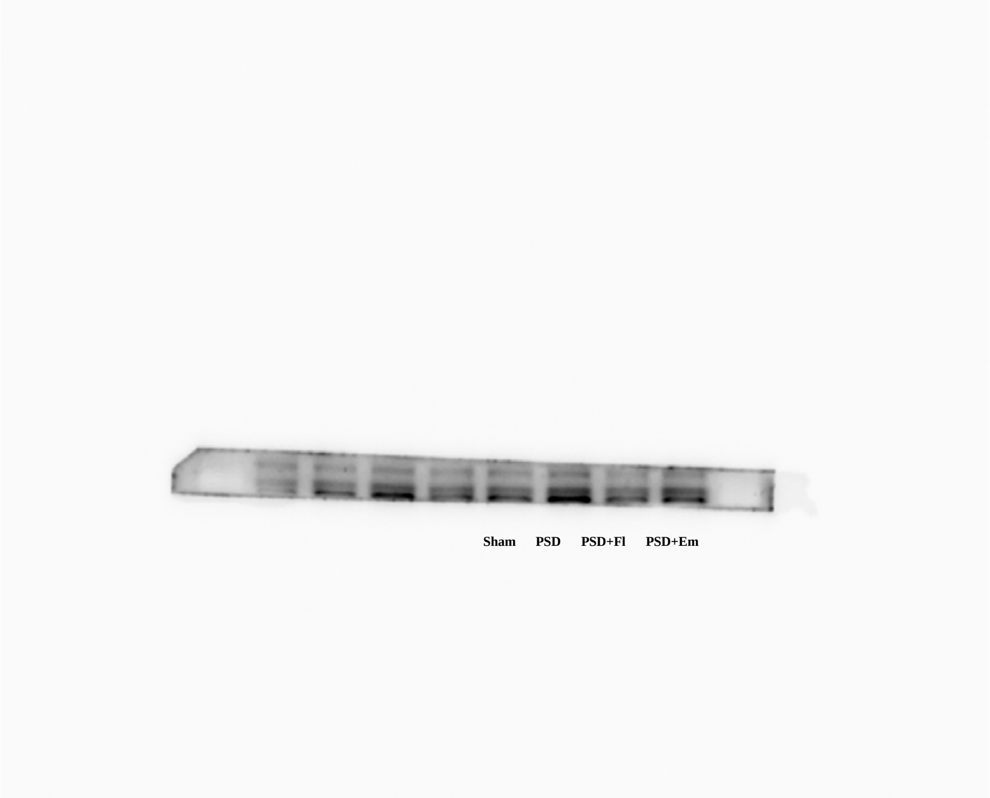

proBDNF-2

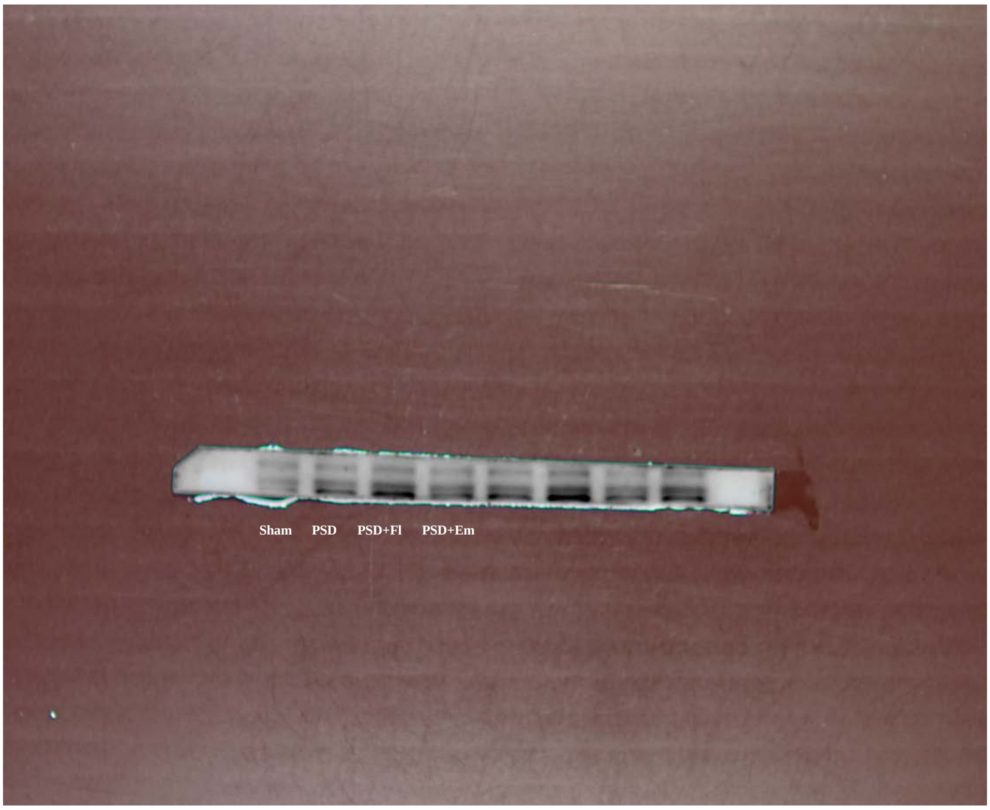

proBDNF-2’

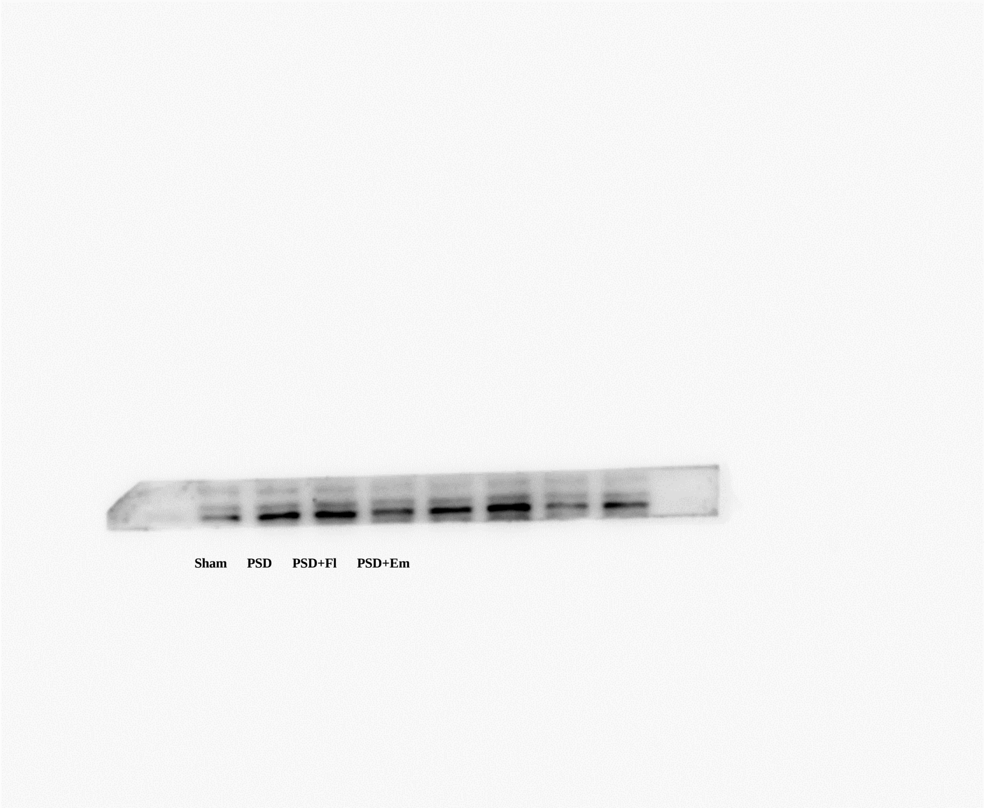

proBDNF-3

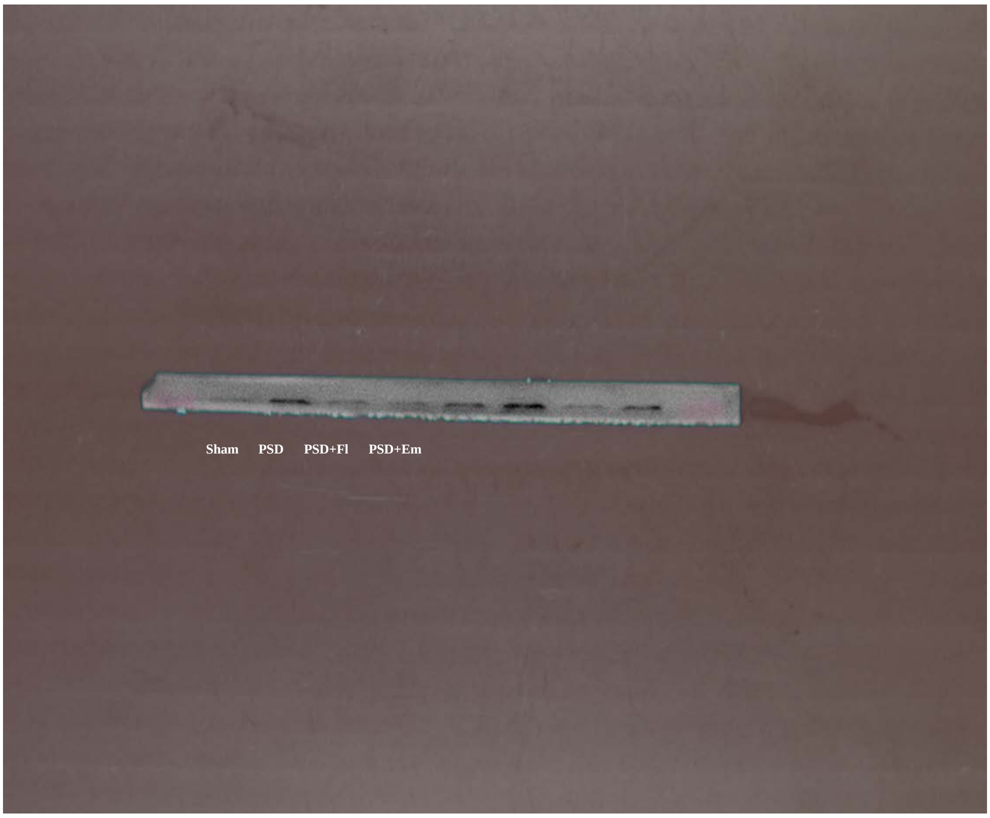

proBDNF-3'

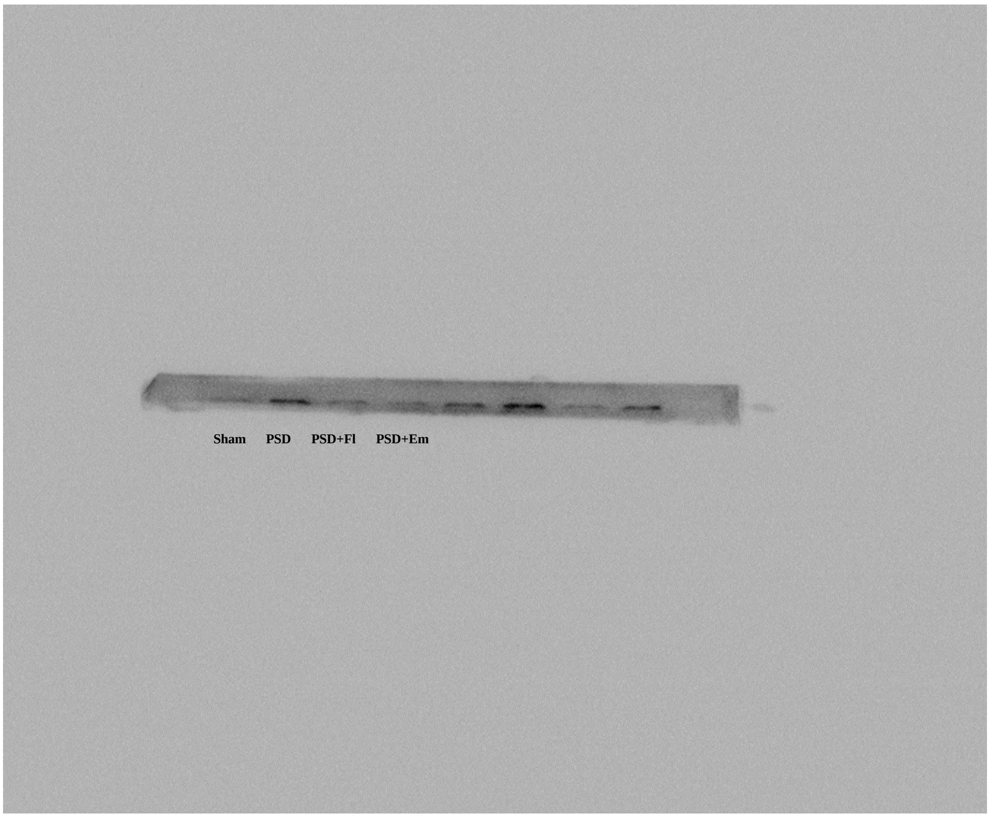

mBDNF-1

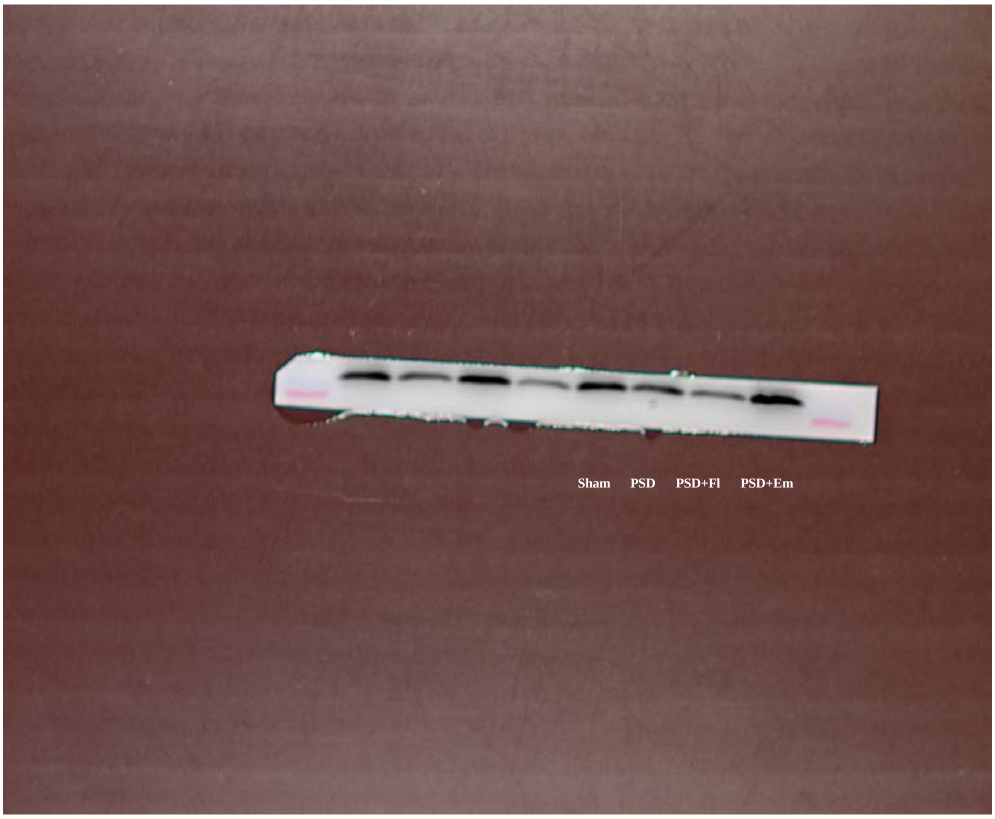

mBDNF-1’

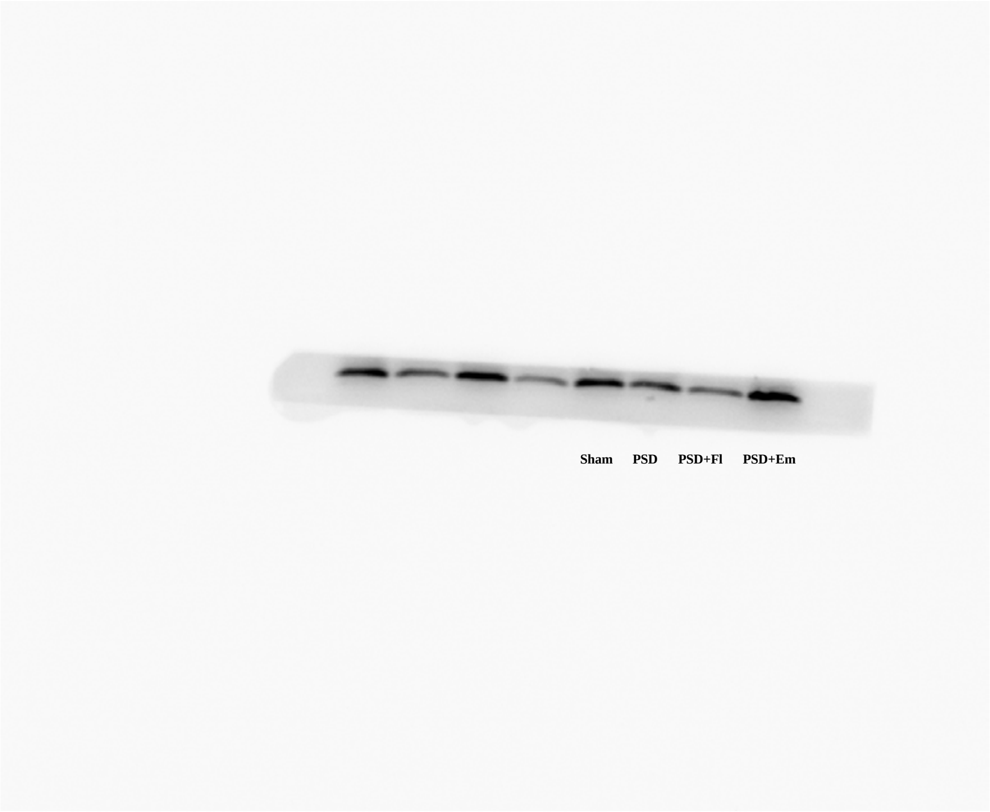

mBDNF-2

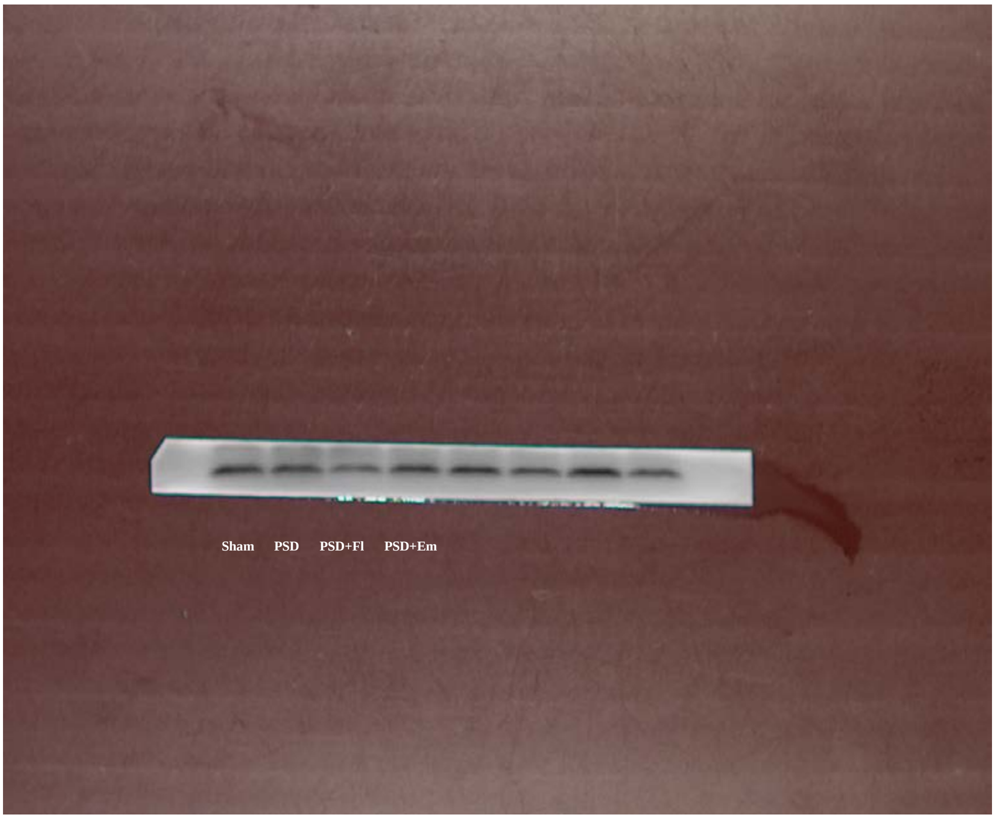

mBDNF-2’

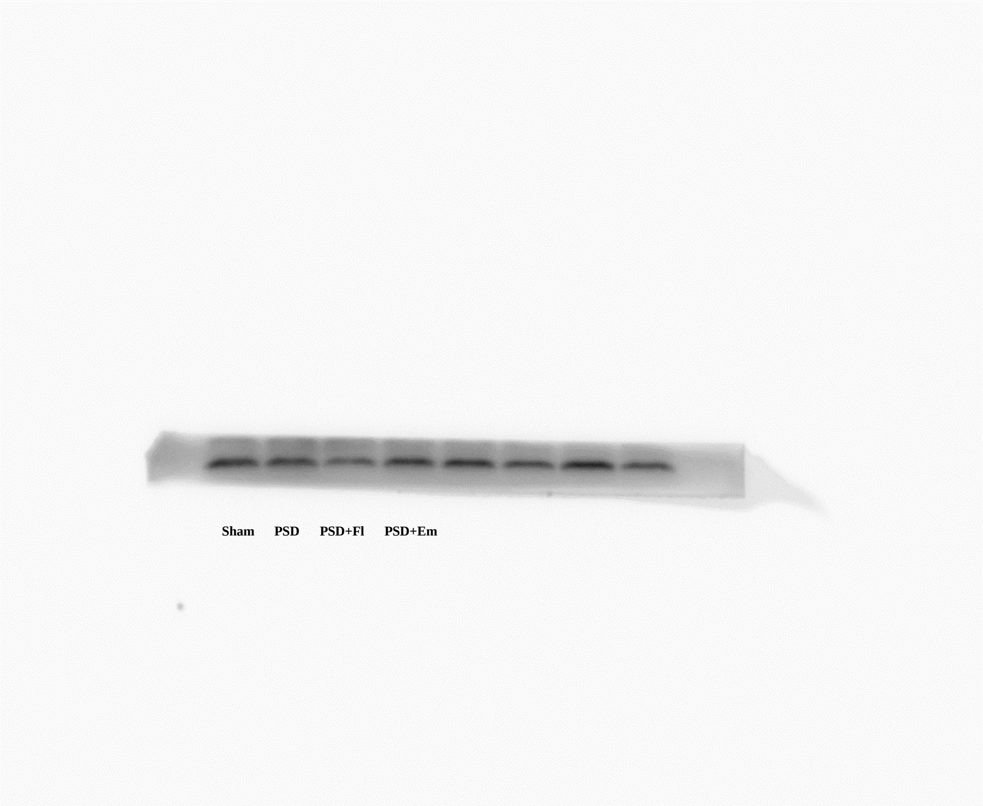

mBDNF-3

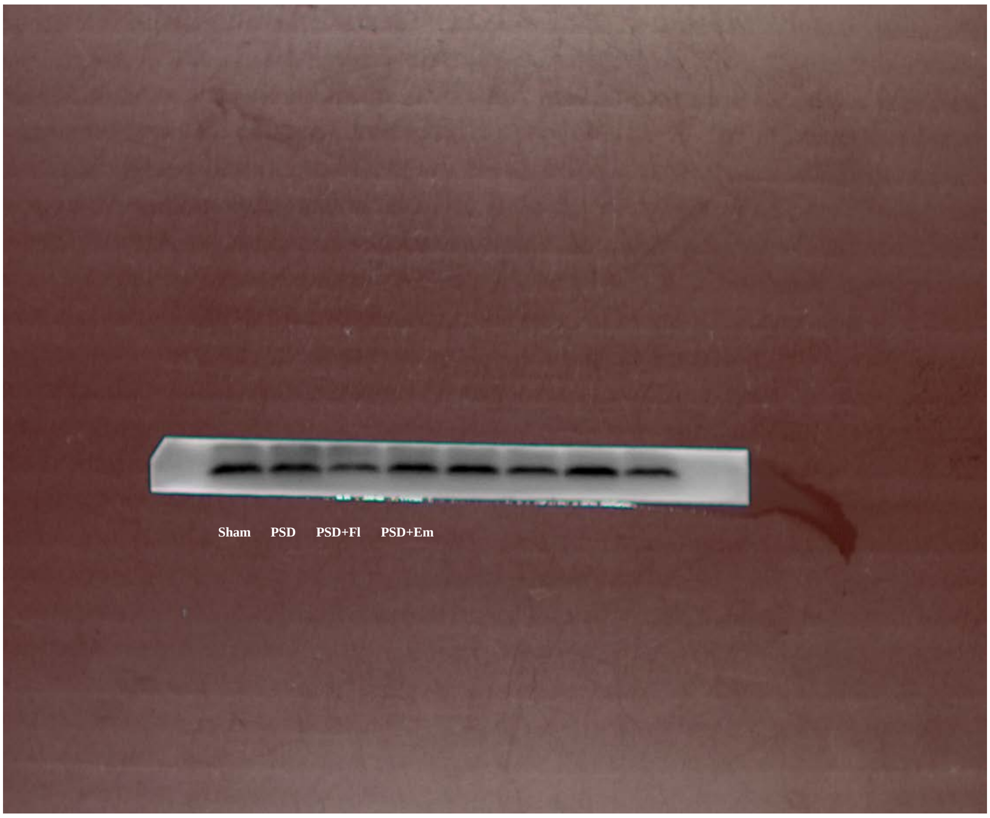

mBDNF-3'

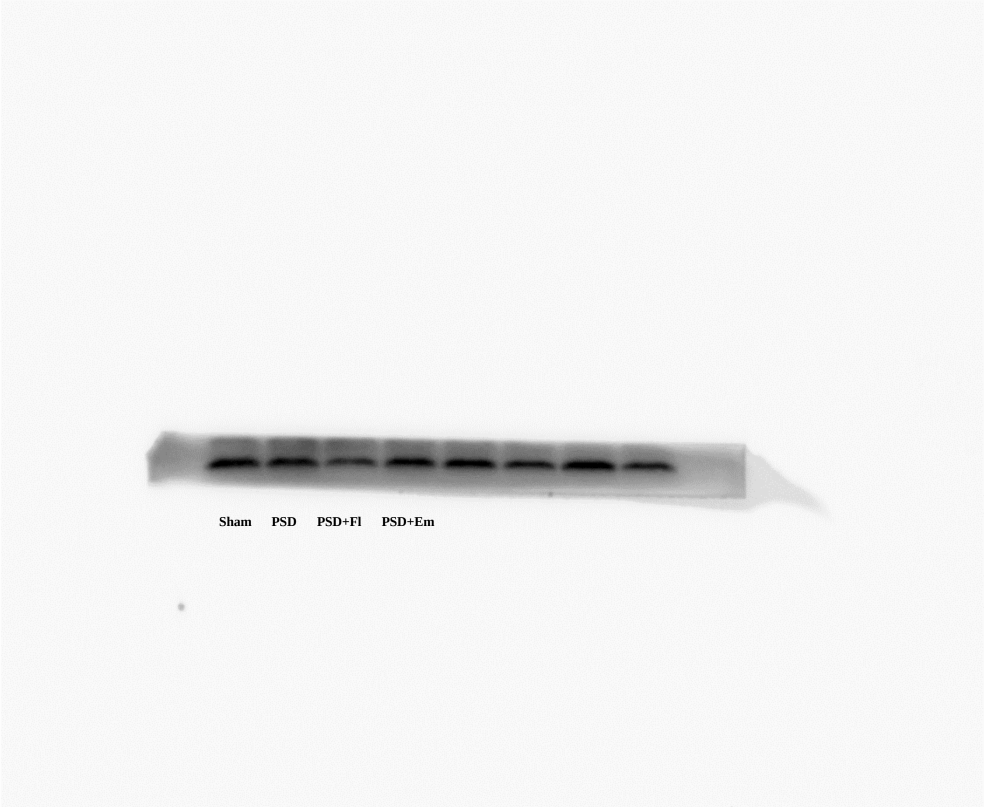

β-actin-1

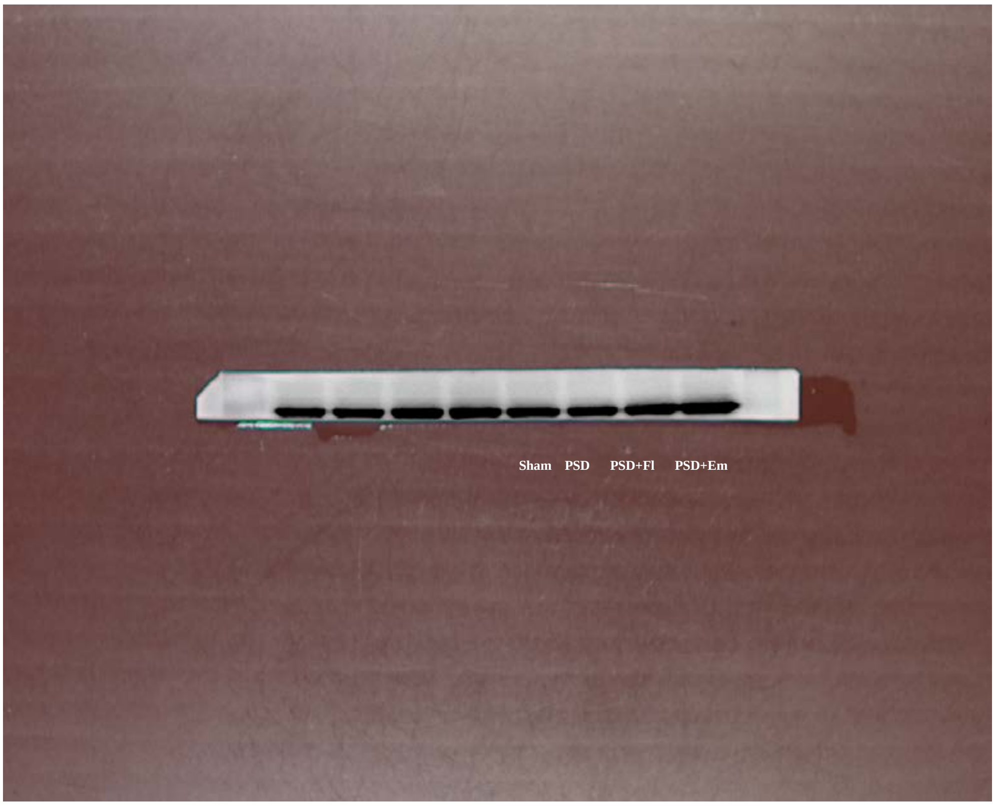

β-actin-1'

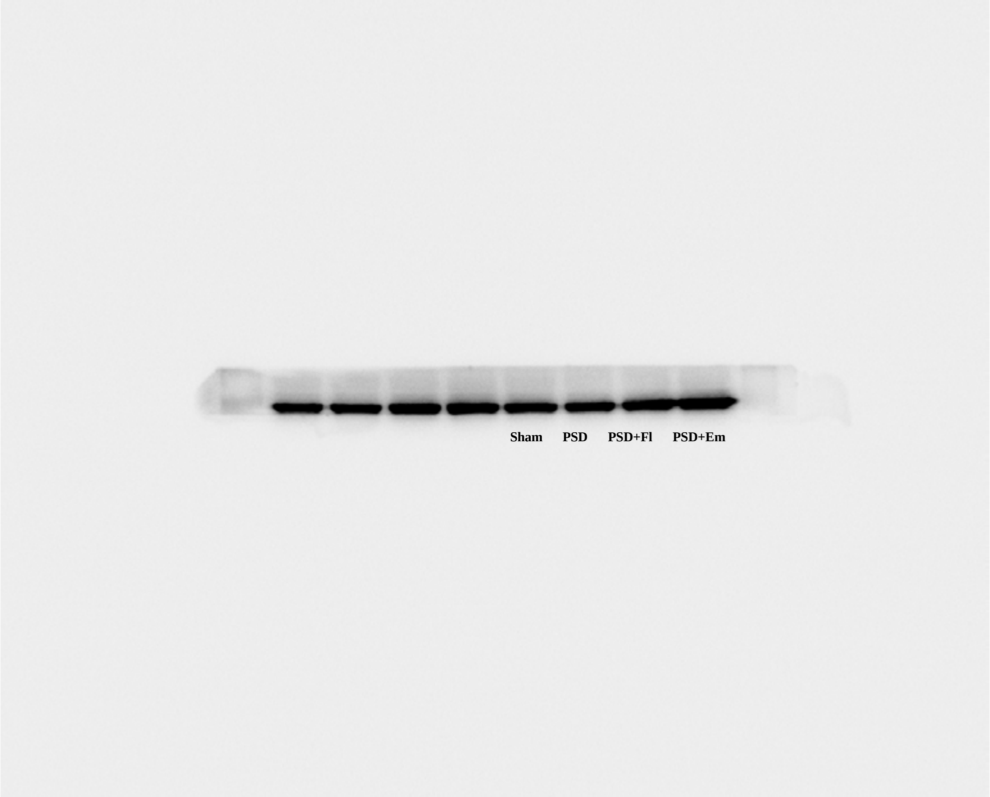

β-actin-2

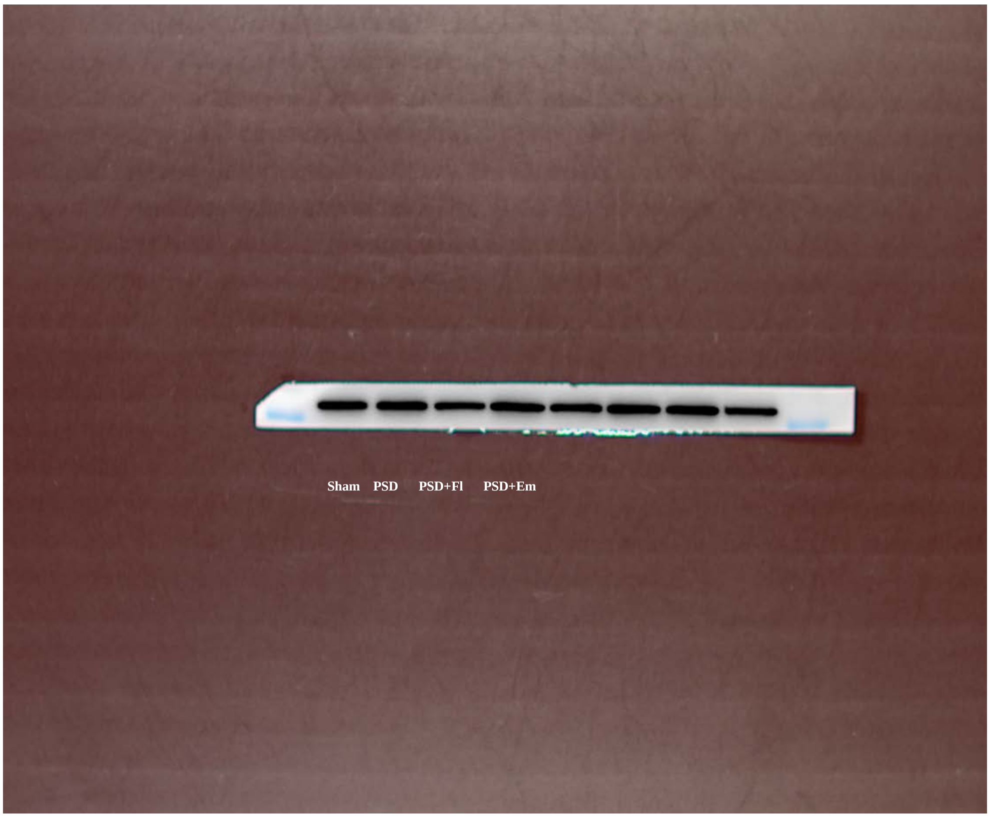

β-actin-2’

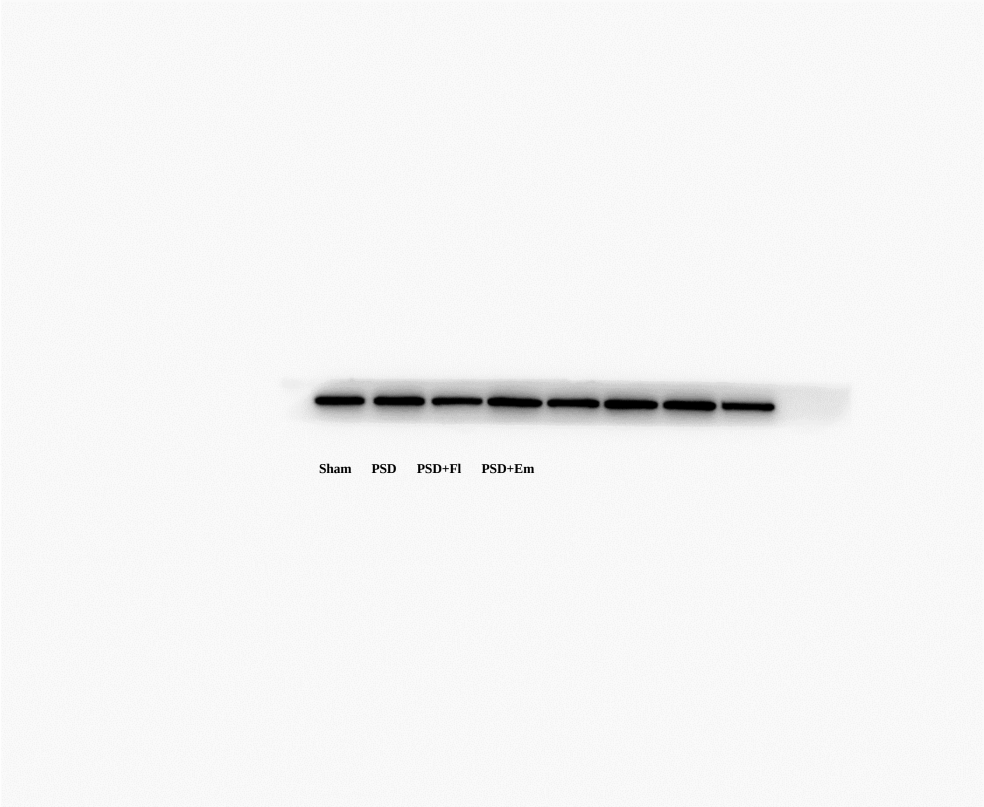

β-actin-3

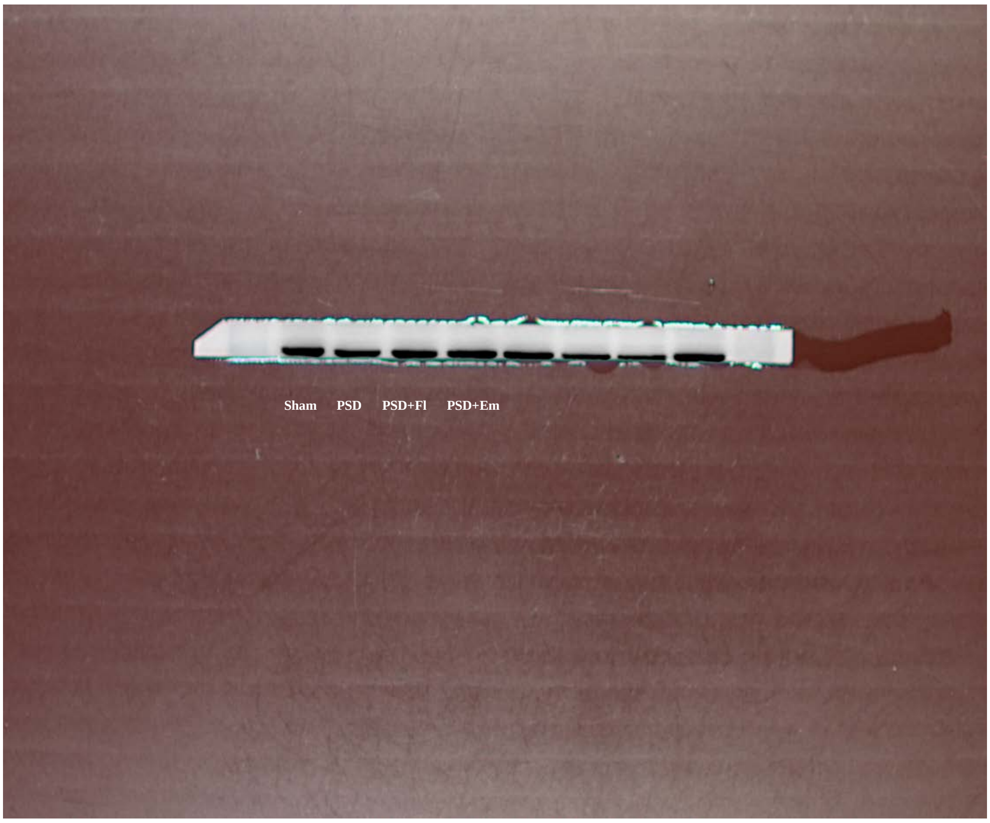

β-actin-3’

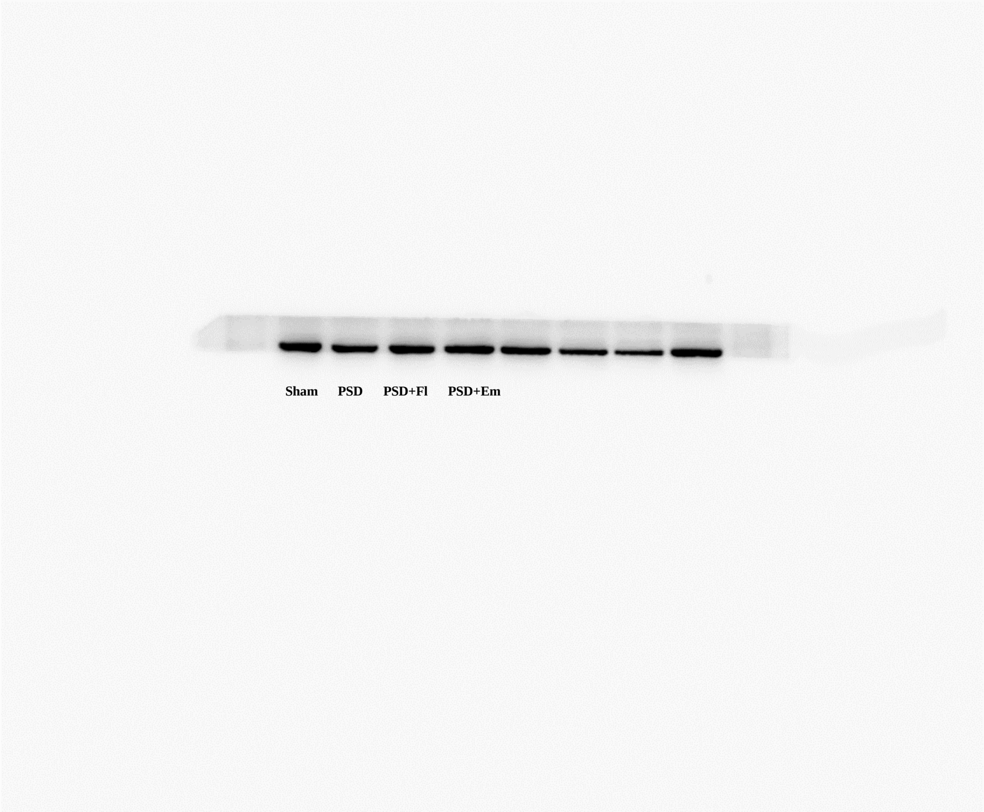

Figure.9 D proBDNF, mBDNF proteins in the mPFC

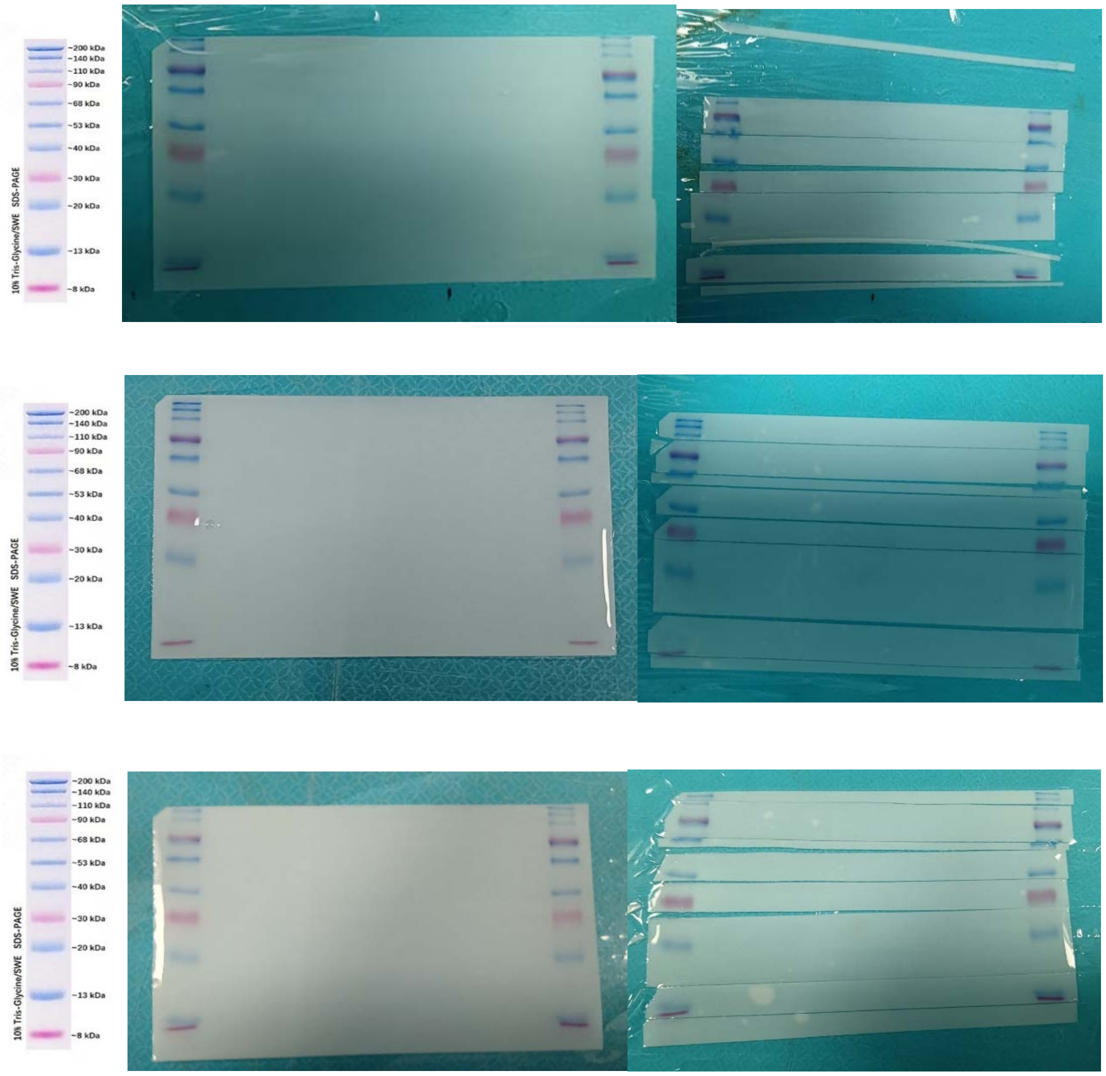

Figure 9D Complete unedited gel/imprint

proBDNF-1

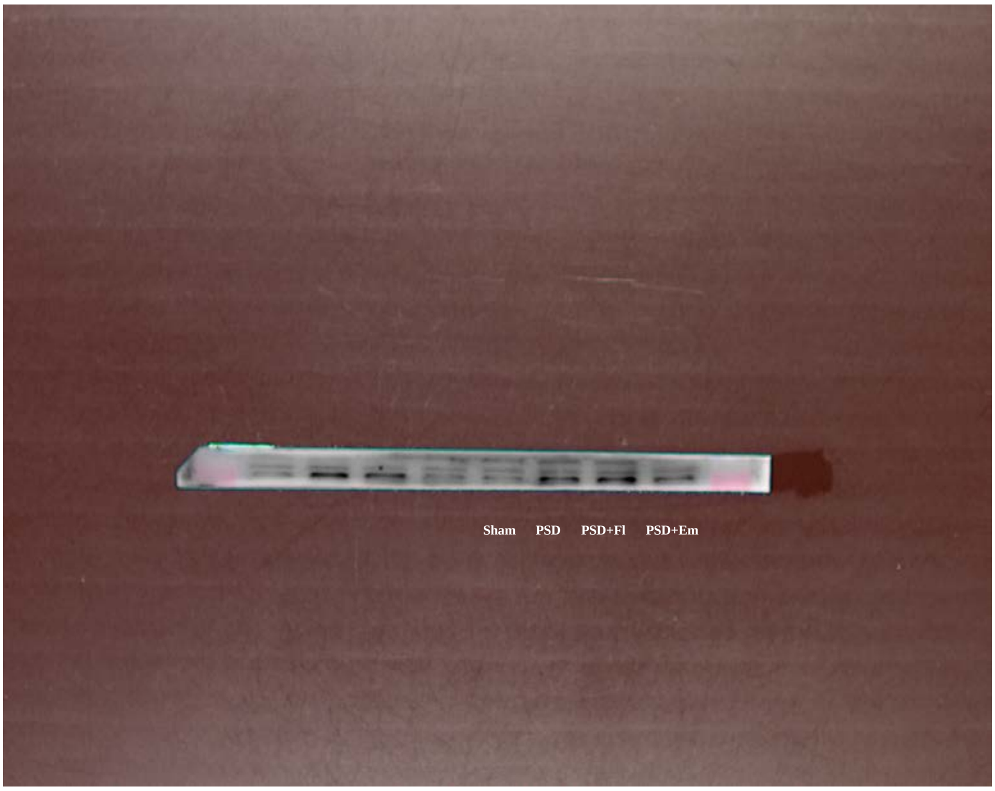

proBDNF-1’

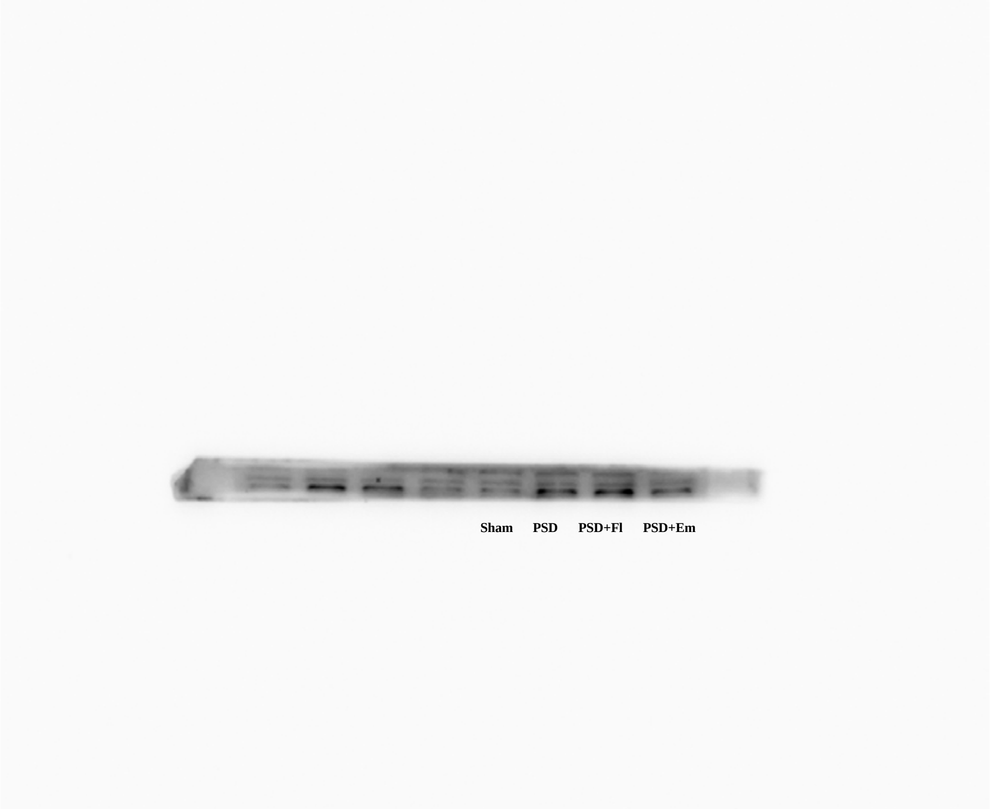

proBDNF-2

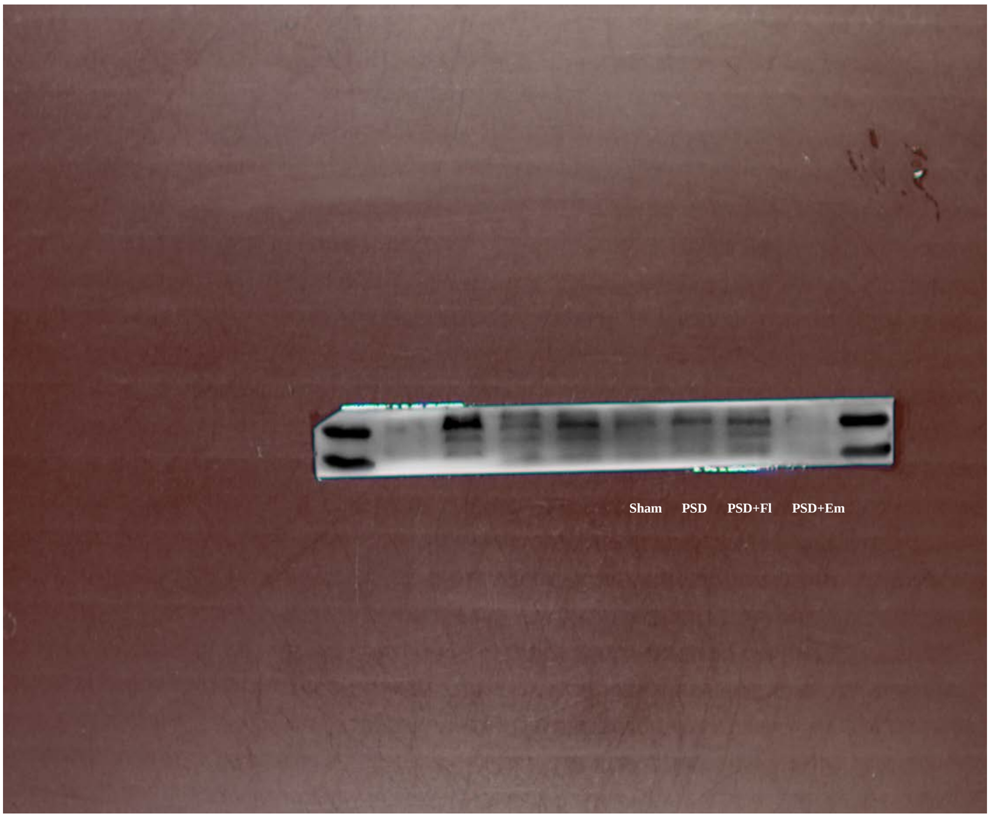

proBDNF-2'

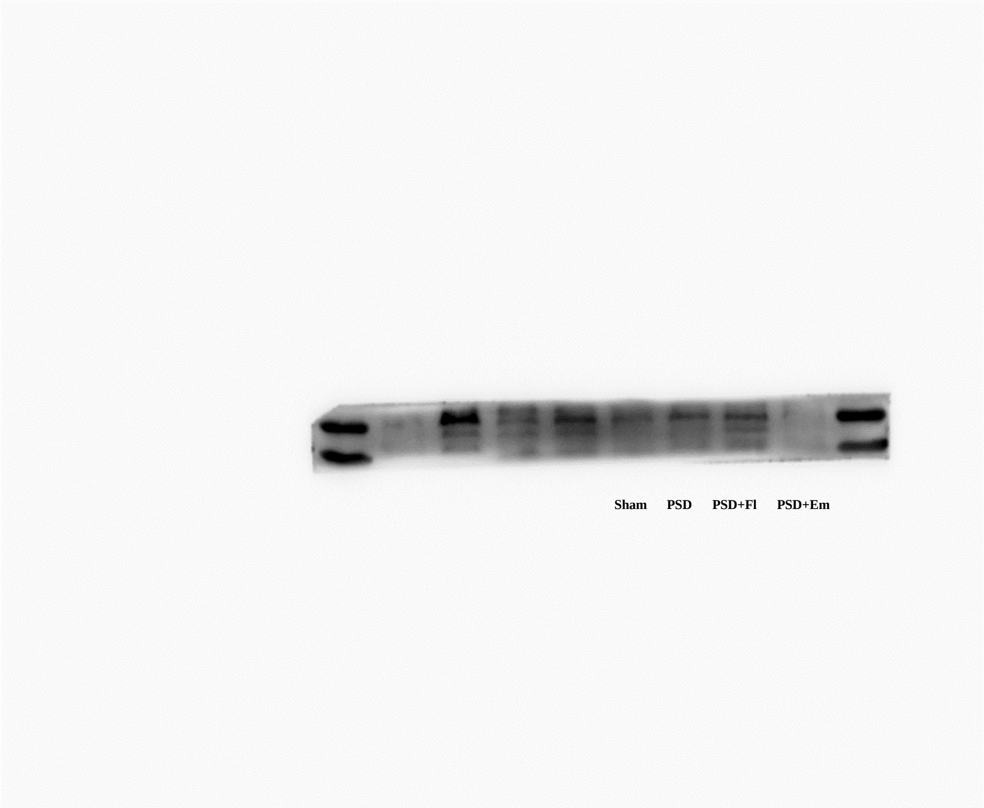

proBDNF-3

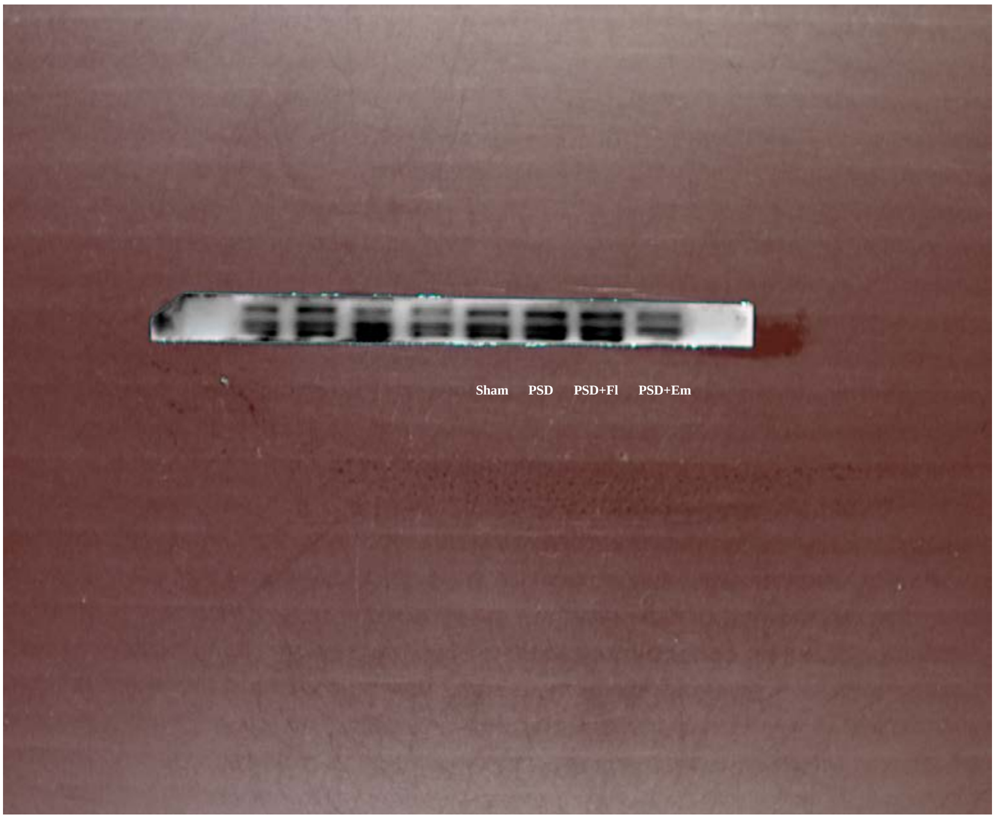

proBDNF-3’

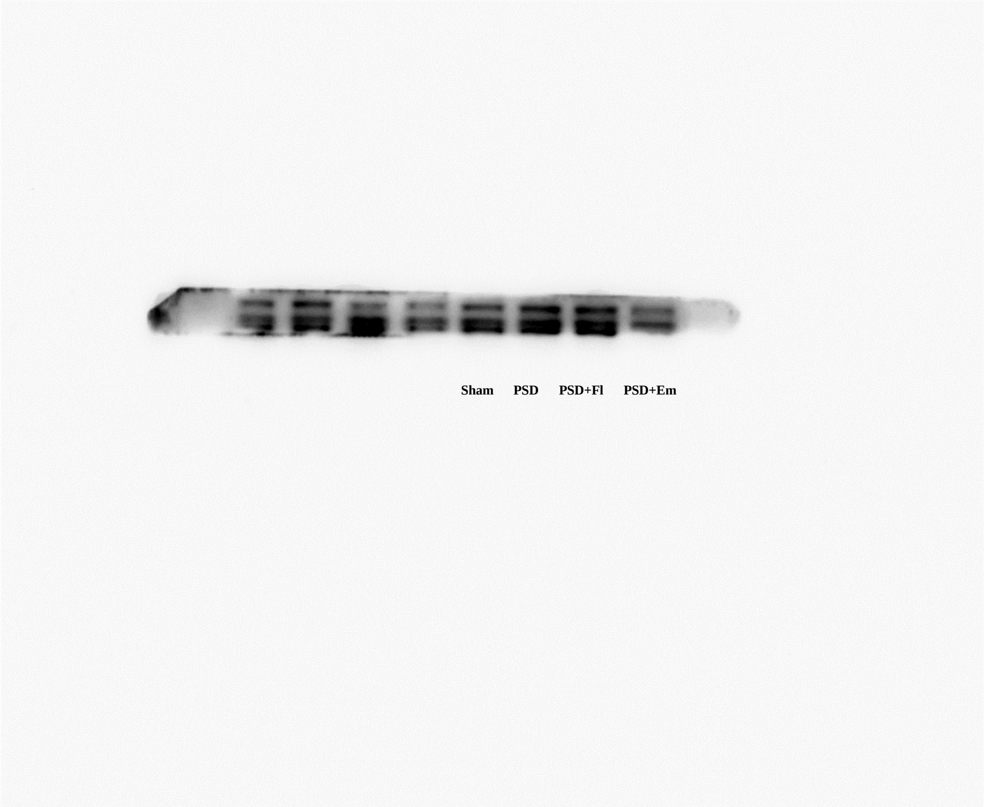

mBDNF-1

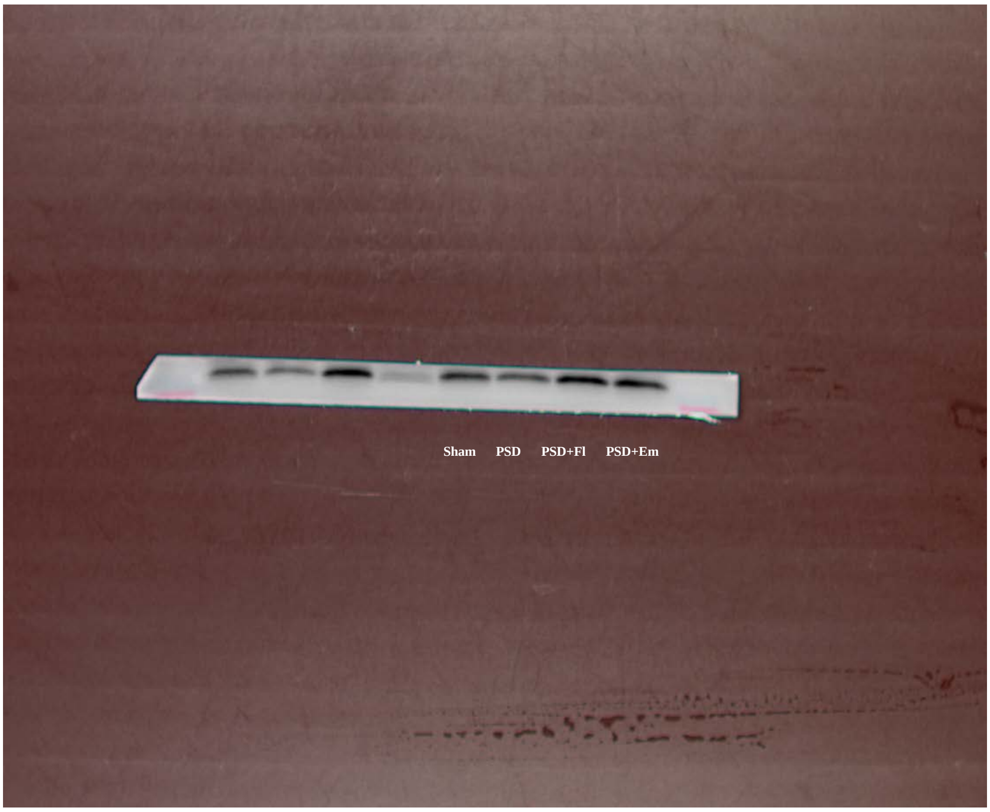

mBDNF-1'

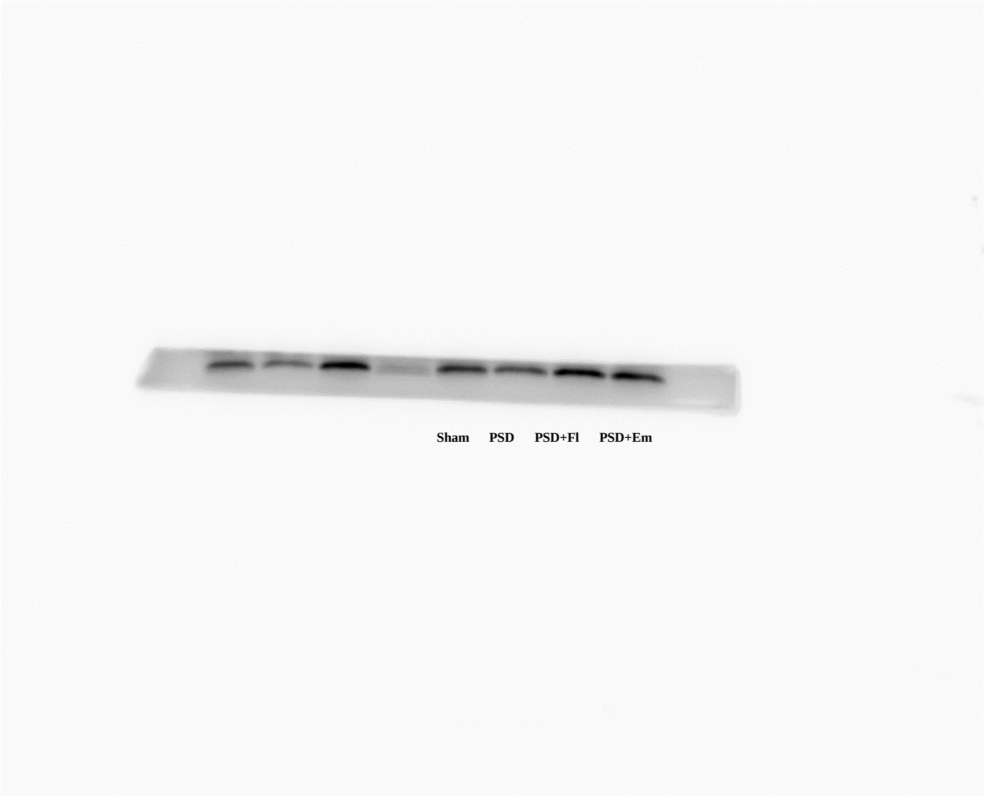

mBDNF-2

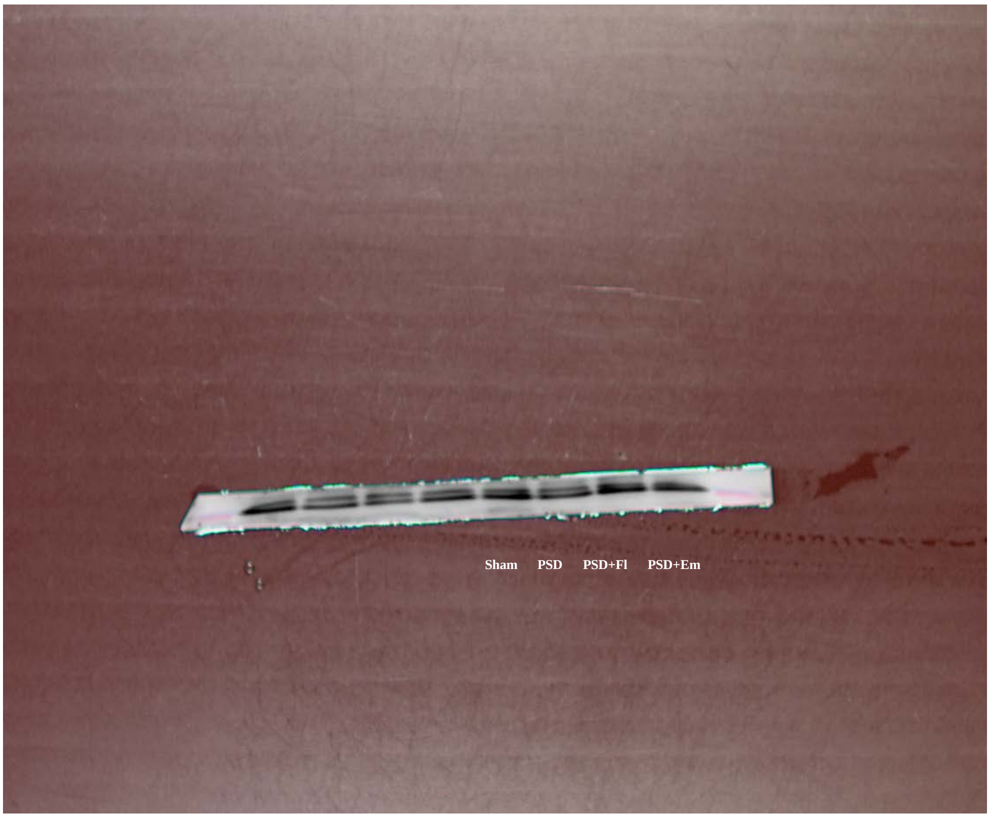

mBDNF-2’

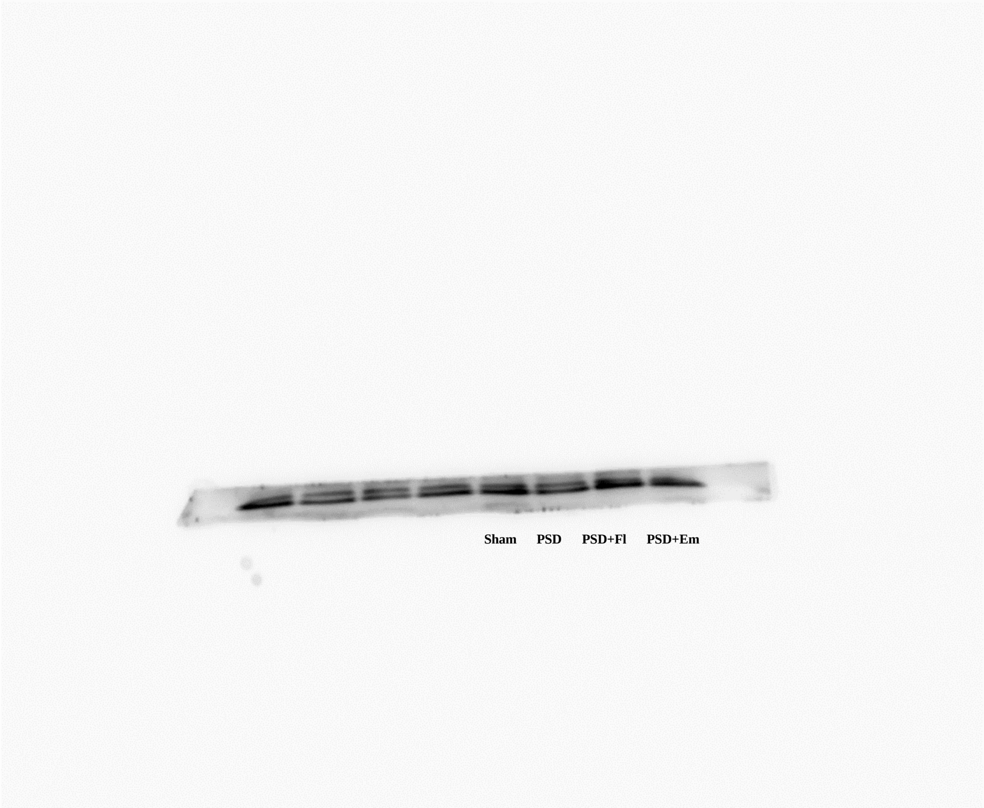

mBDNF-3

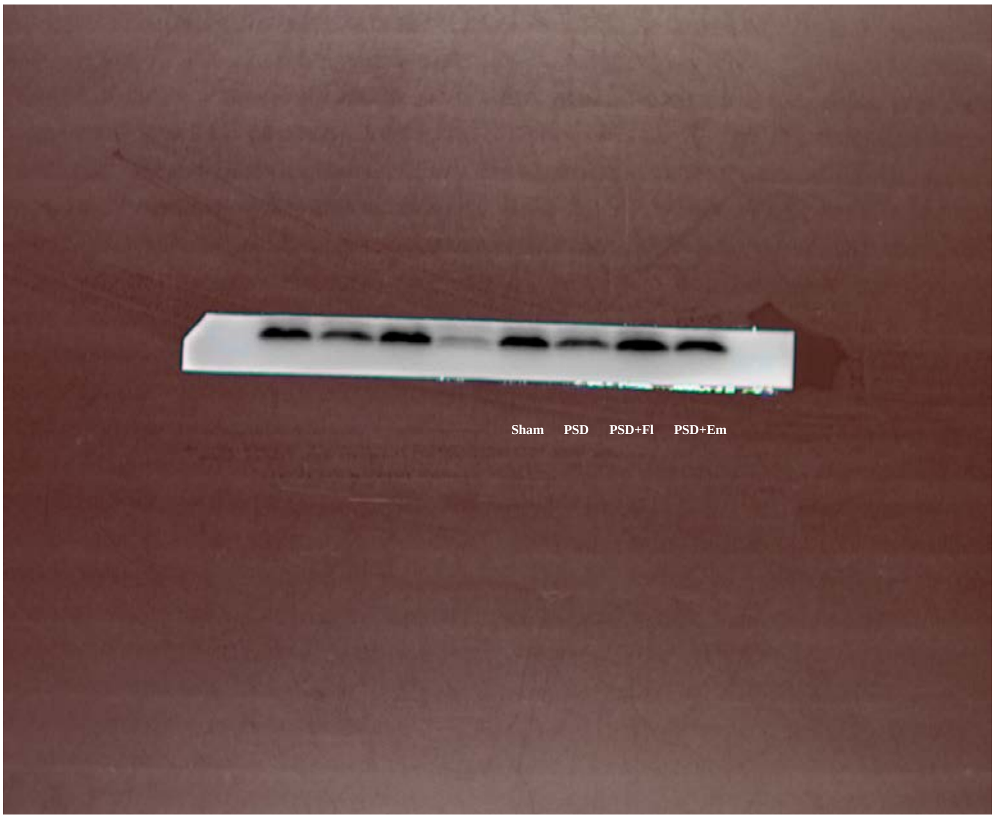

mBDNF-3’

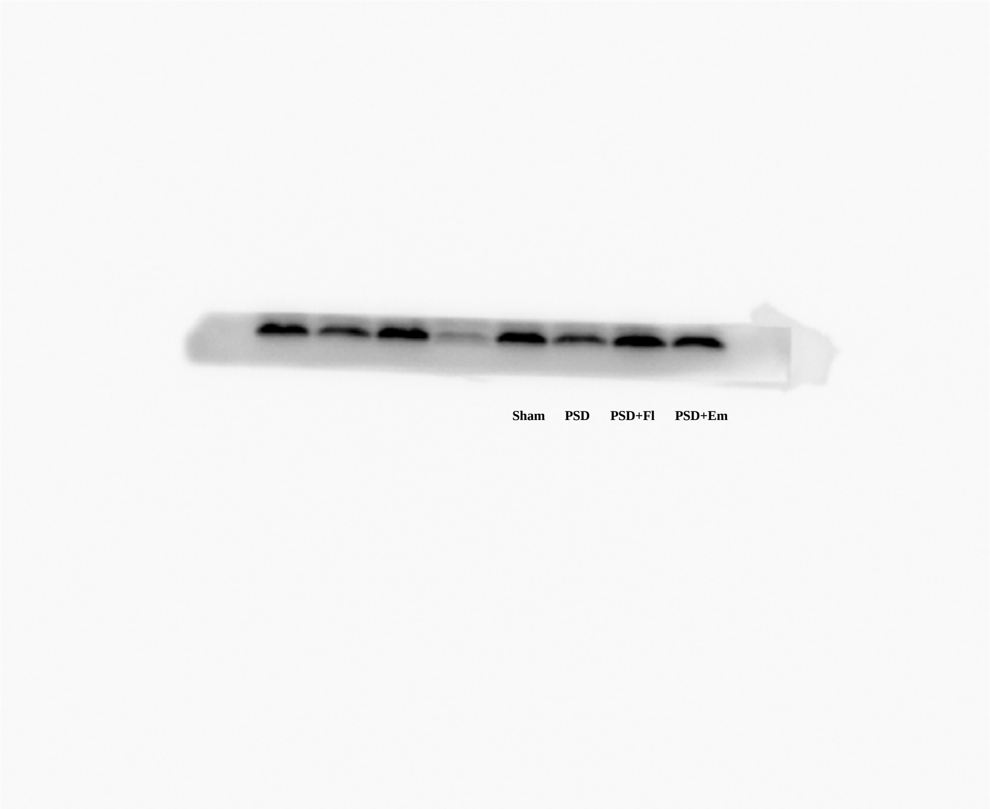

β-actin-1

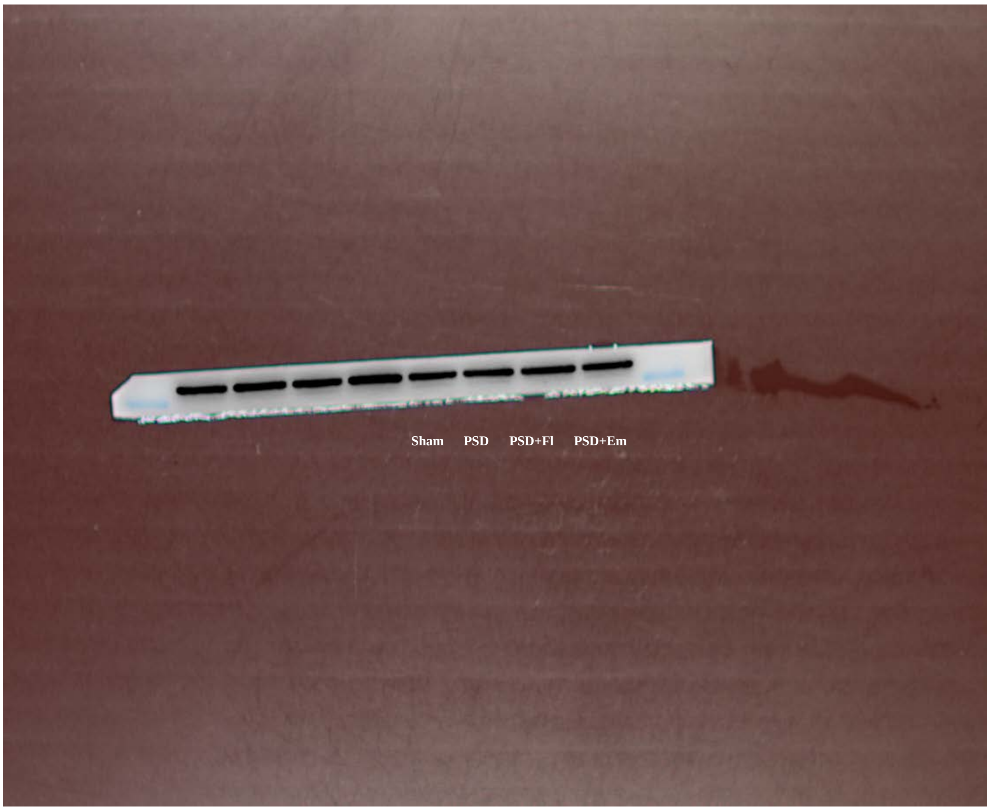

β-actin-1’

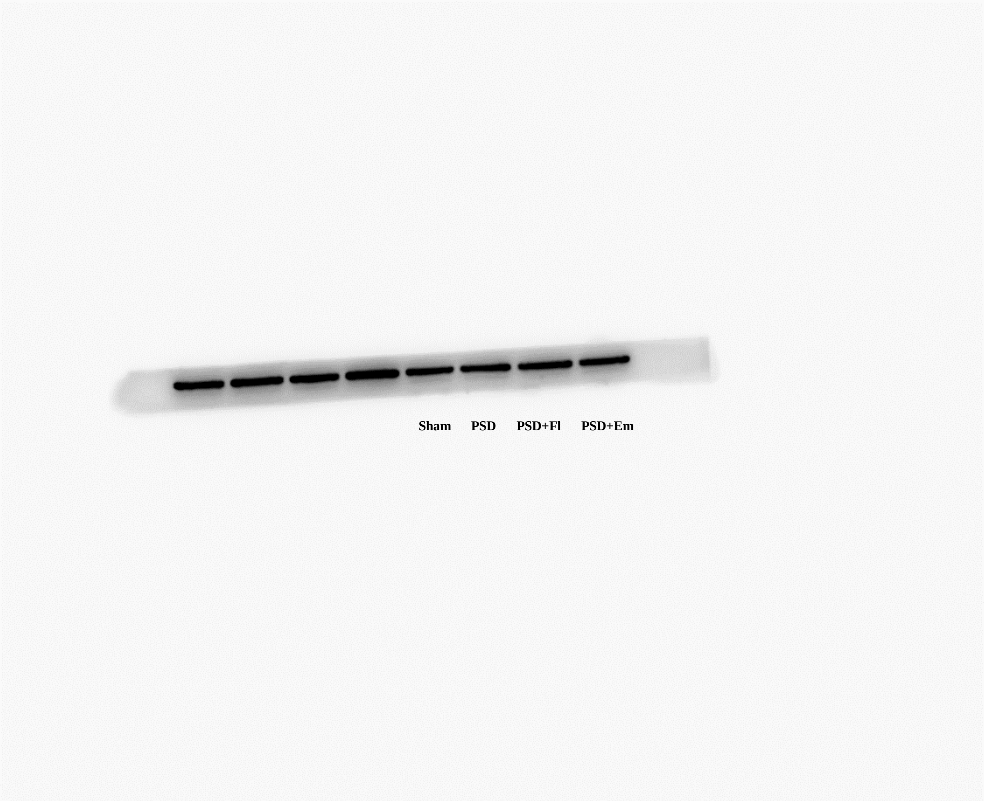

β-actin-2

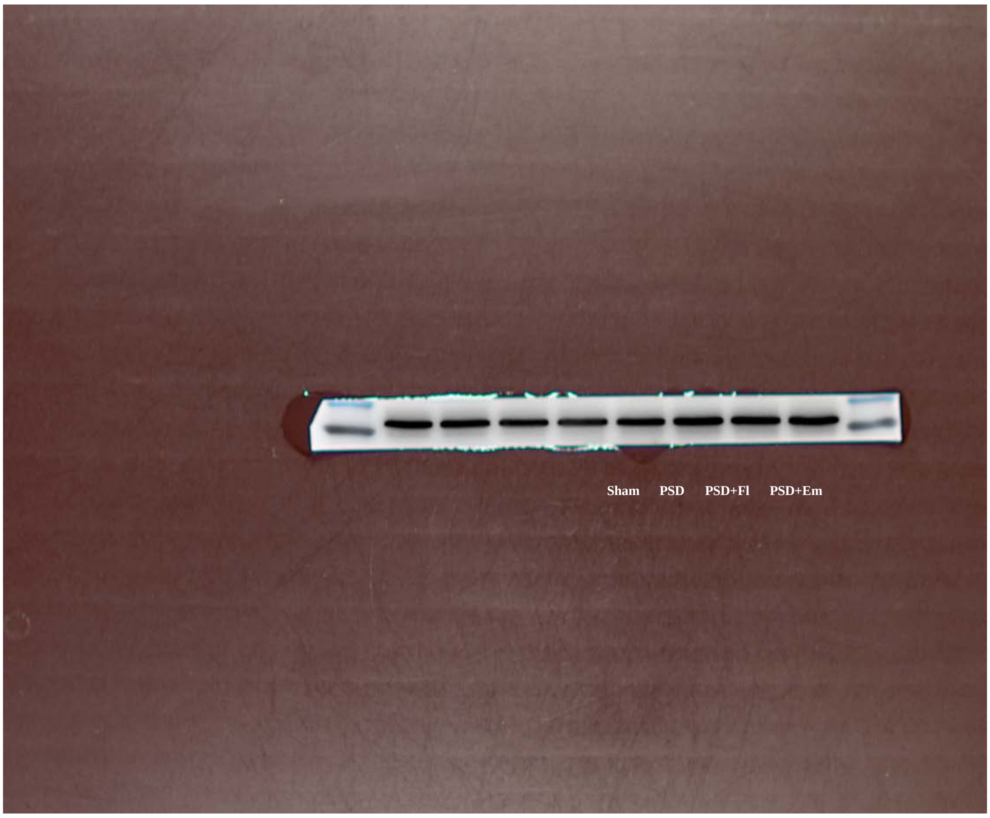

β-actin-2’

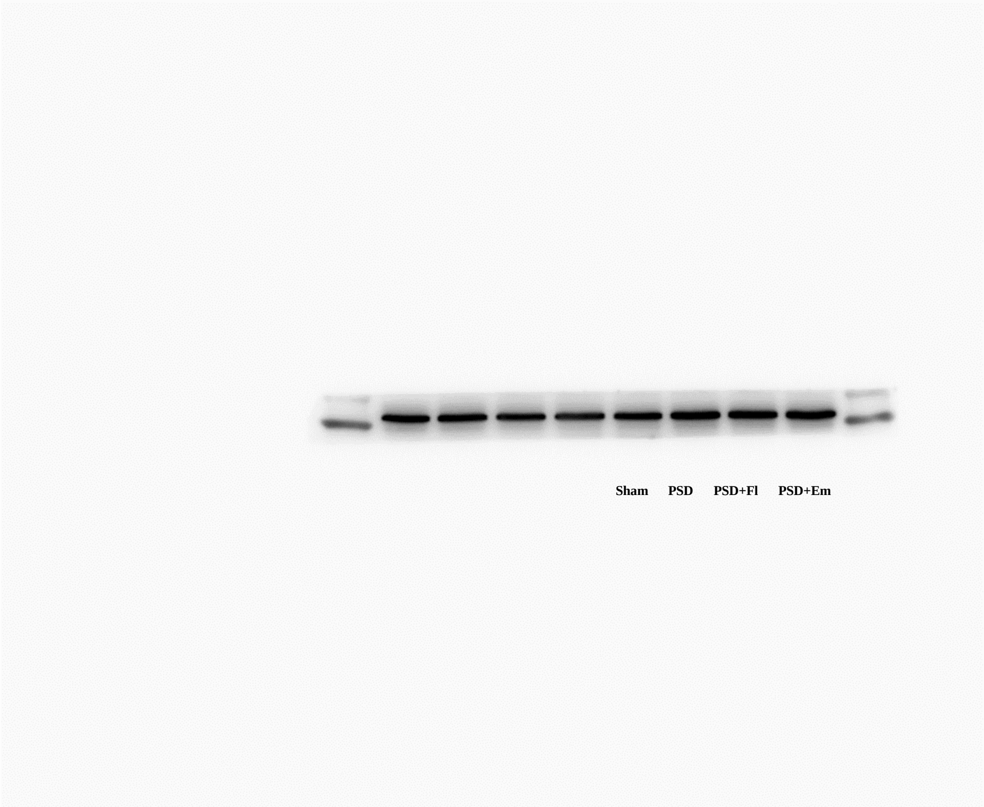

β-actin-3

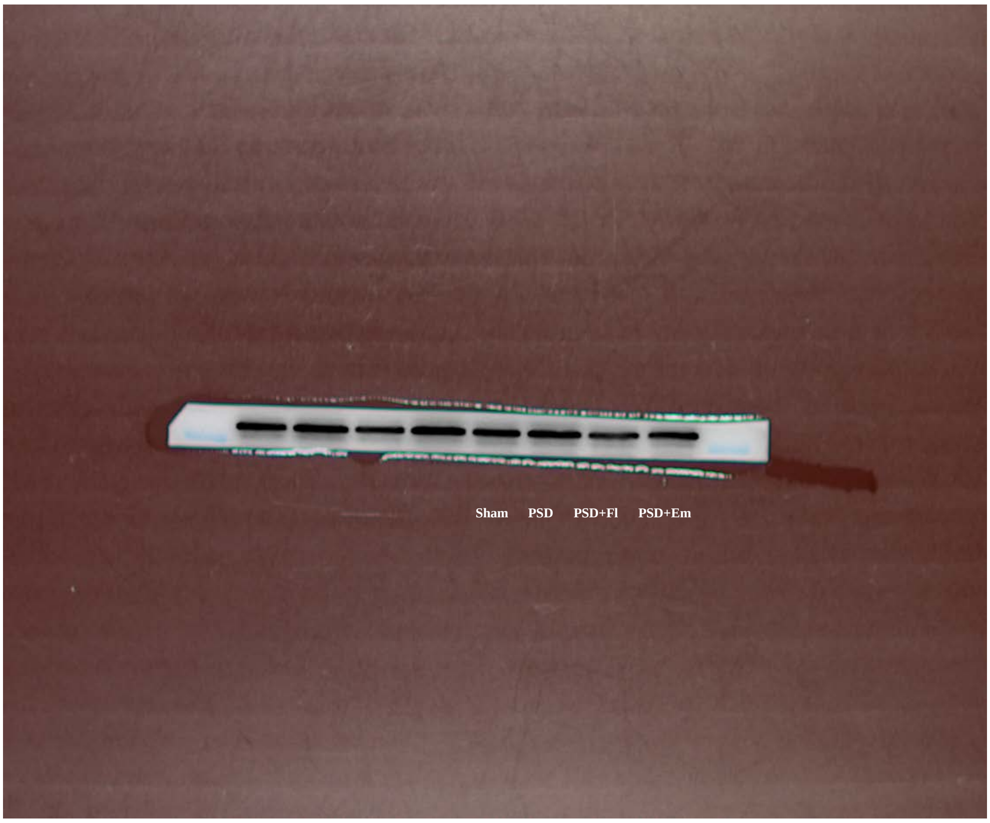

β-actin-3’

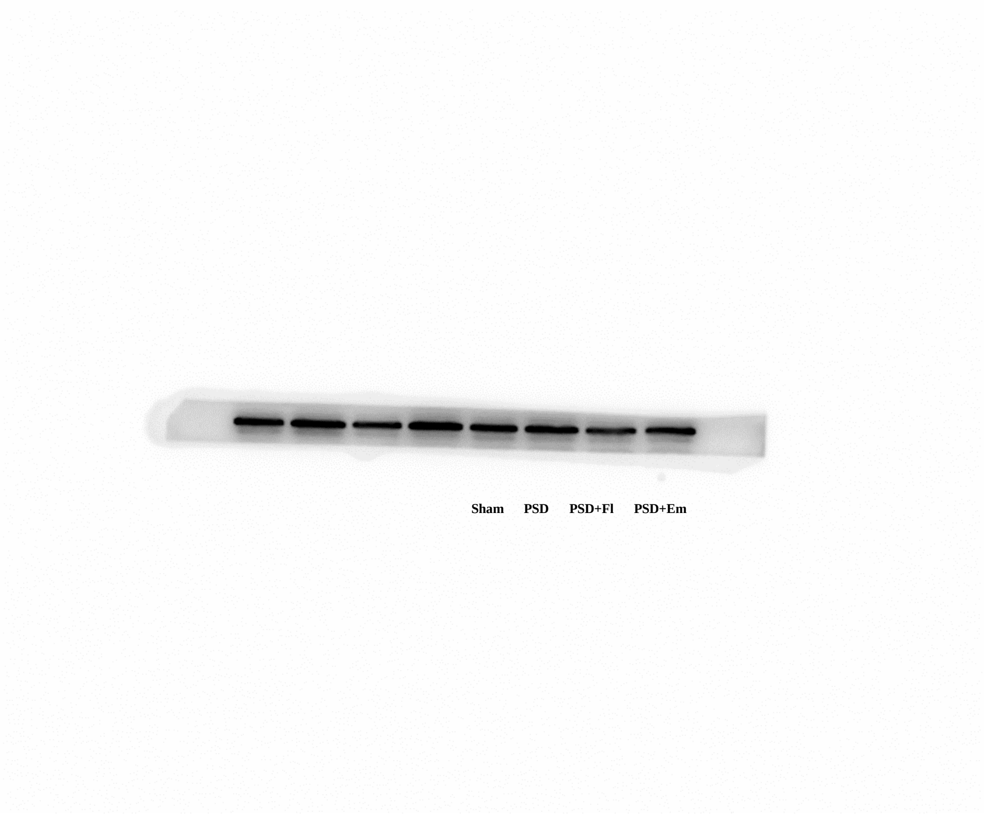

Figure. 10 E **tPA, MMP 9, Furin, and PC proteins in the Hippocampus**

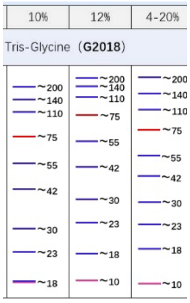

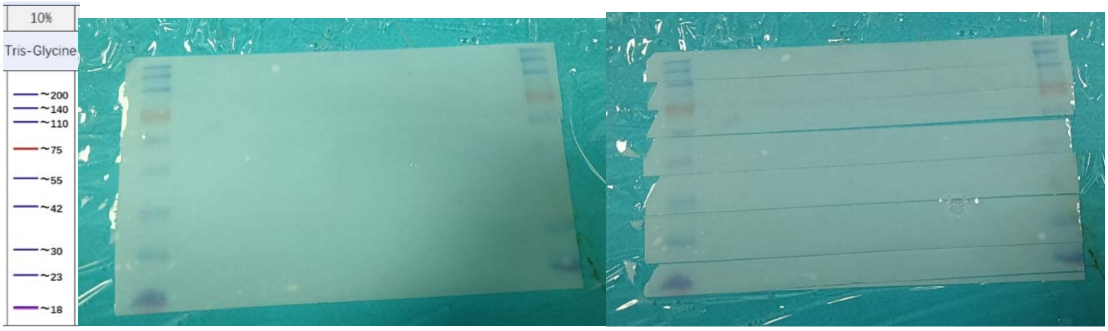

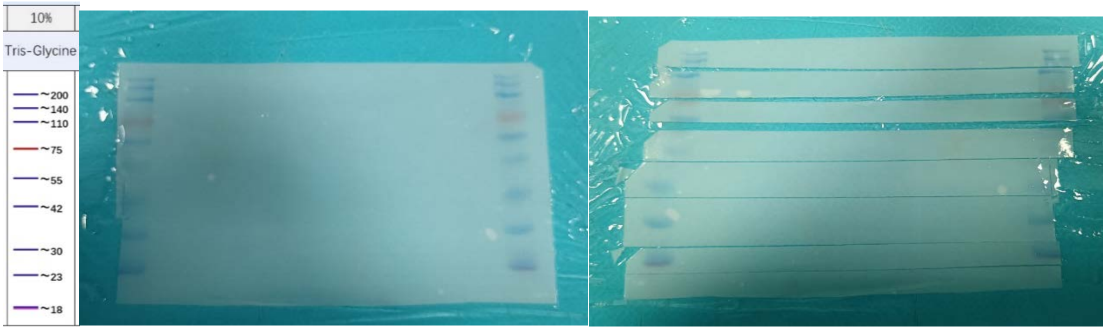

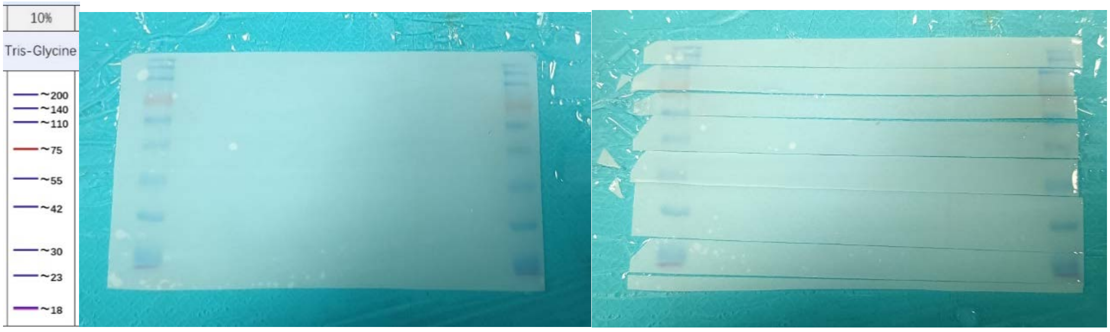

Figure 10E Complete unedited gel/imprint

tPA-1

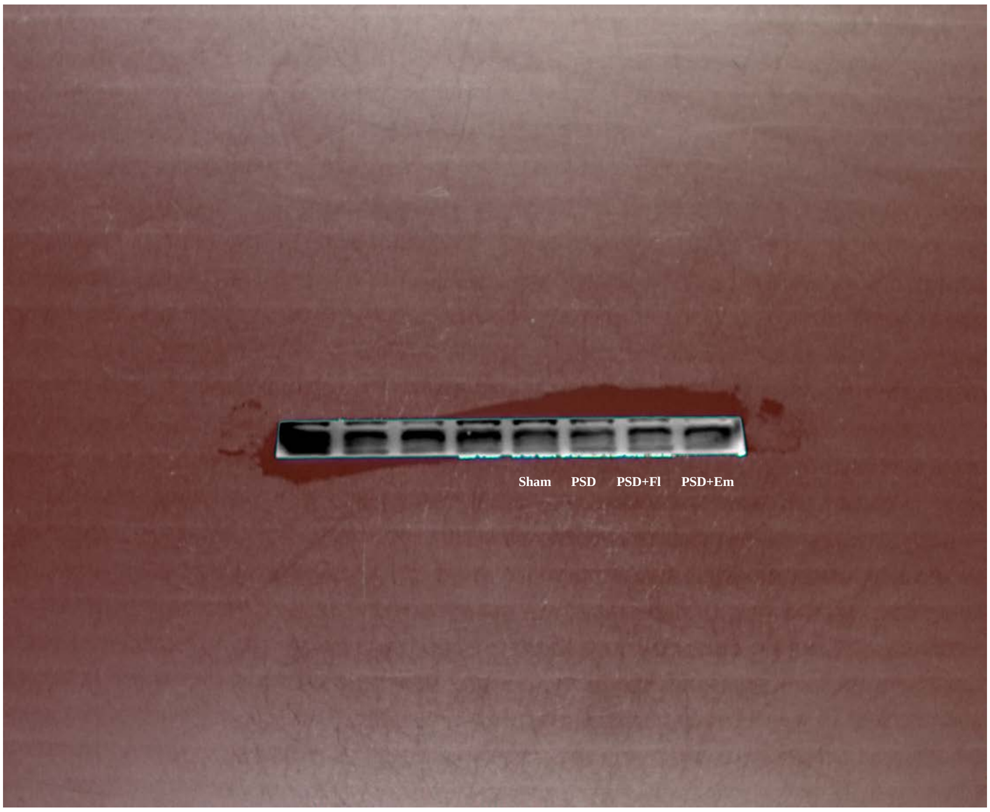

tPA-1’

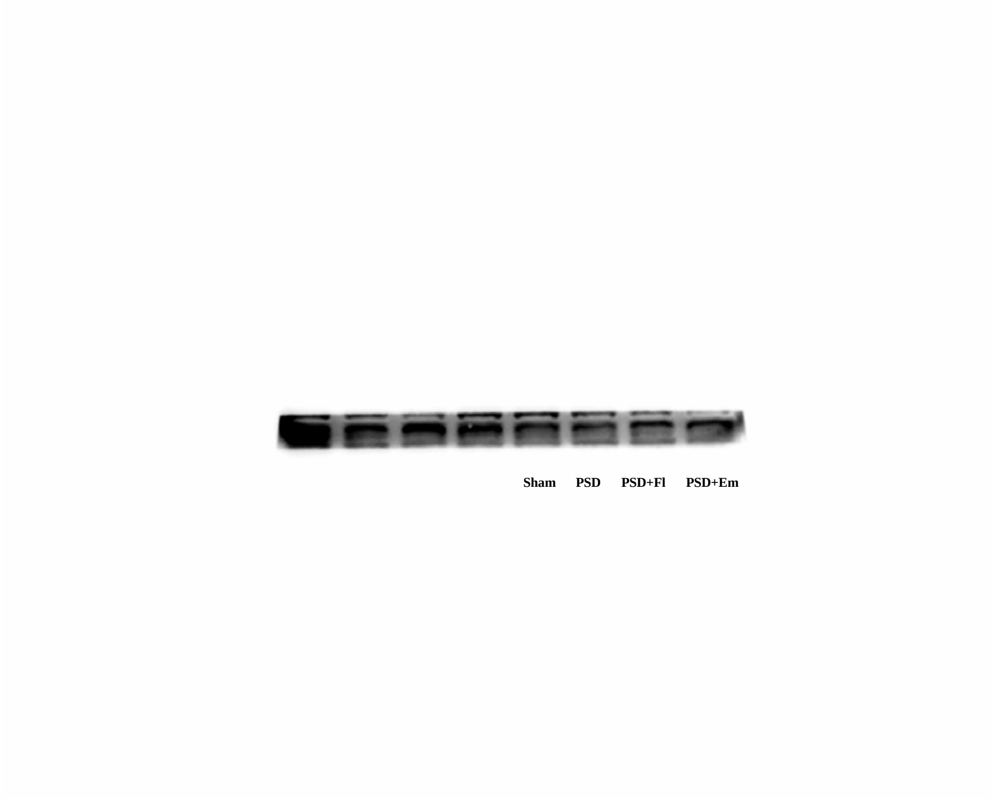

tPA-2

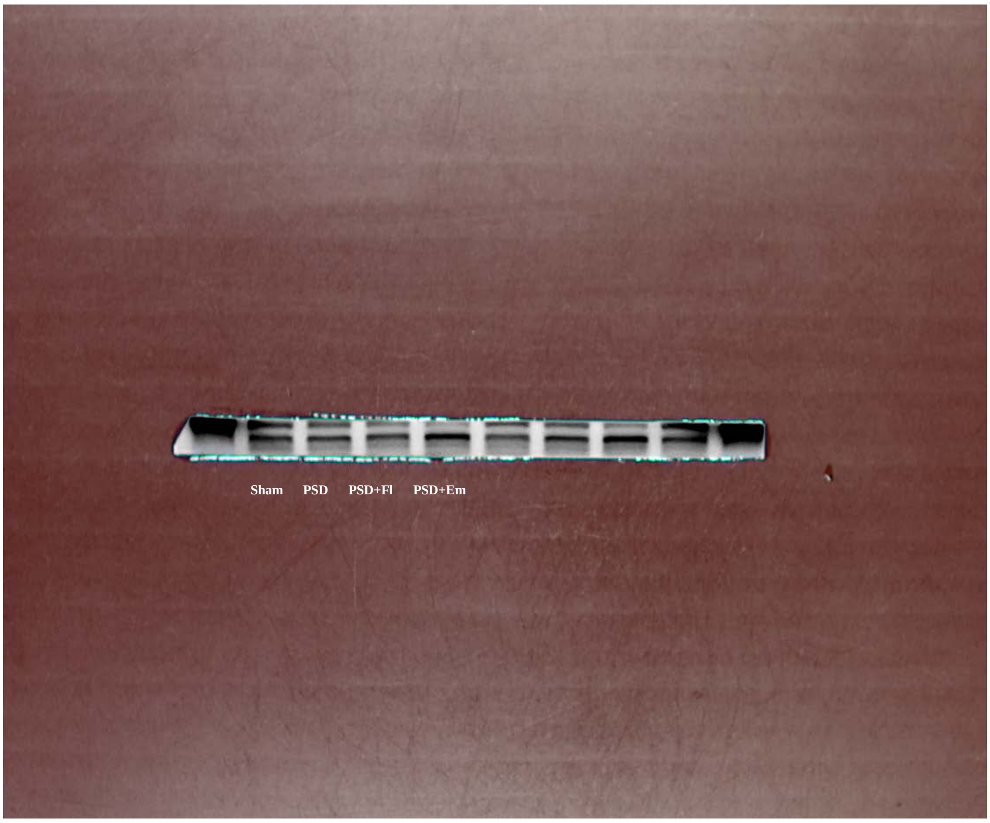

tPA-2’

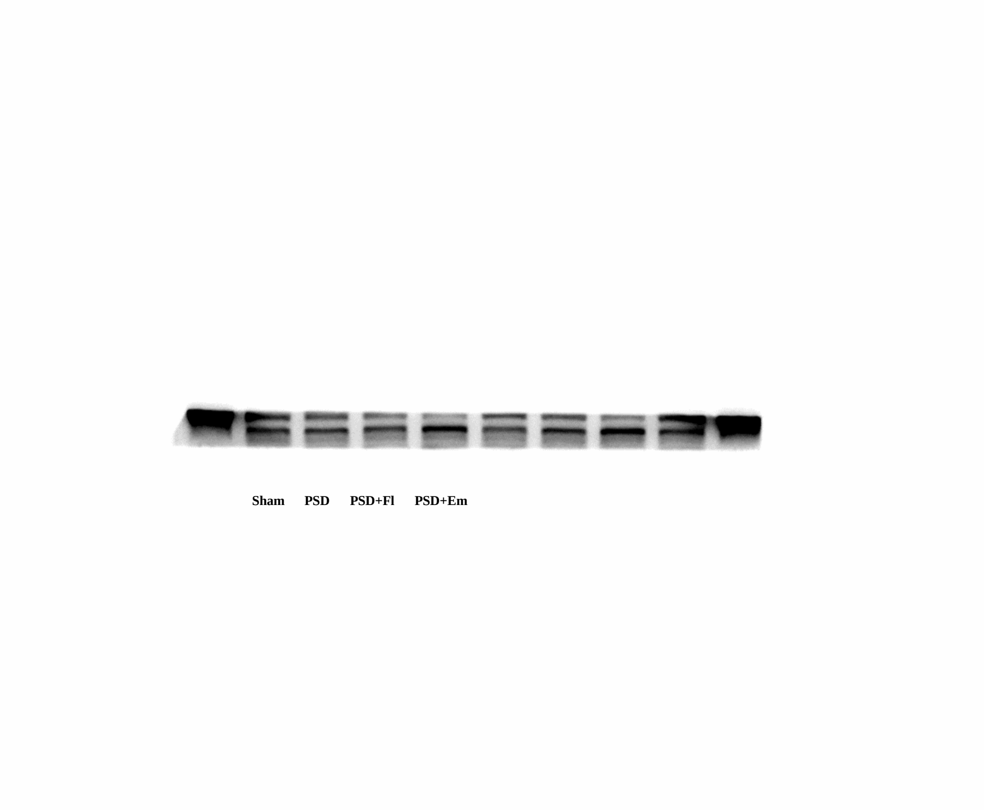

tPA-3

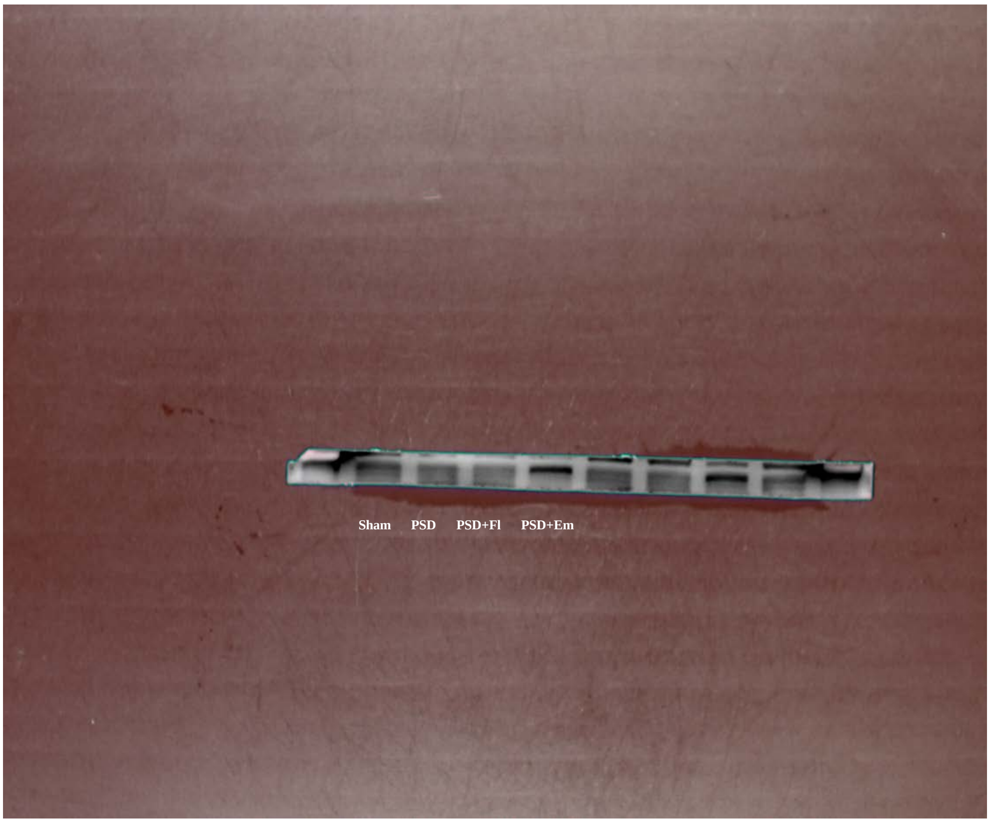

tPA-3′

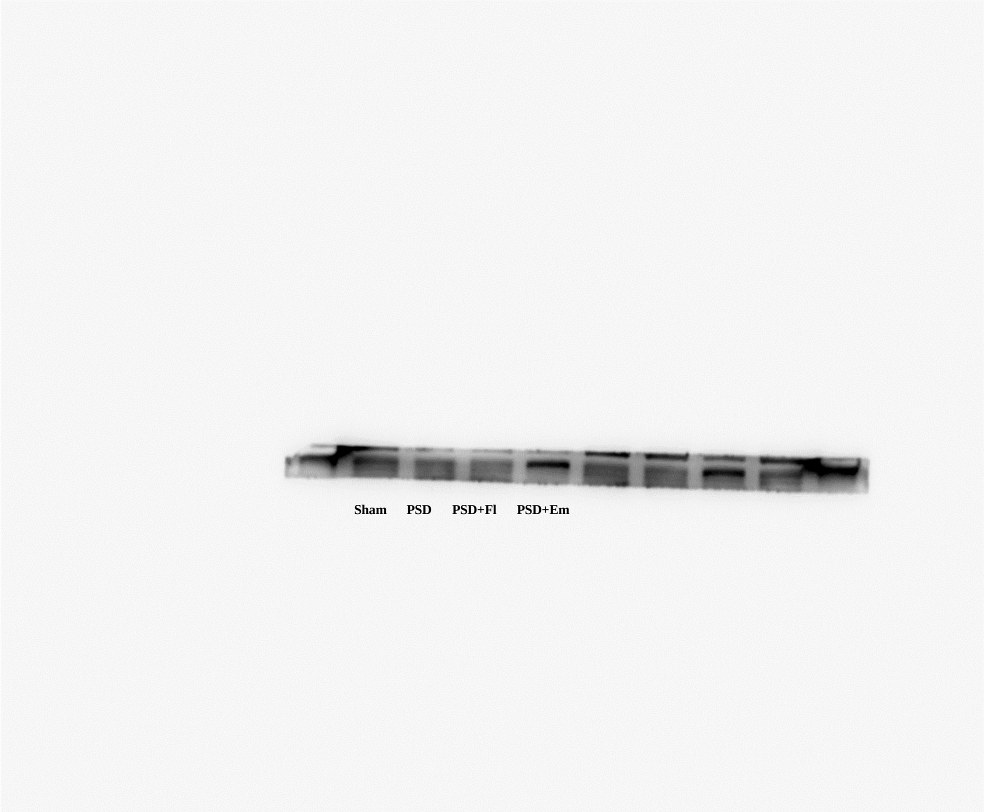

Furin-1

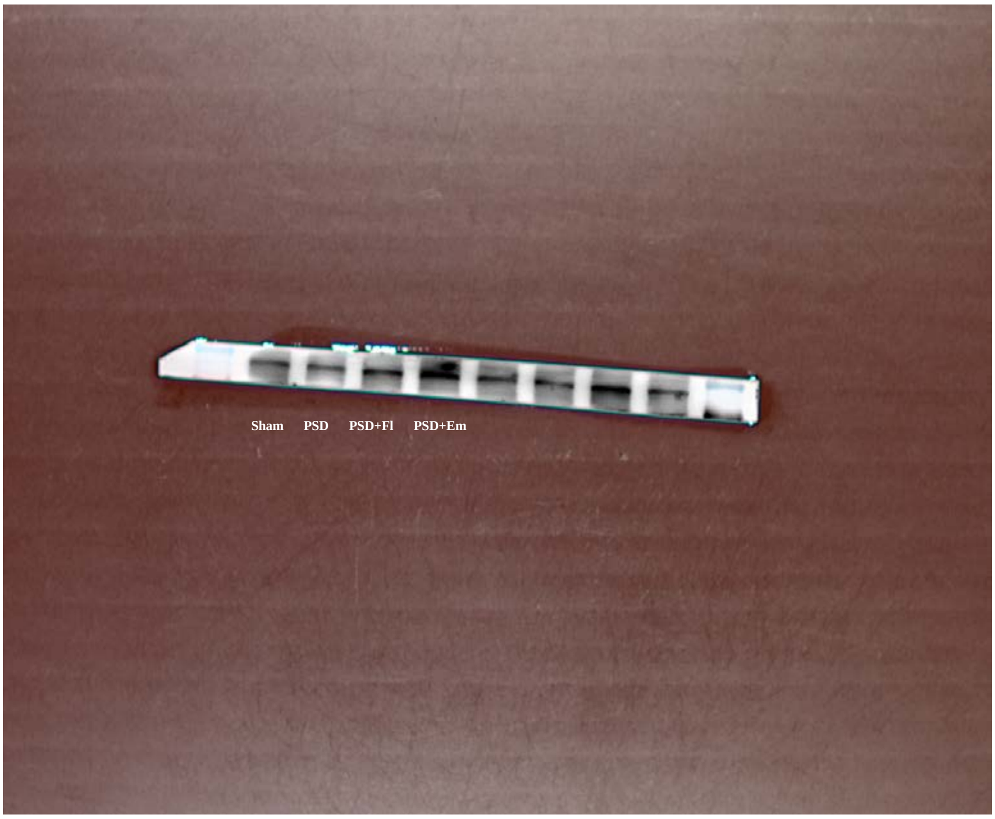

Furin-1′

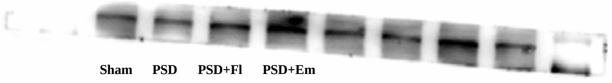

Furin-2

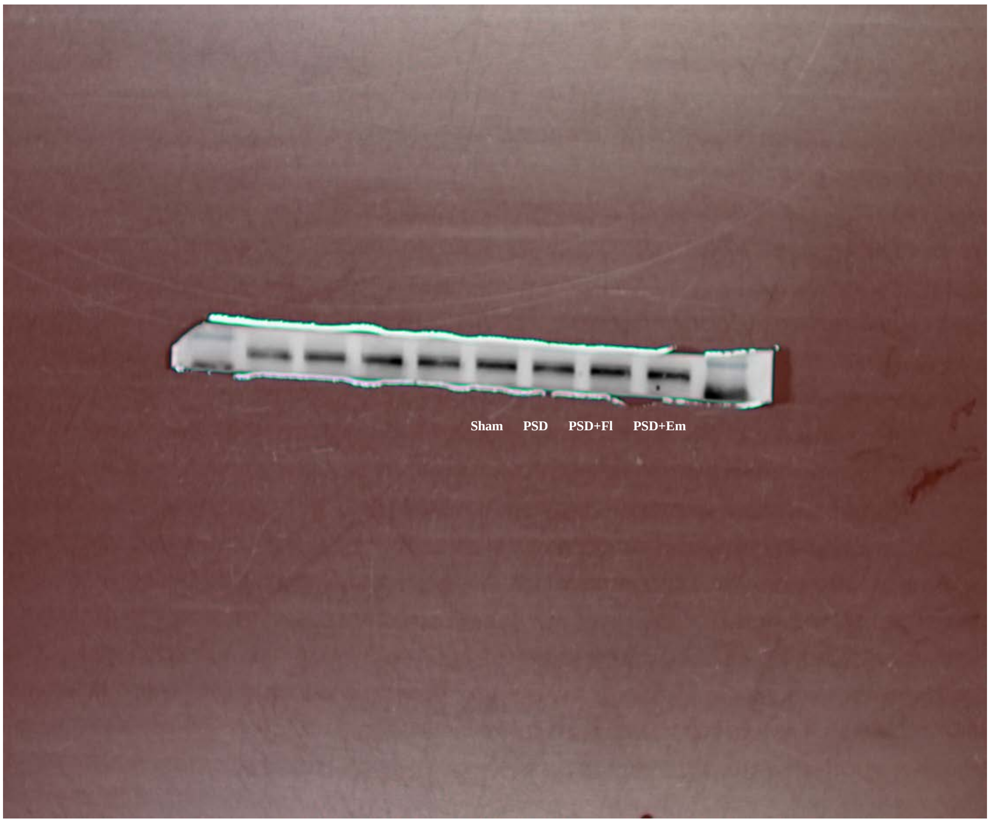

Furin-2'

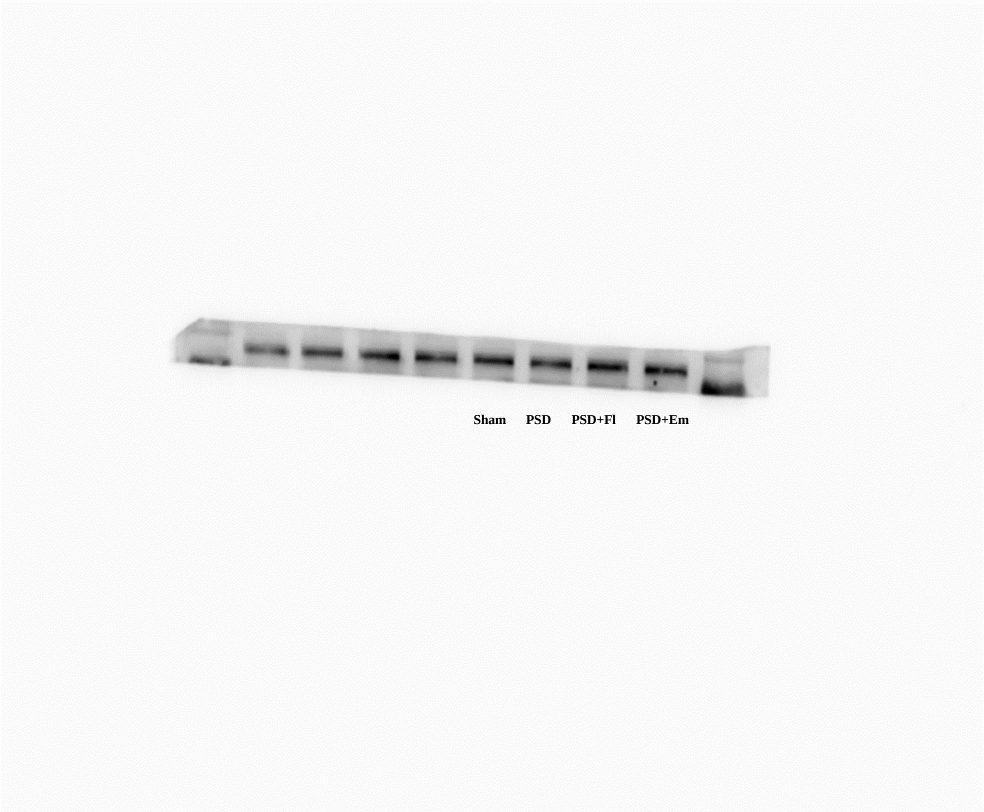

Furin-3

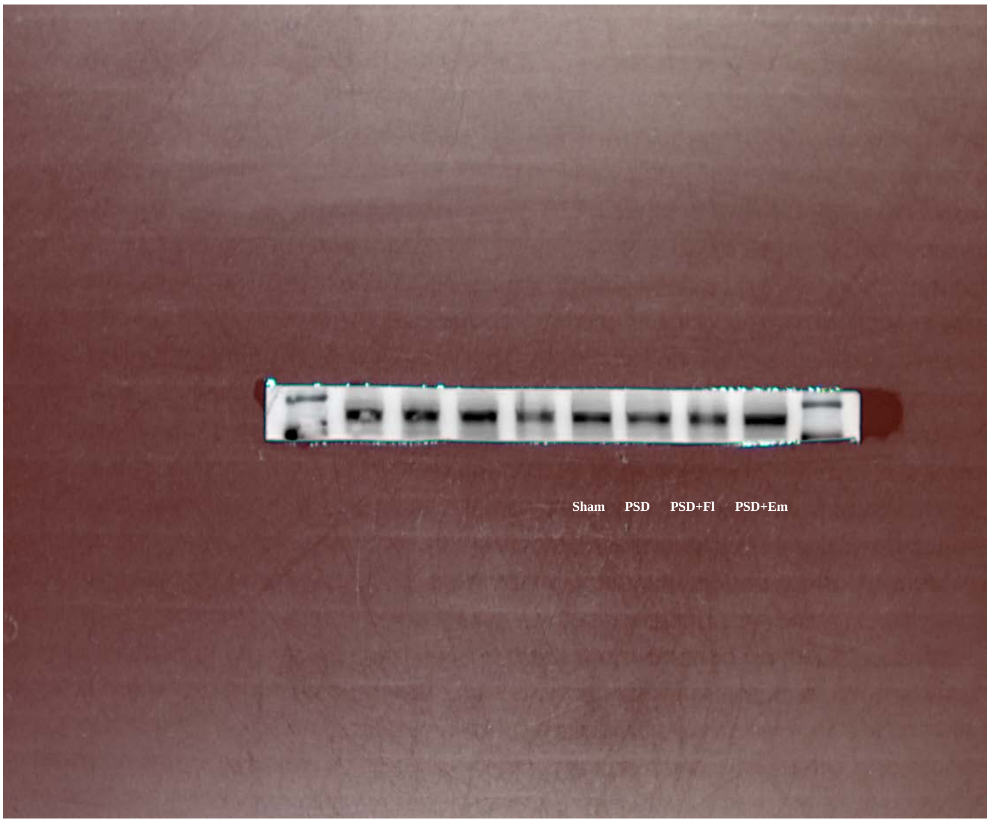

Furin-3′

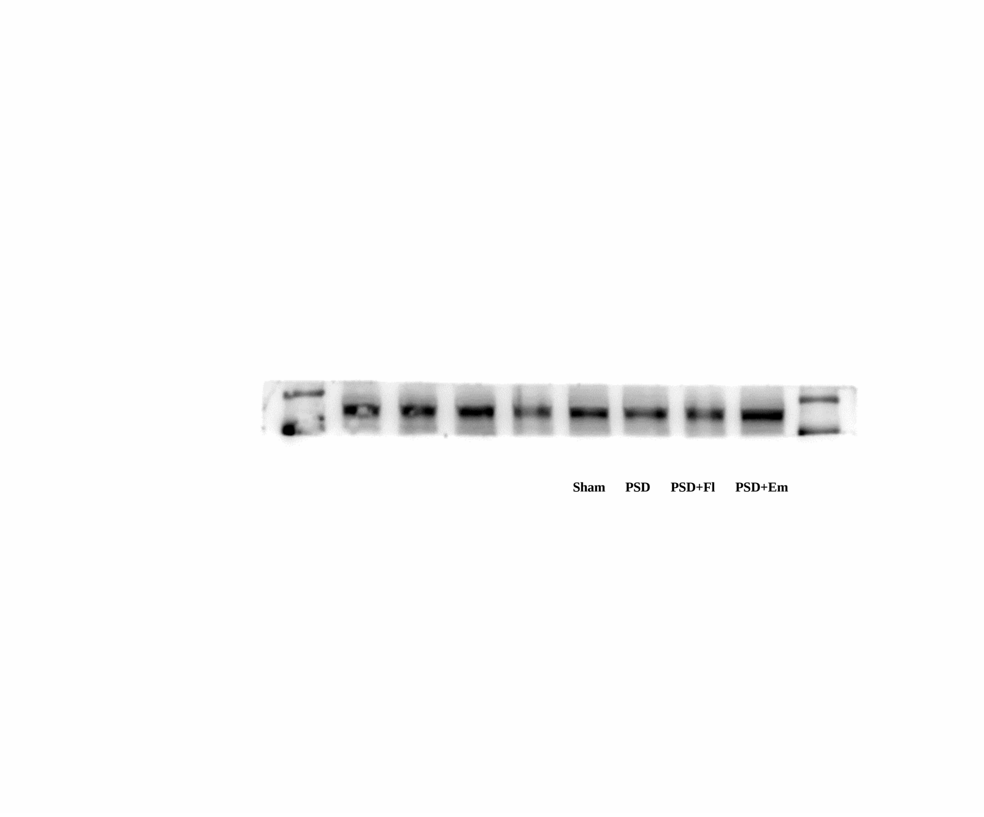

β-actin-1

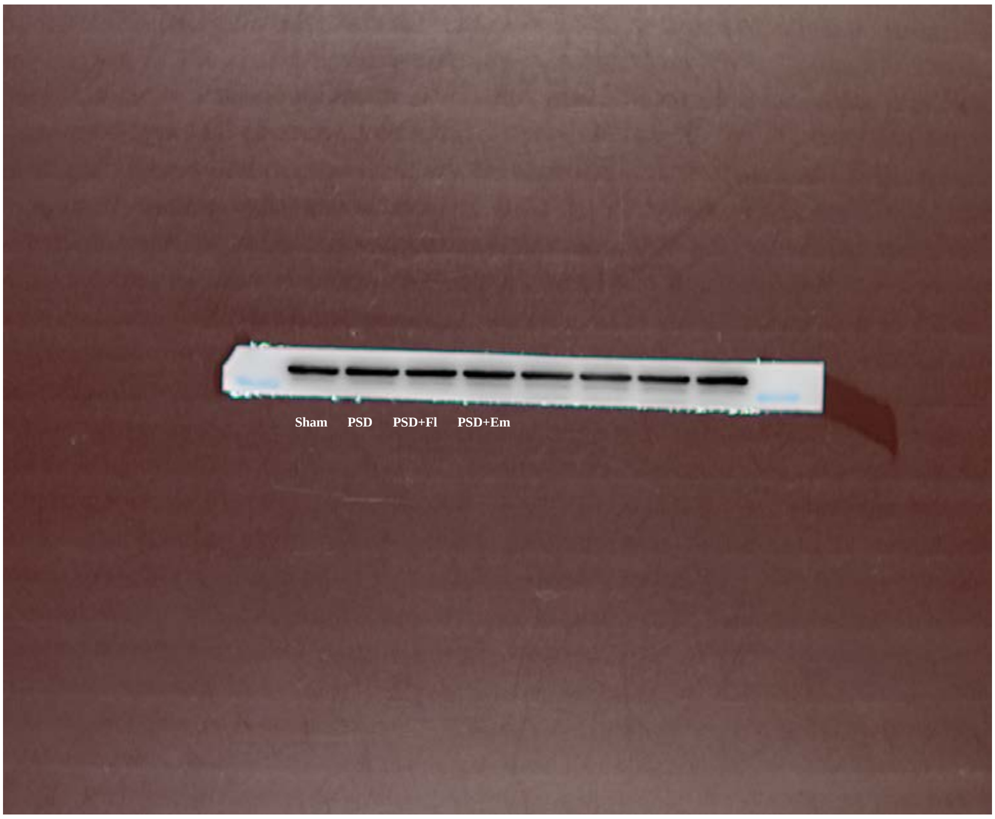

β-actin-1’

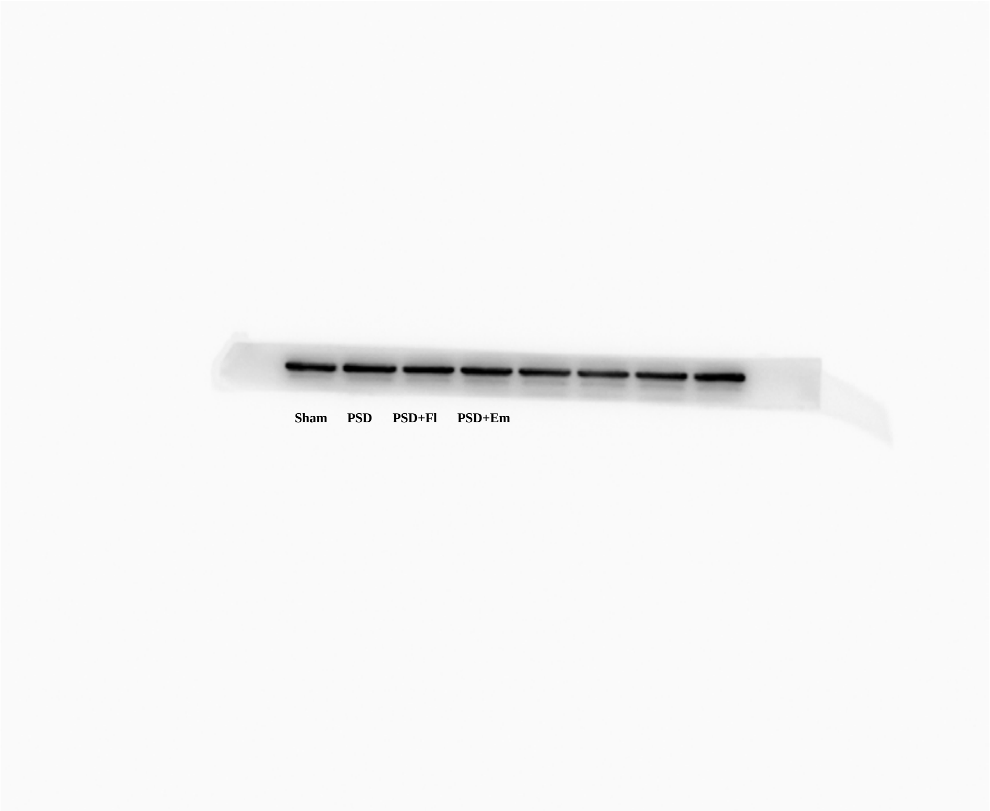

β-actin-2

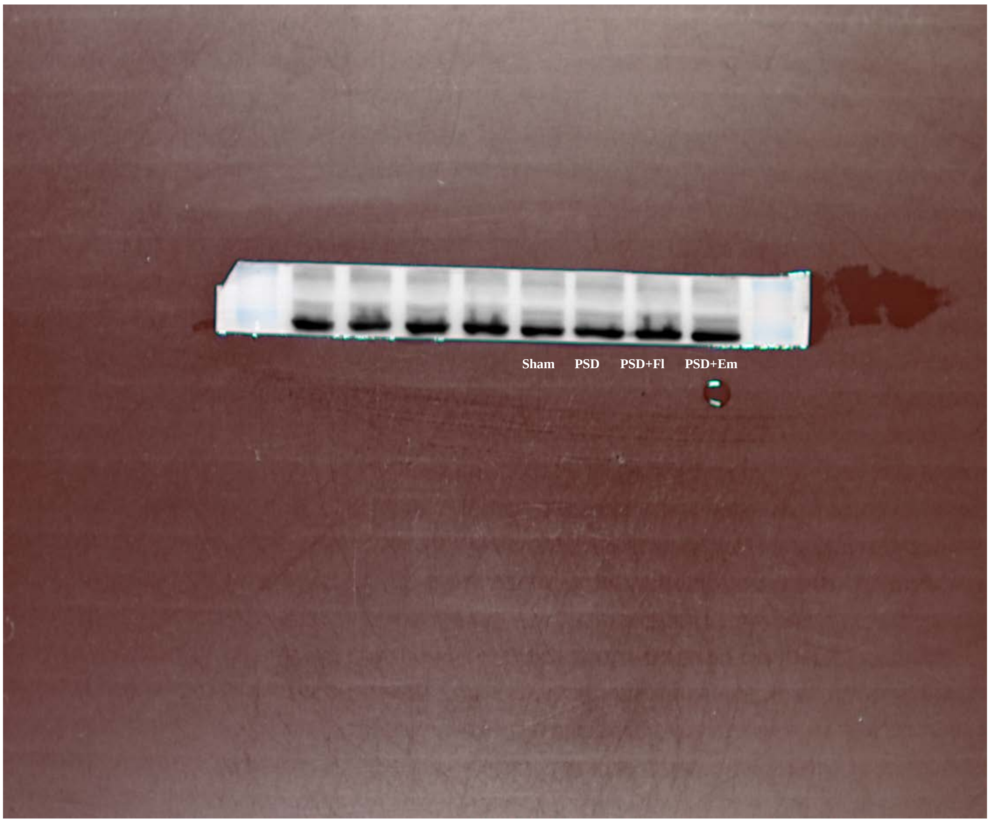

β-actin-2’

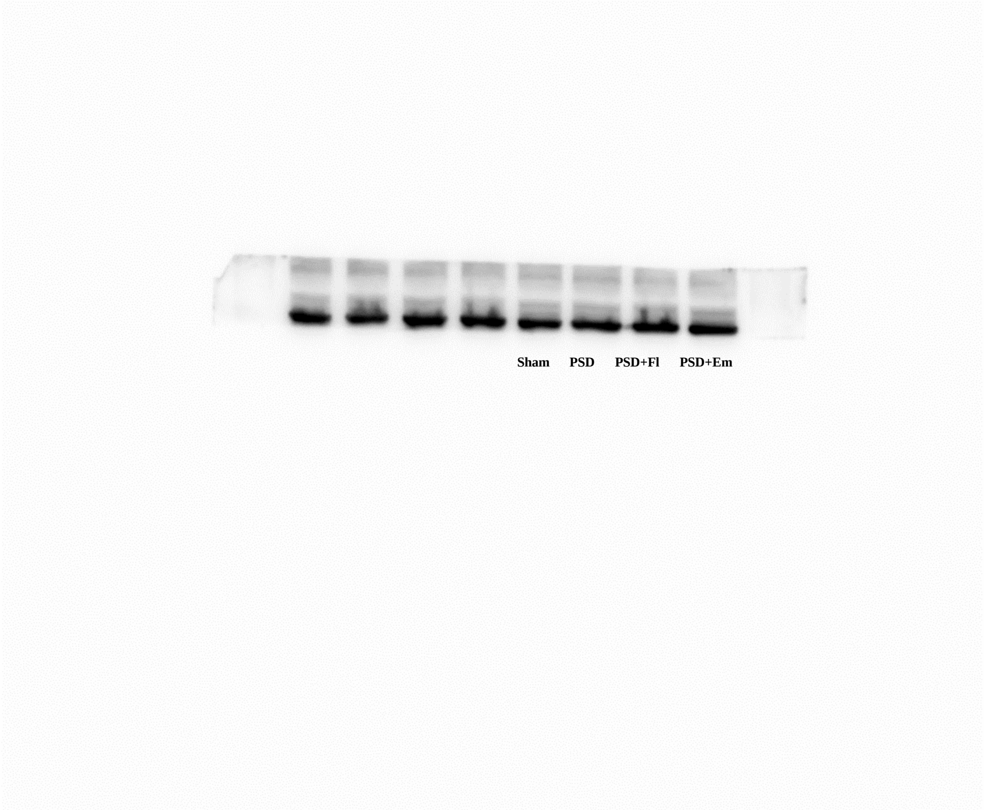

β-actin-3

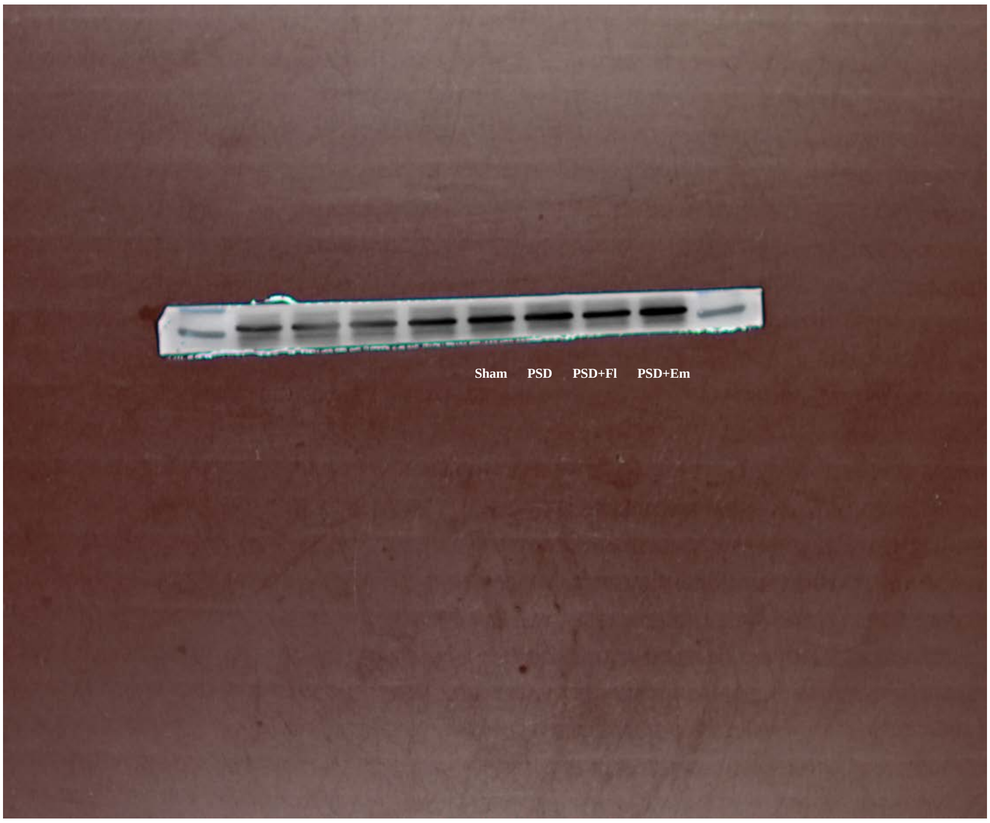

β-actin-3’

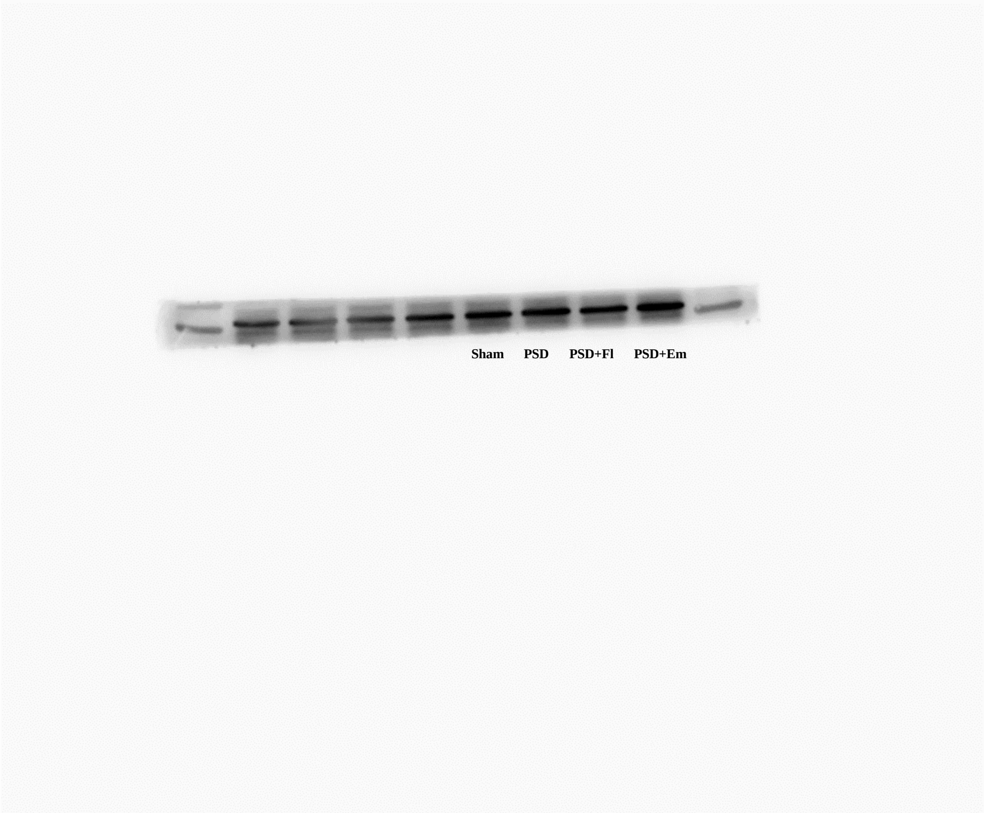

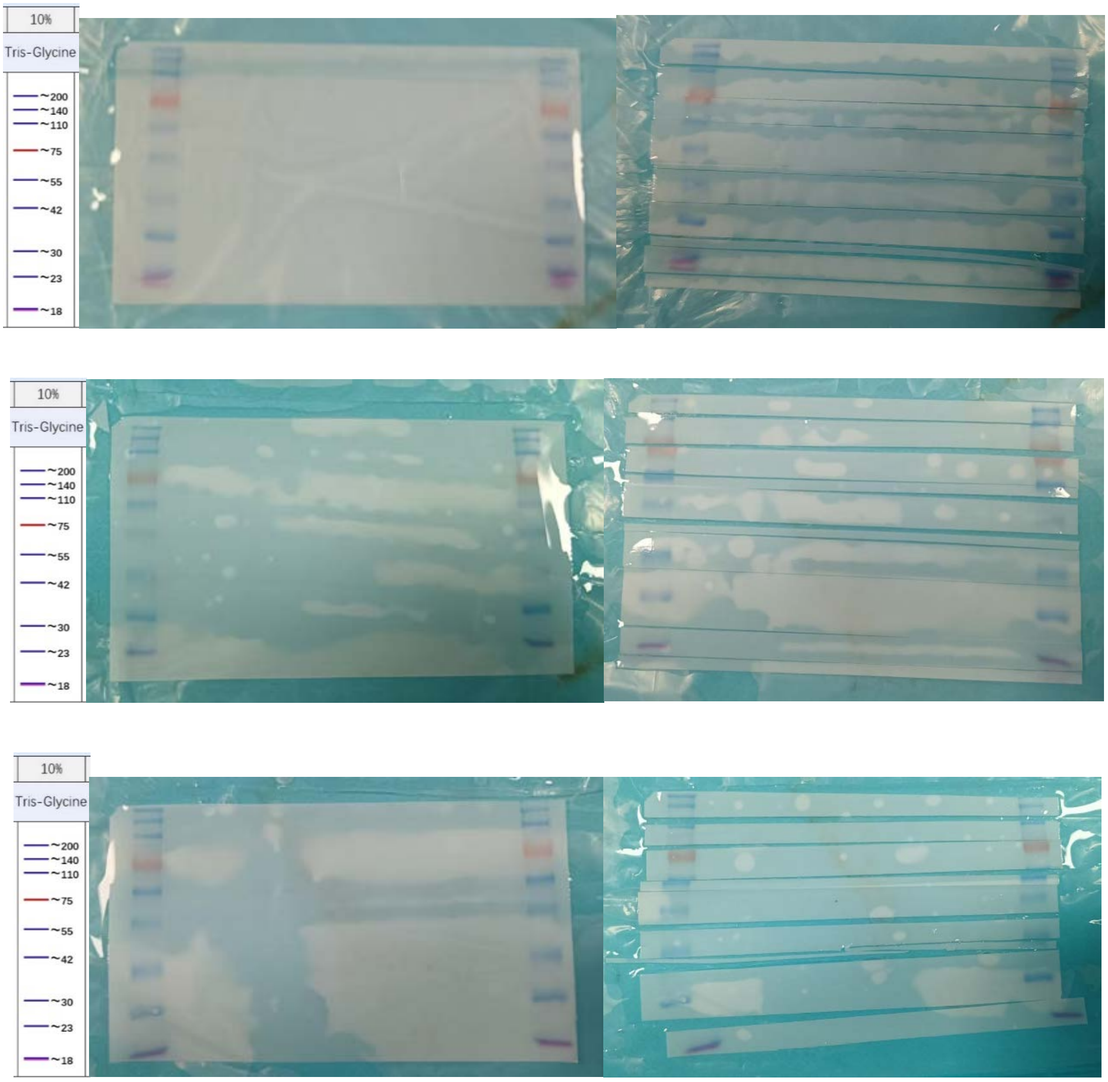

Figure 10E Complete unedited gel/imprint

MMP9-1

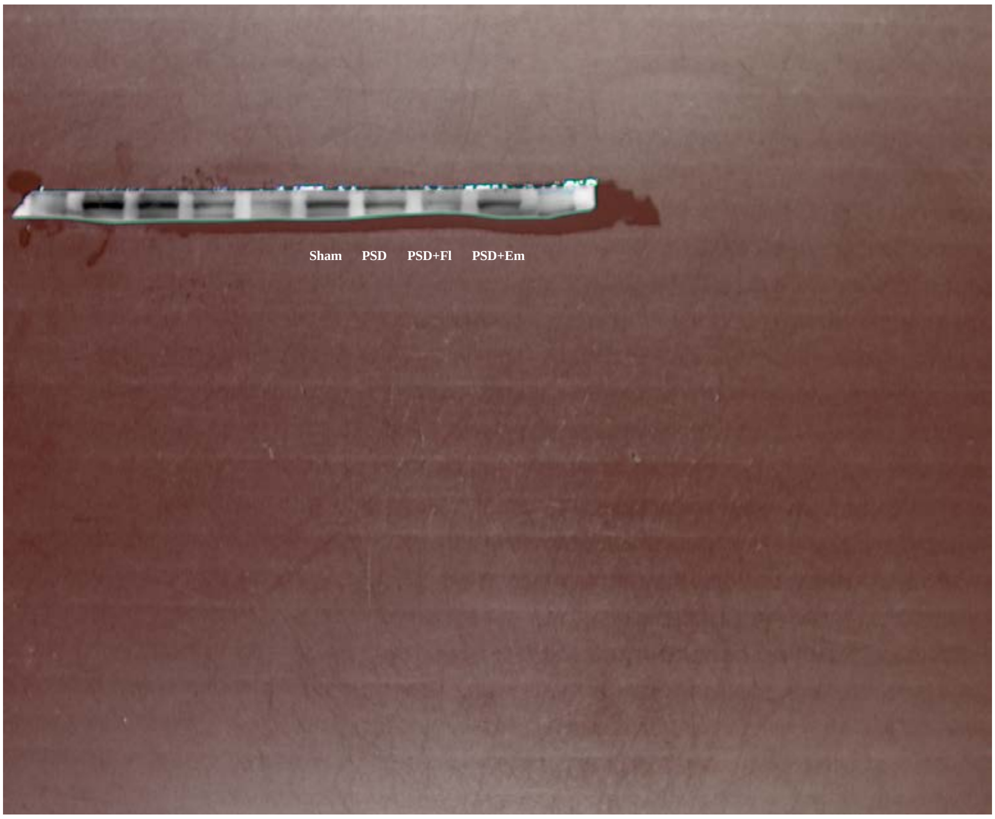

MMP9-1′

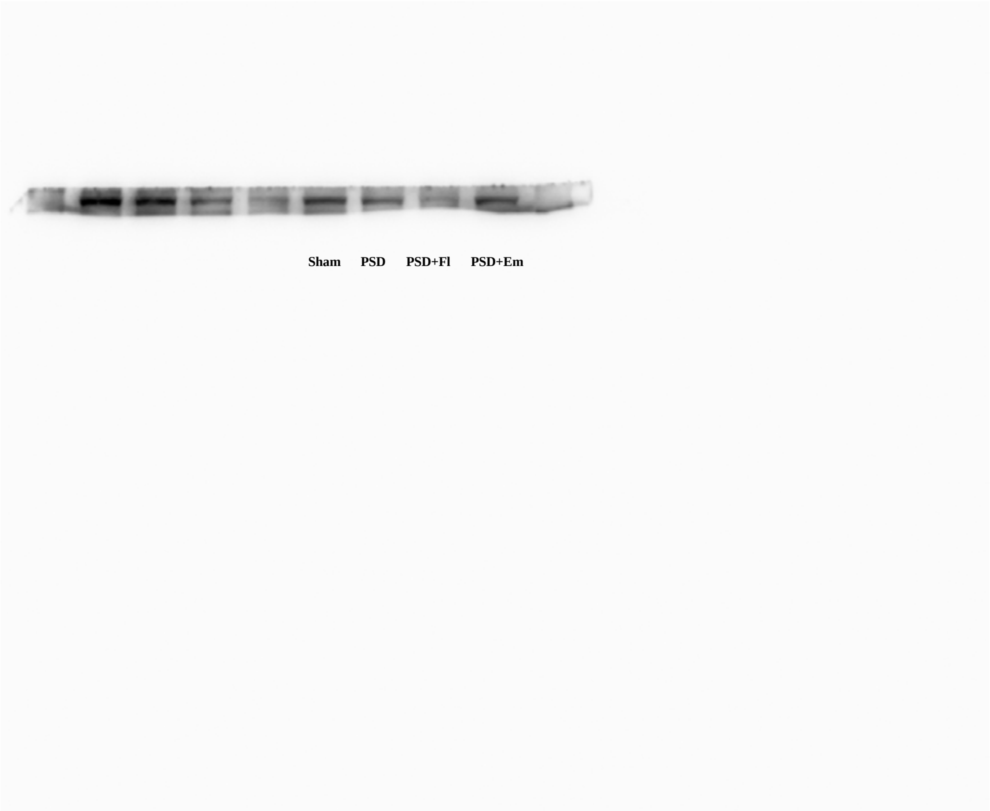

MMP9-2

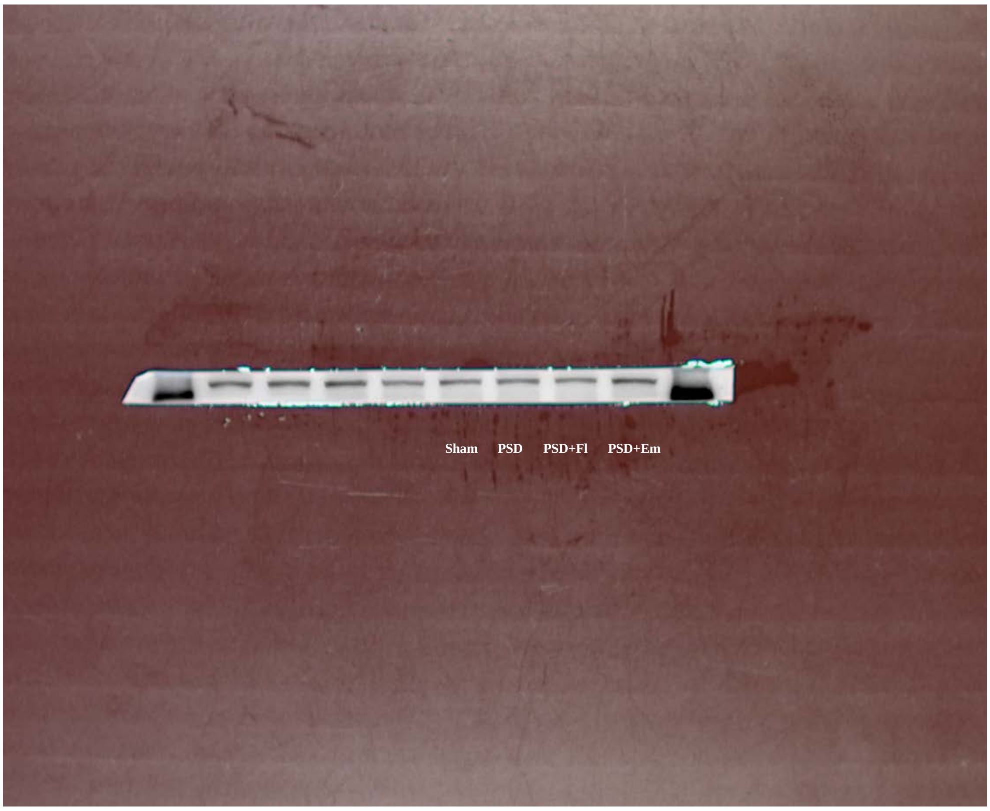

MMP9-2’

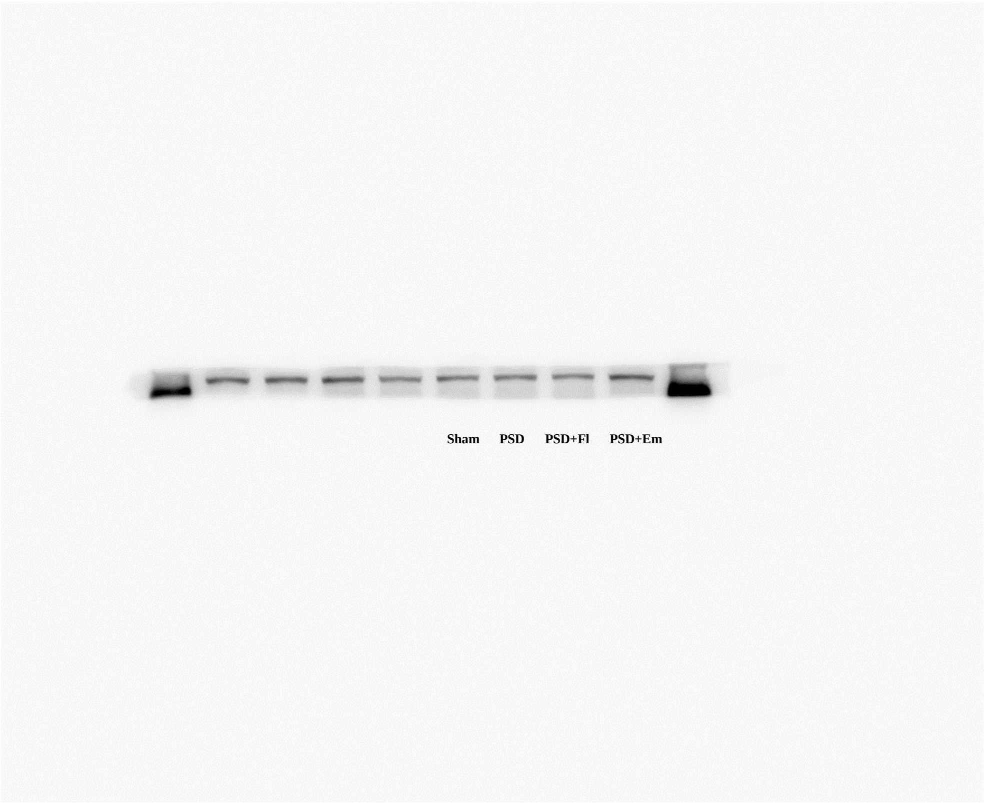

MMP9-3

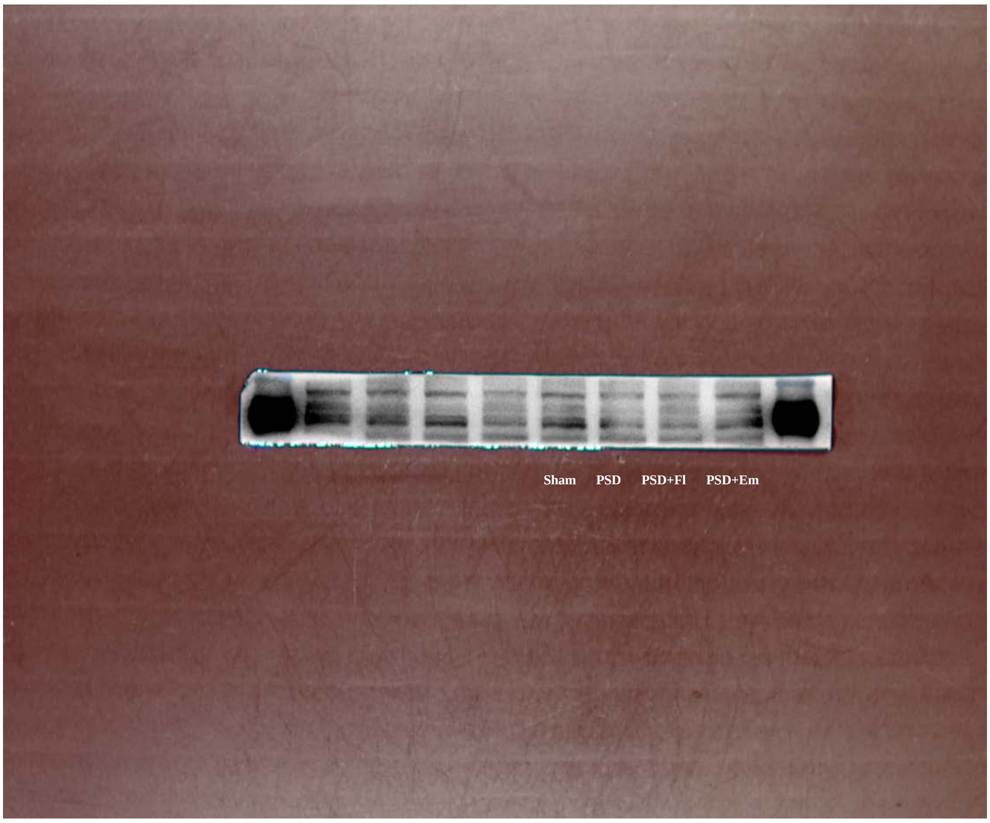

MMP9-3′

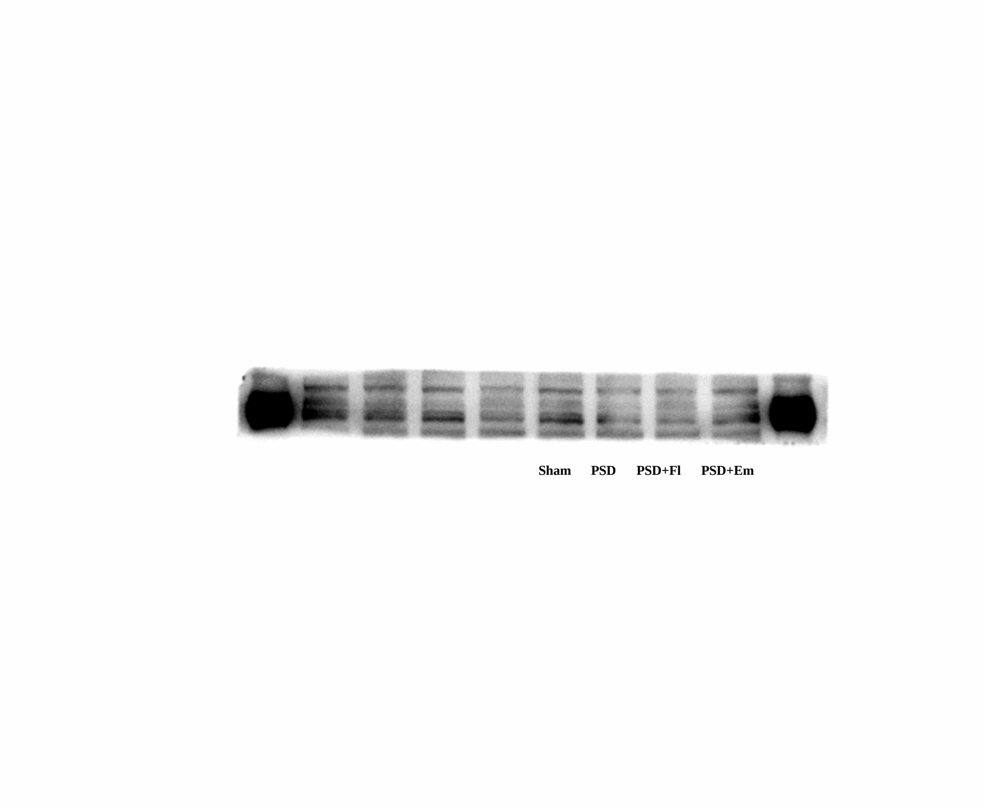

PC2-1

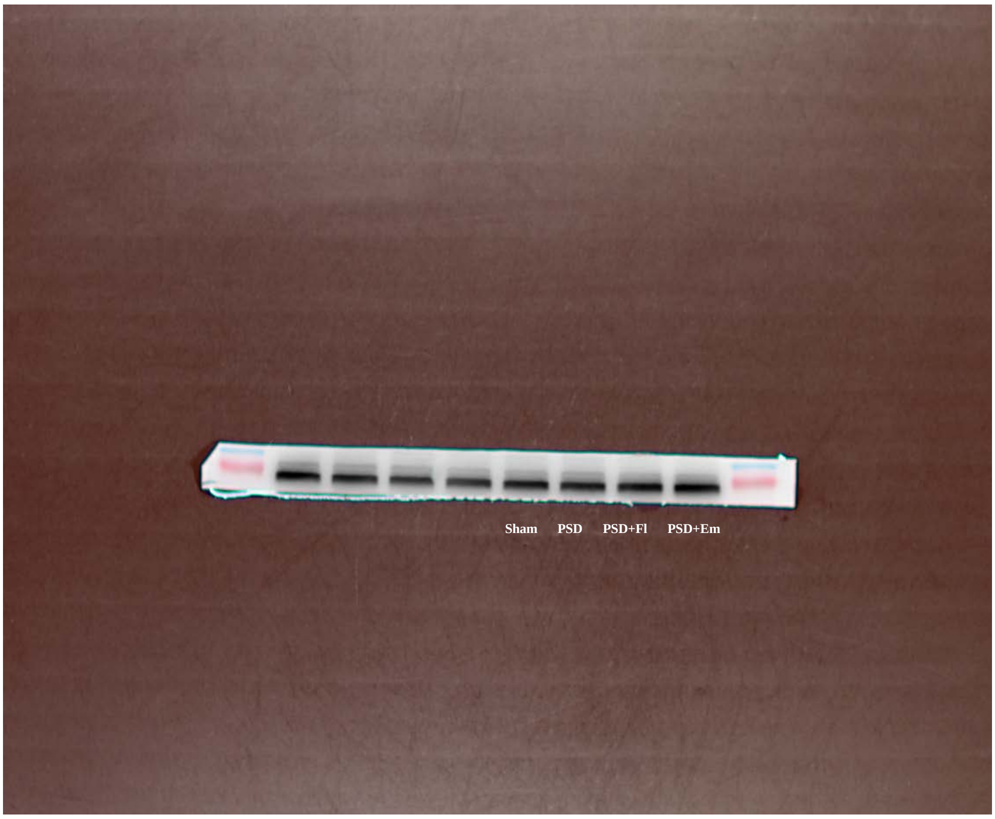

PC2-1’

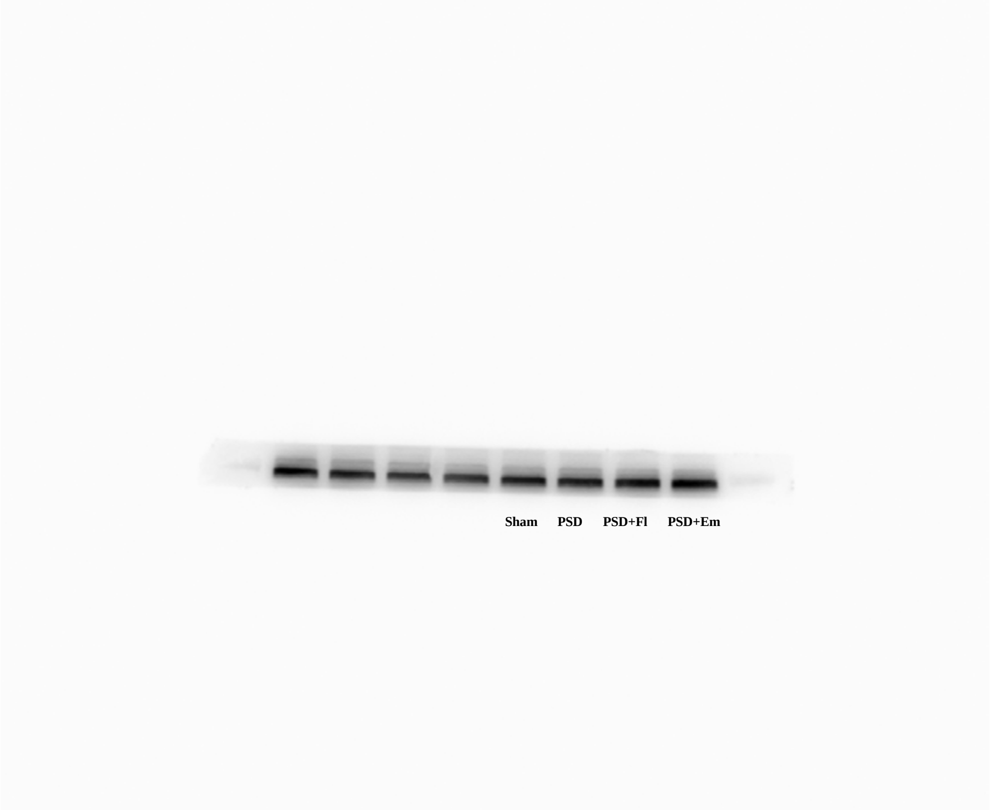

PC2-2

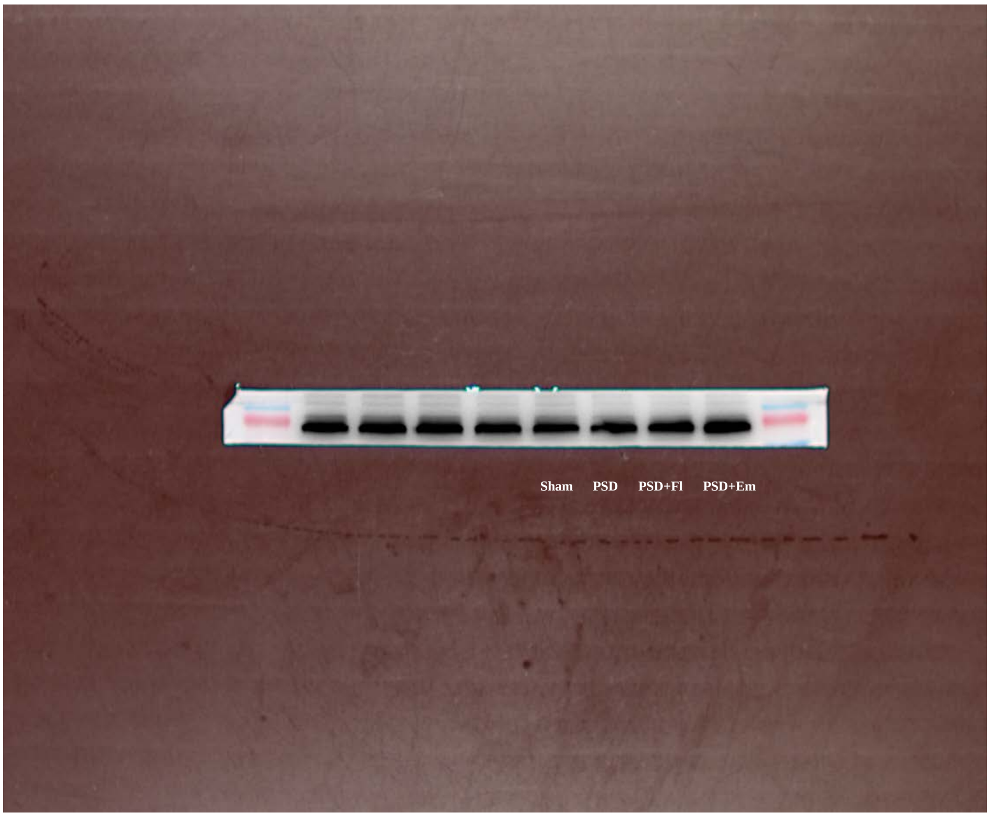

PC2-2’

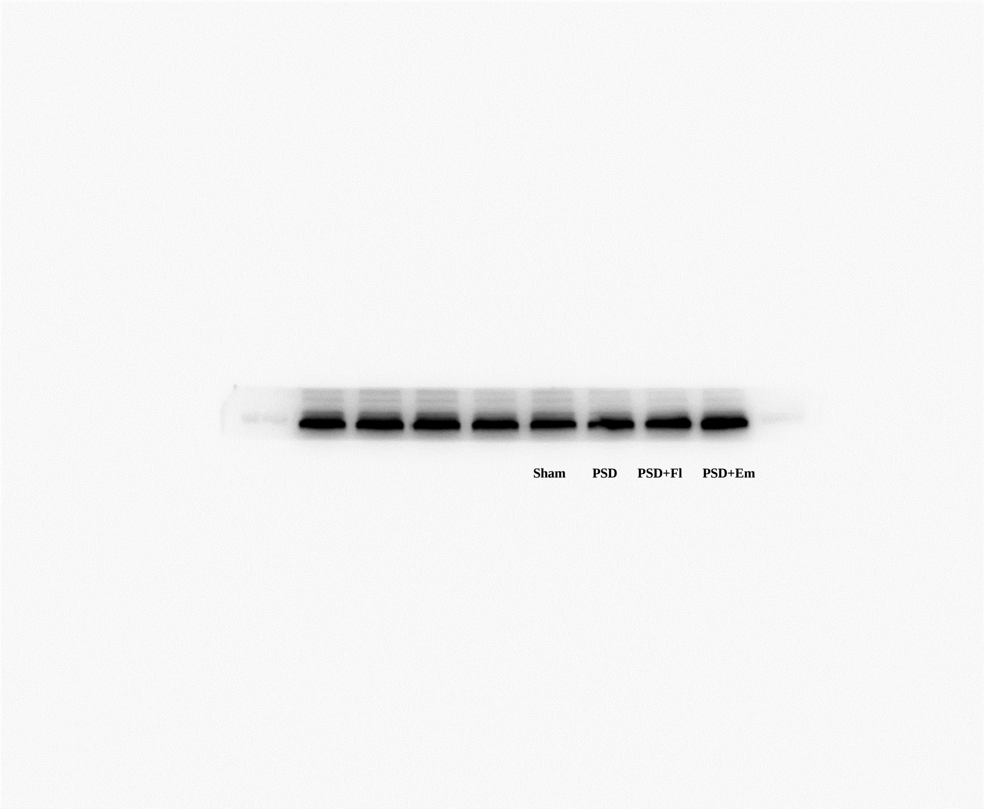

PC2-3

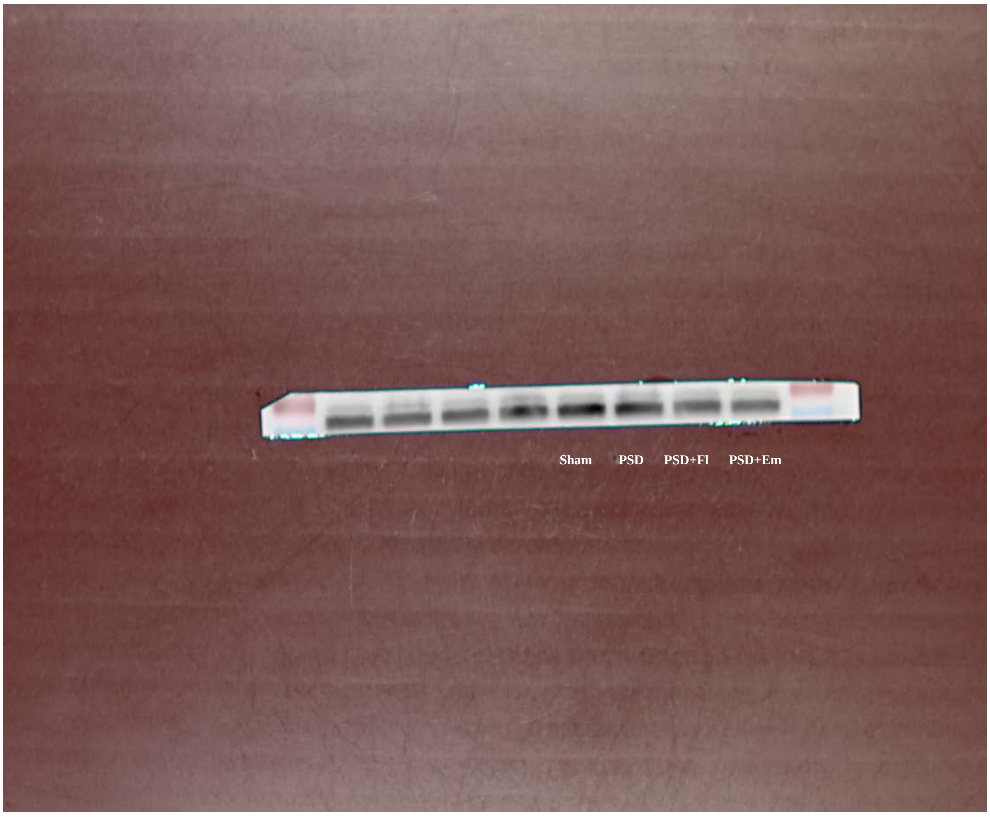

PC2-3’

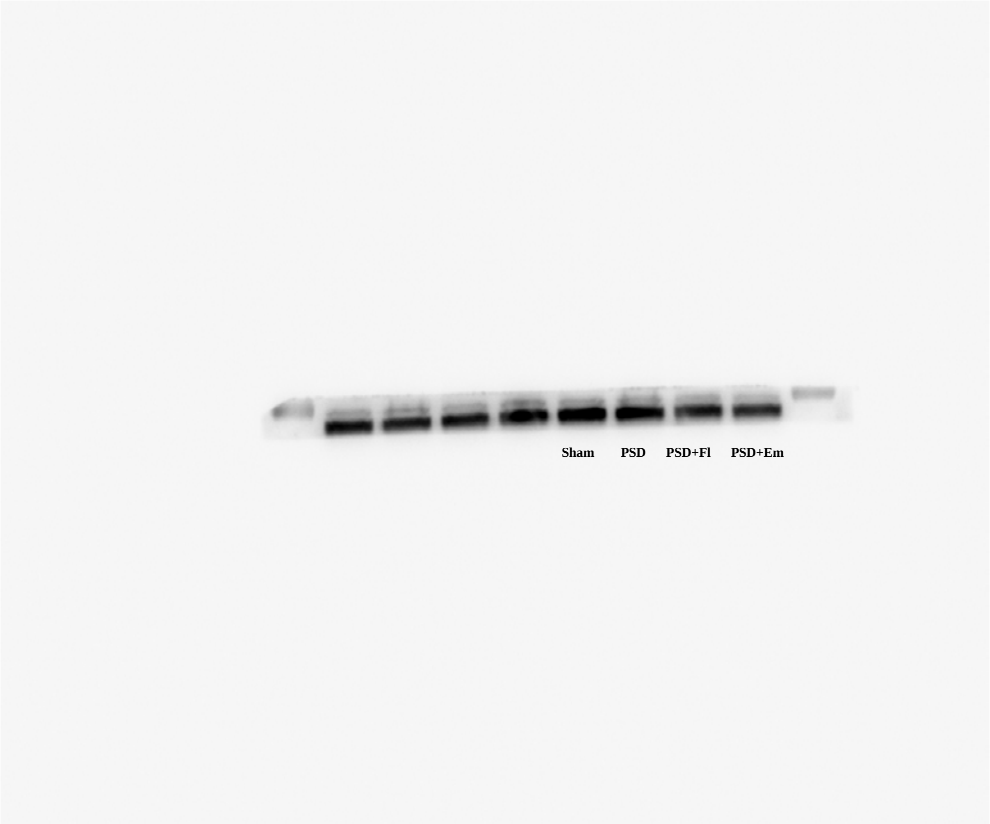

β-actin-1

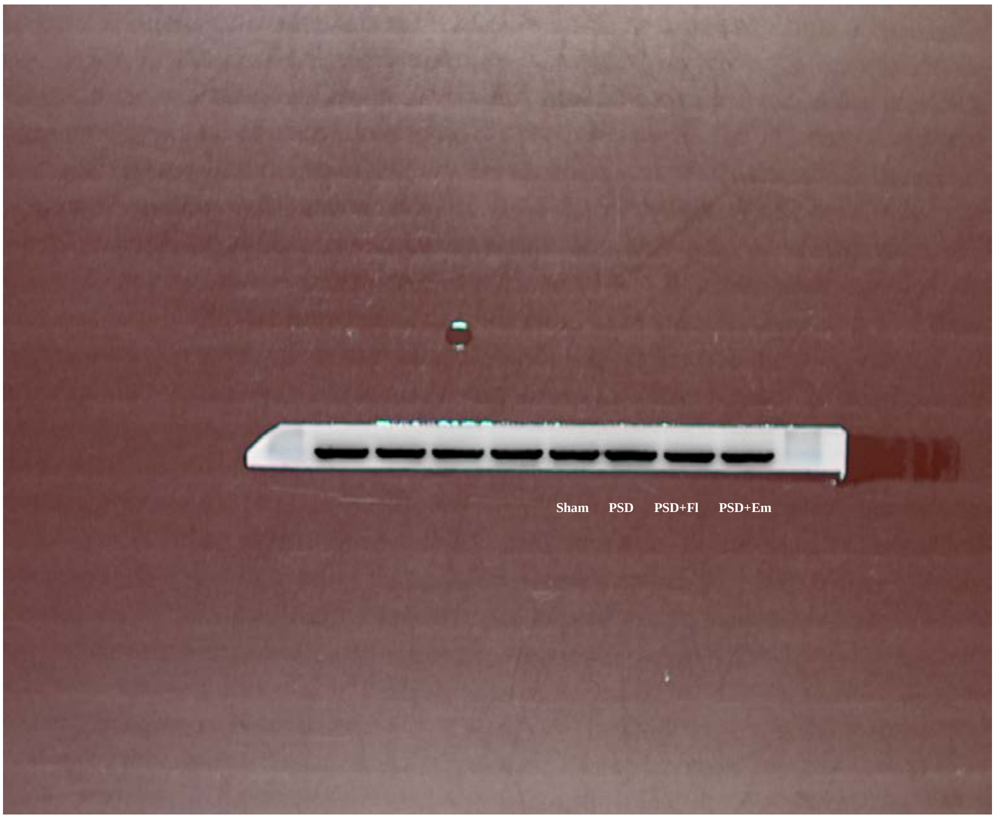

β-actin-1’

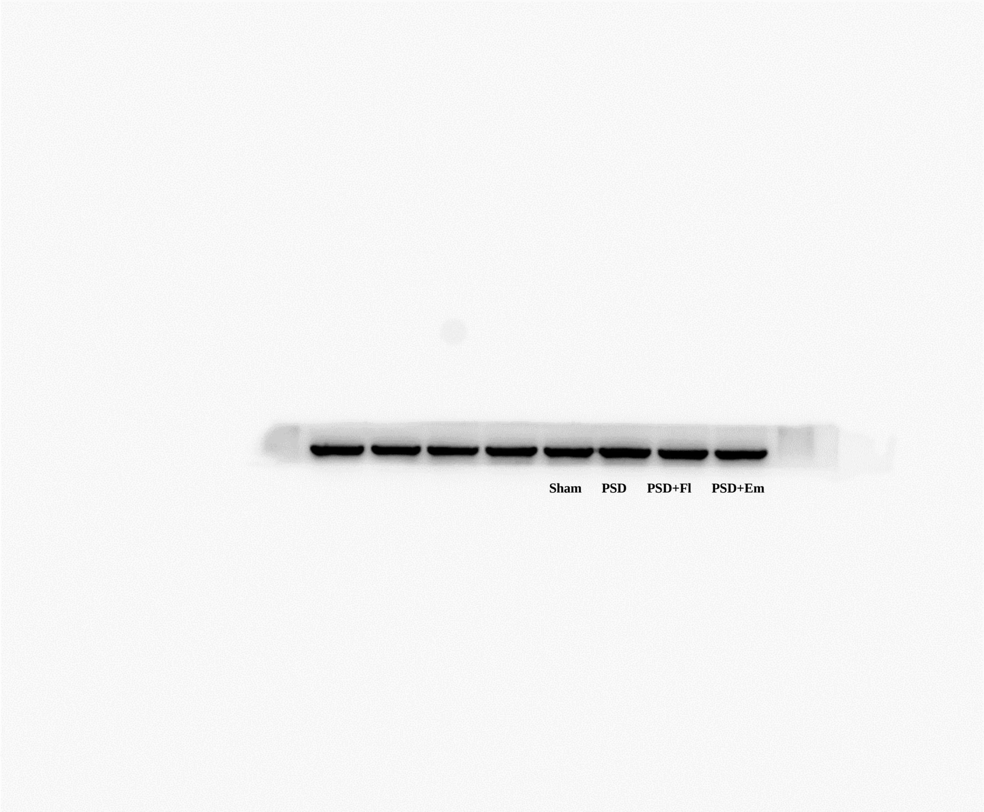

β-actin-2

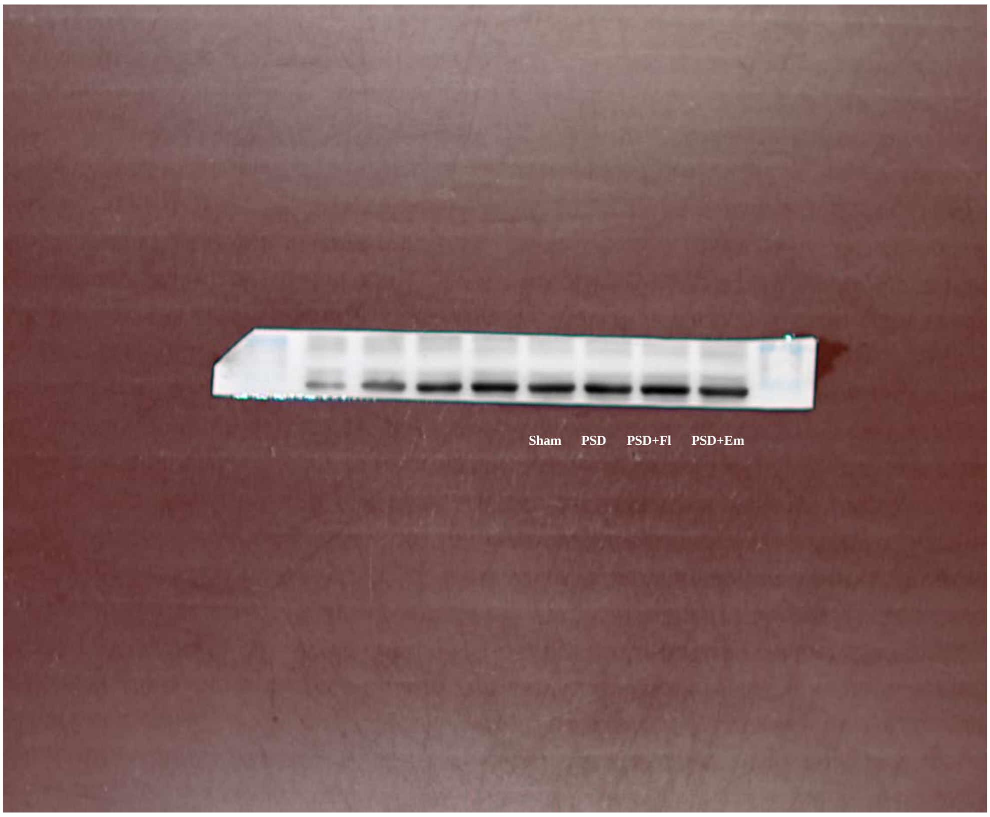

β-actin-2’

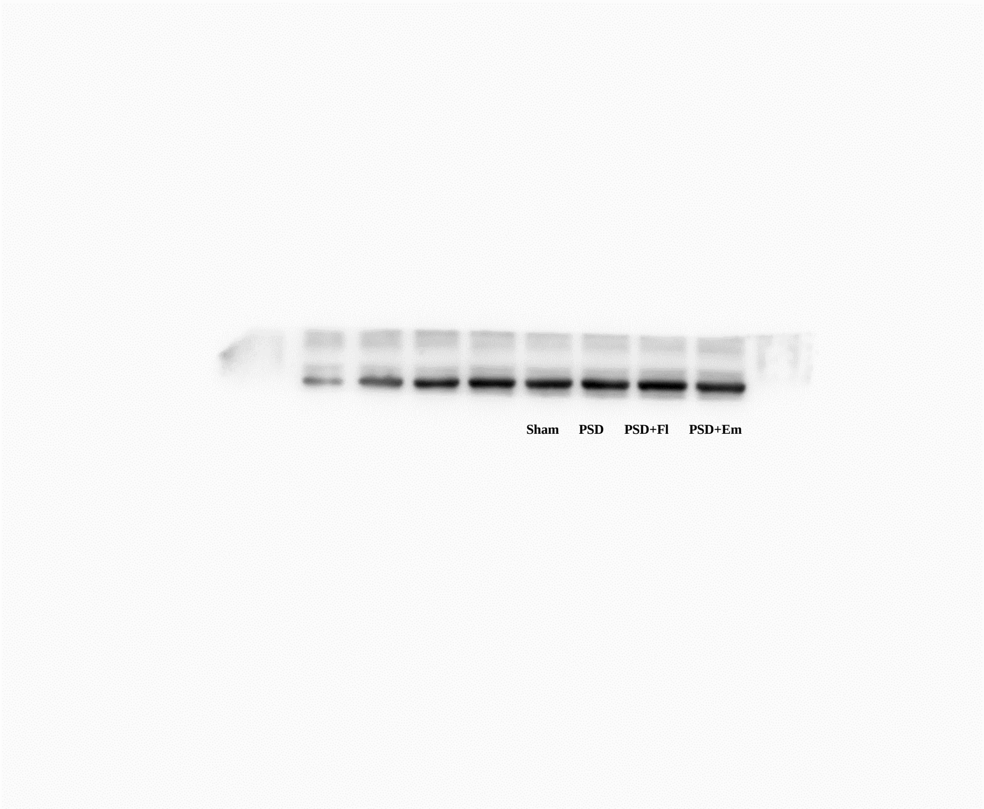

β-actin-3

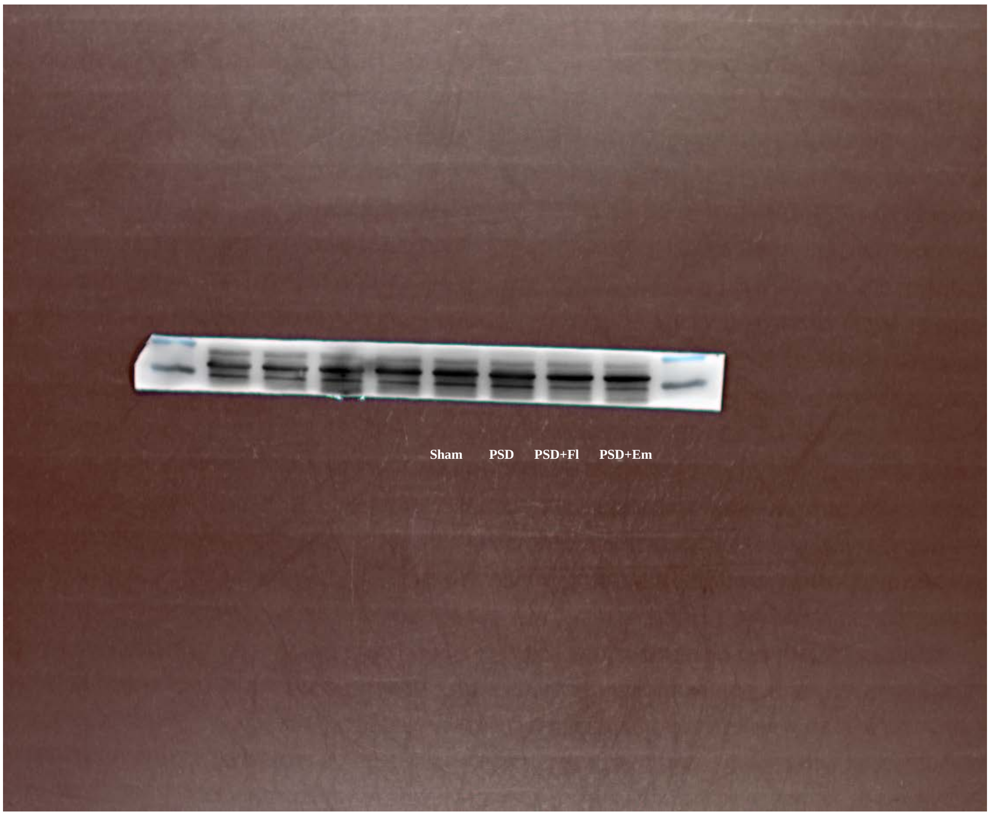

β-actin-3’

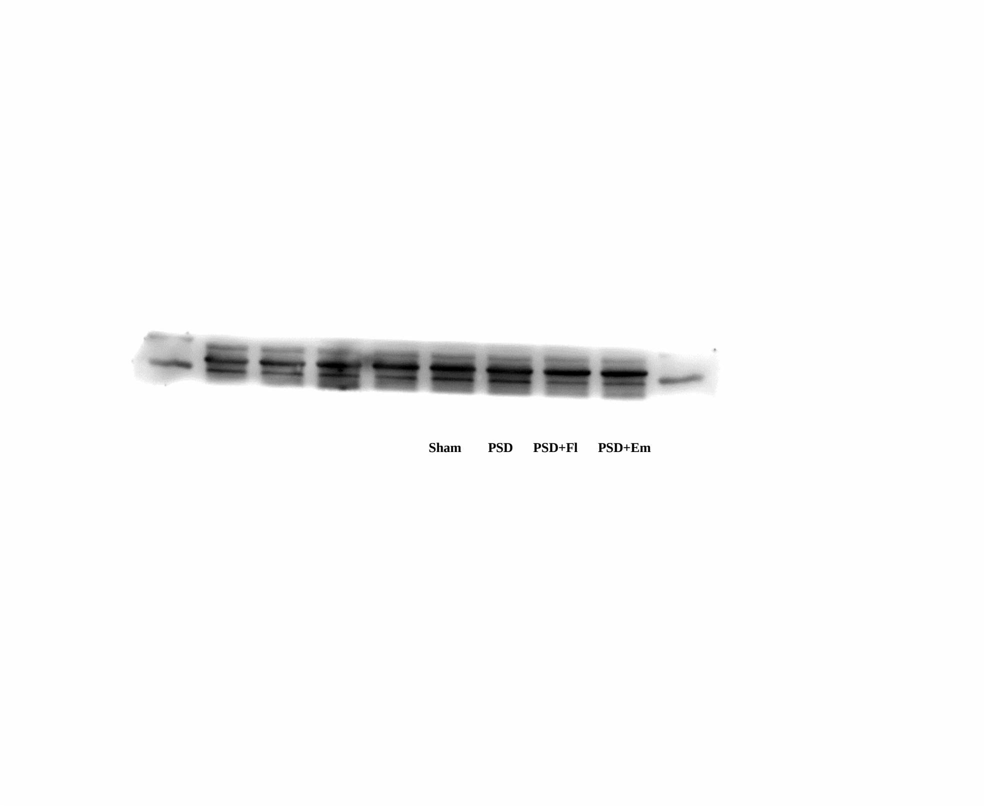

Figure.11 E tPA, Furin, MMP 9, and PC proteins in the mPFC

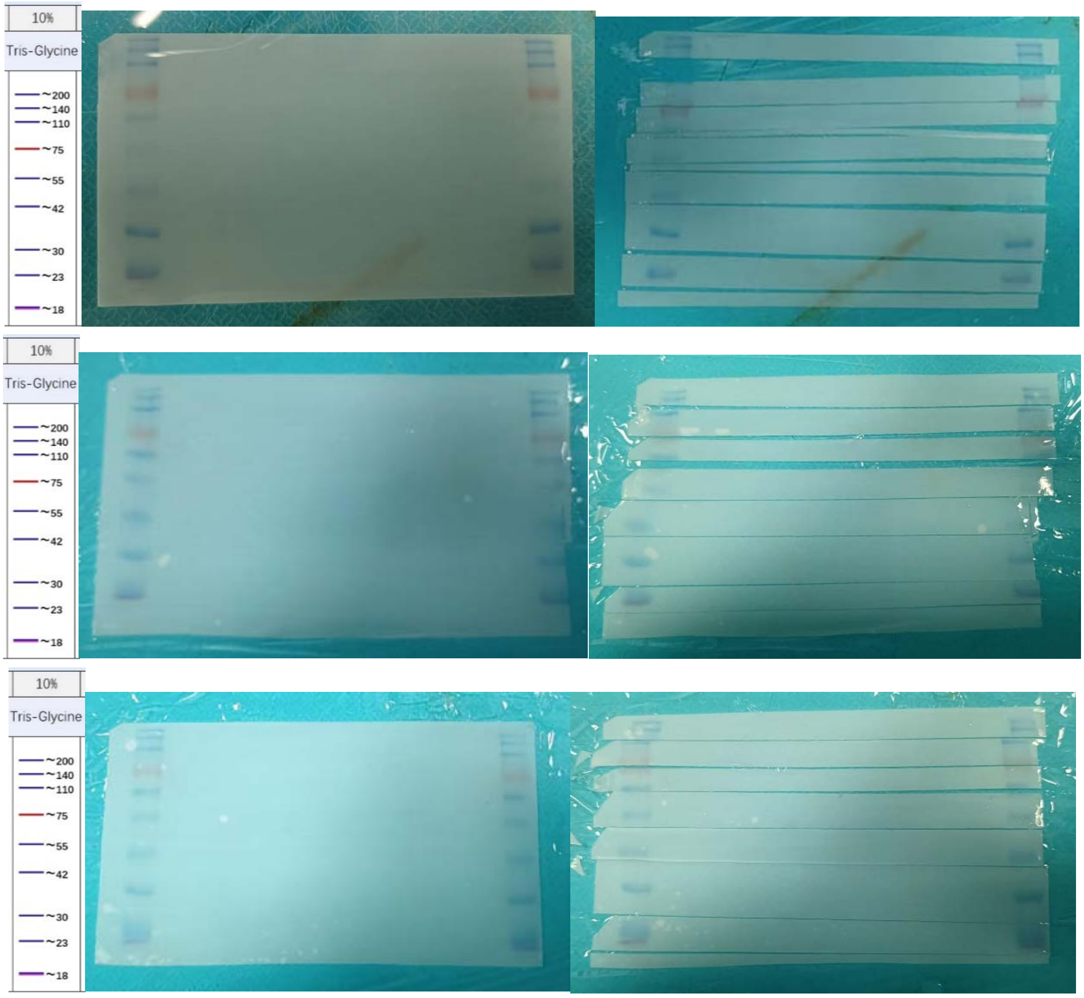

Figure 11E Complete unedited gel/imprint

tPA-1

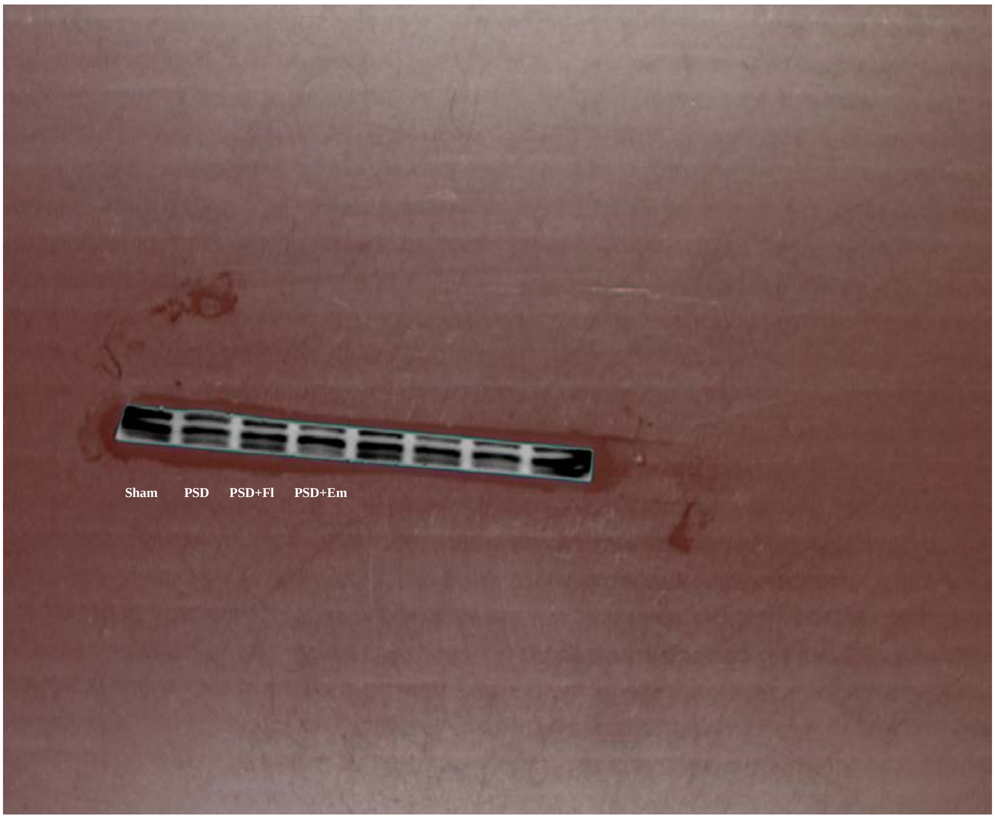

tPA-1′

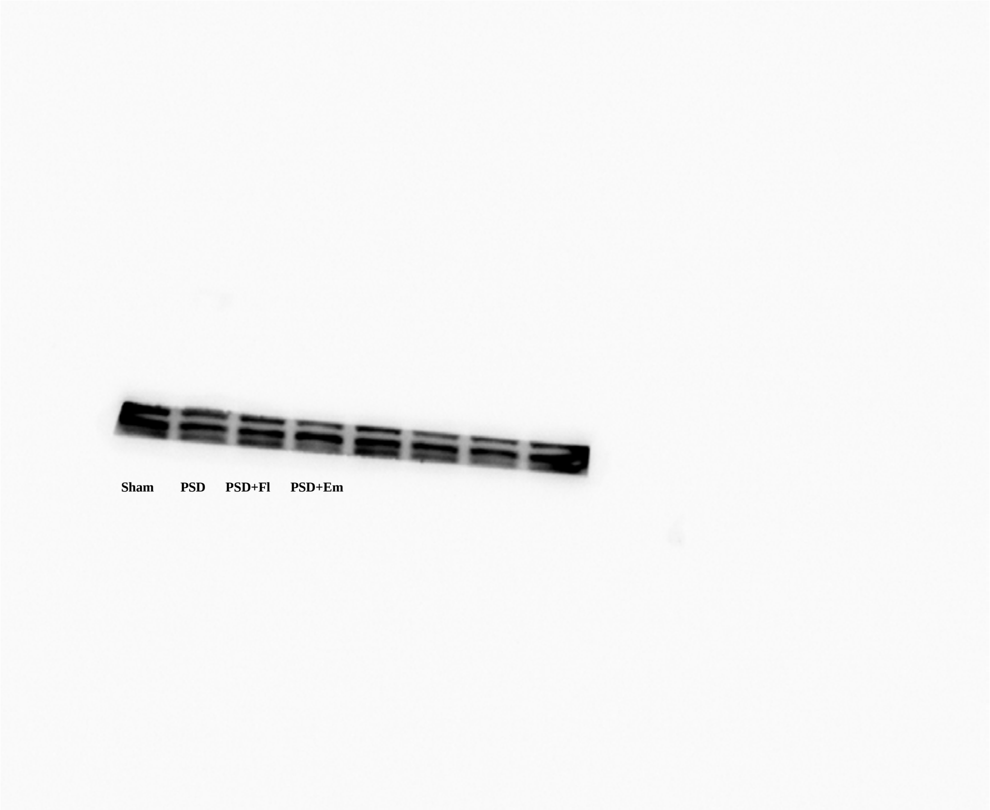

tPA-2

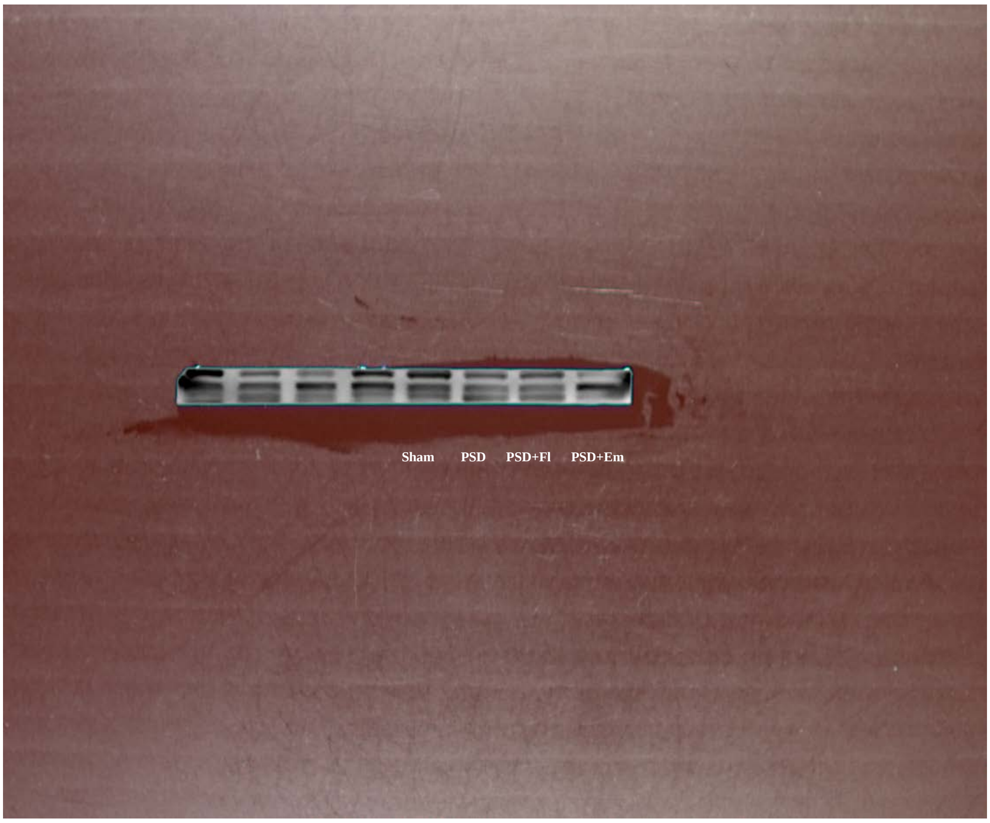

tPA-2’

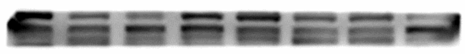

tPA-3

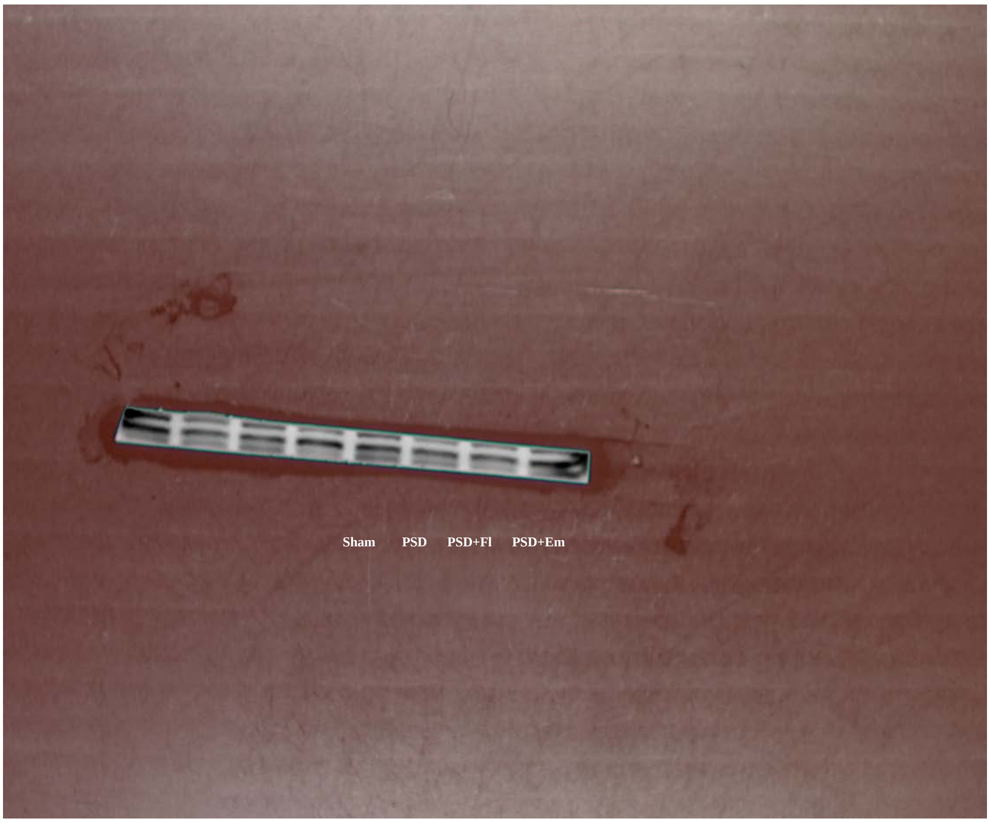

tPA-3’

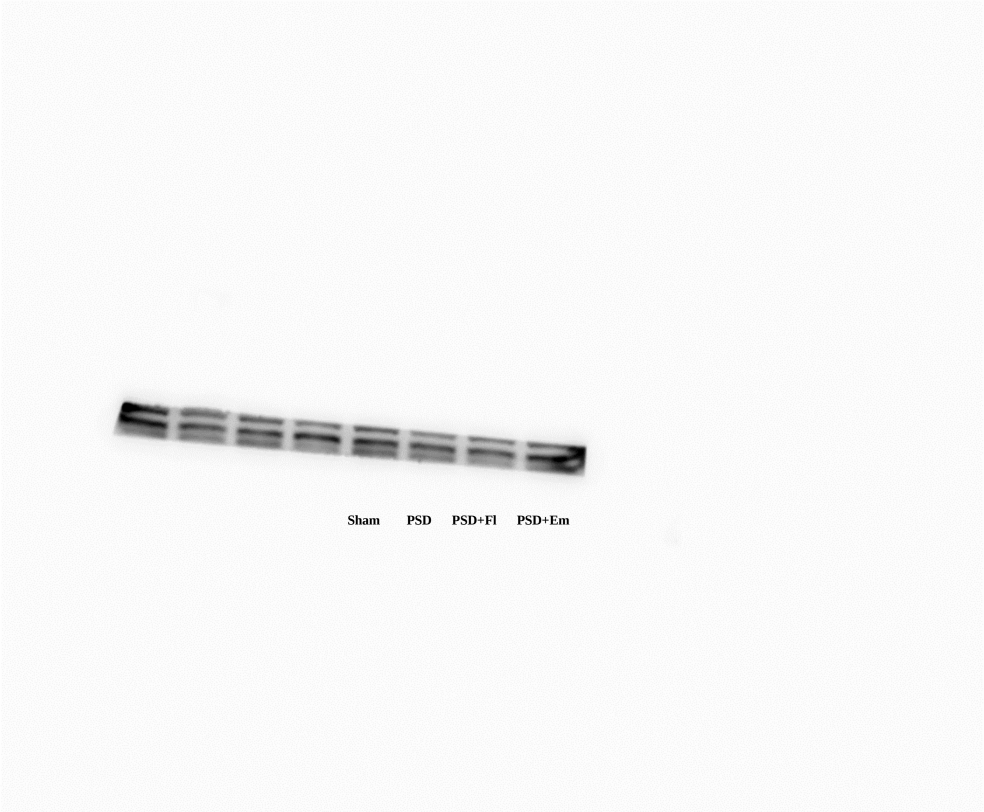

Furin-1

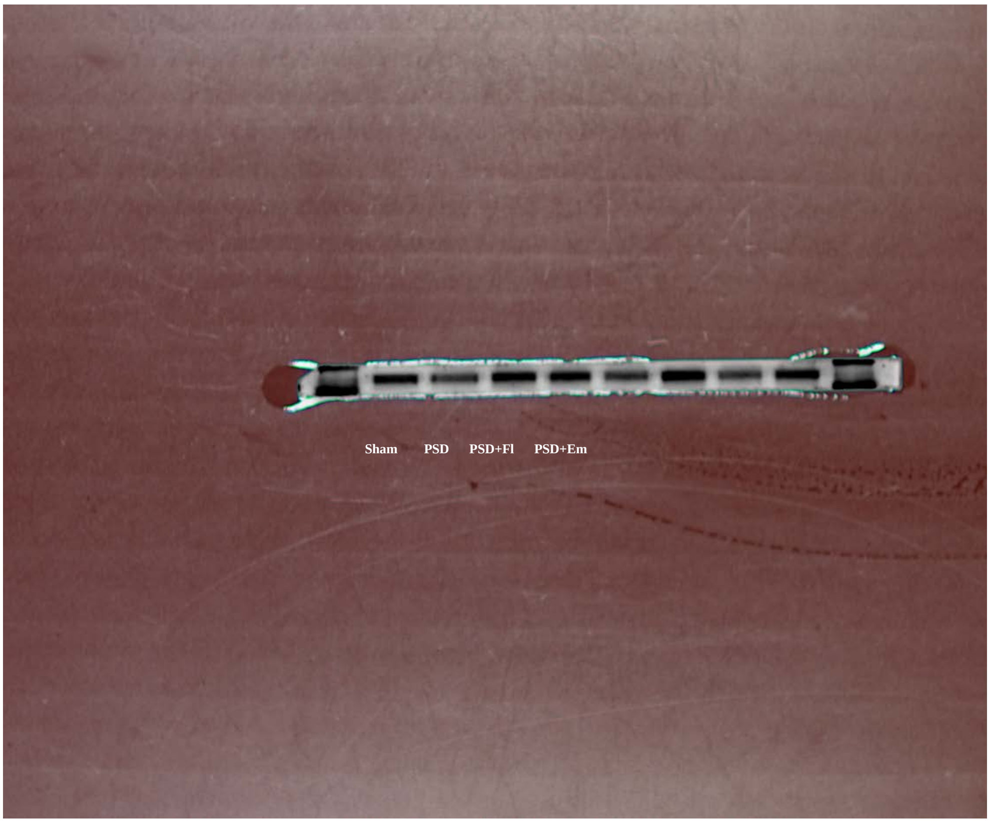

Furin-1′

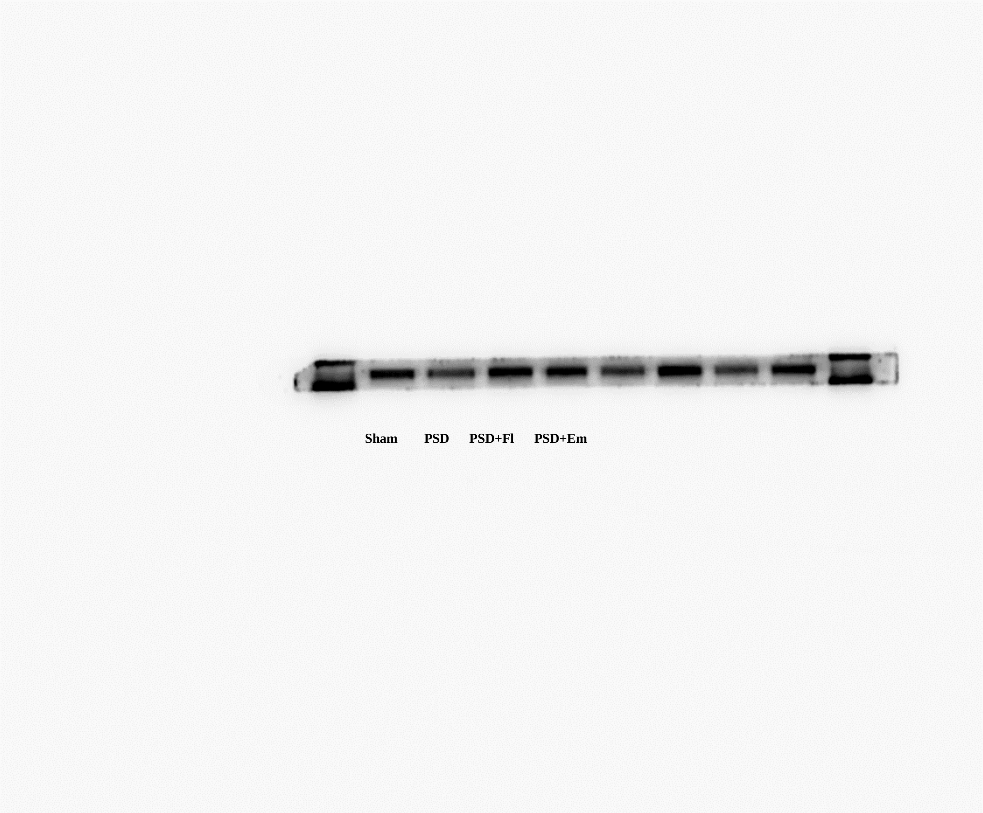

Furin-2

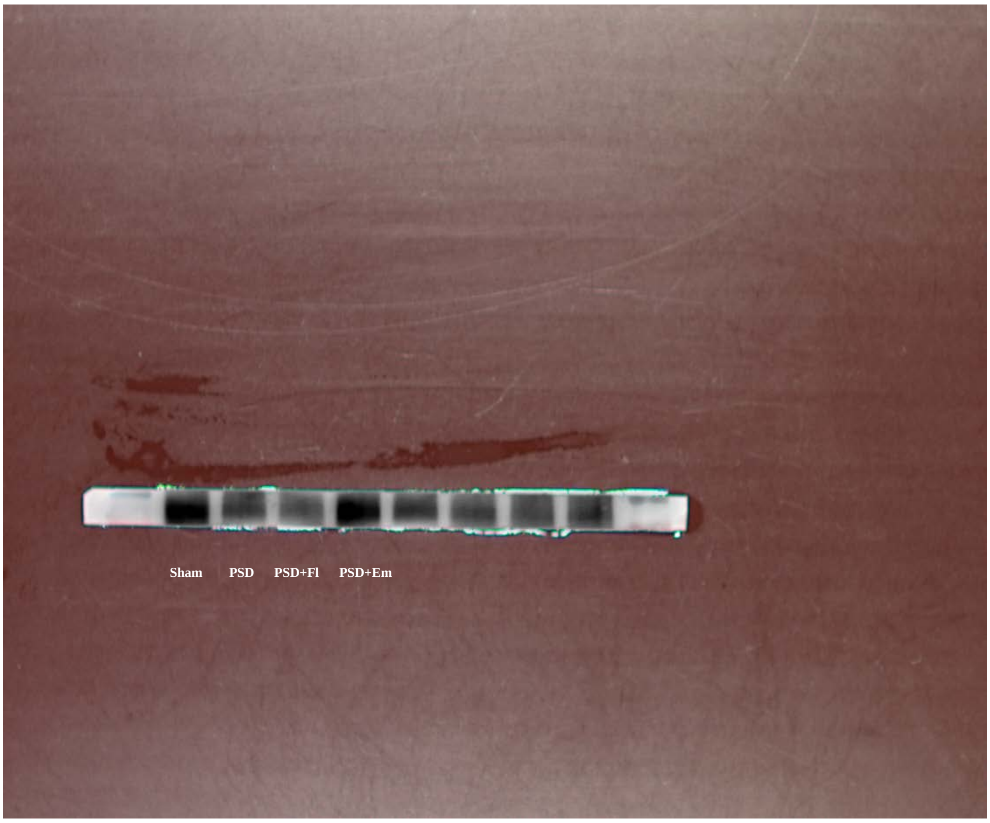

Furin-2’

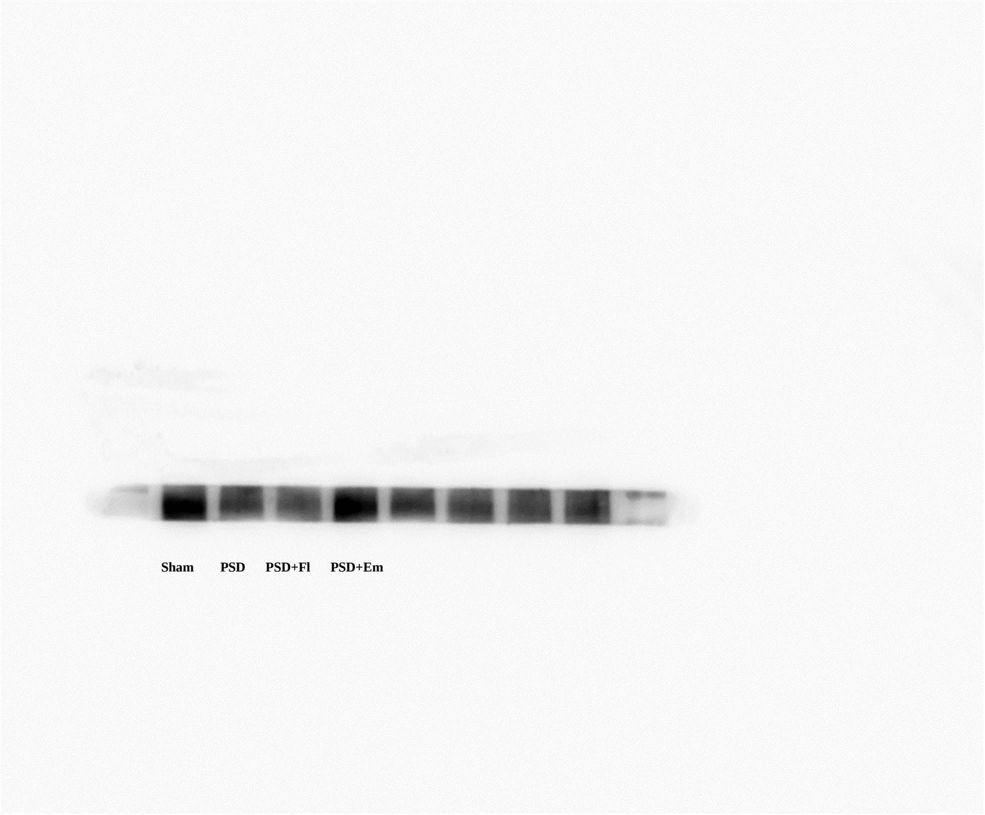

Furin-3

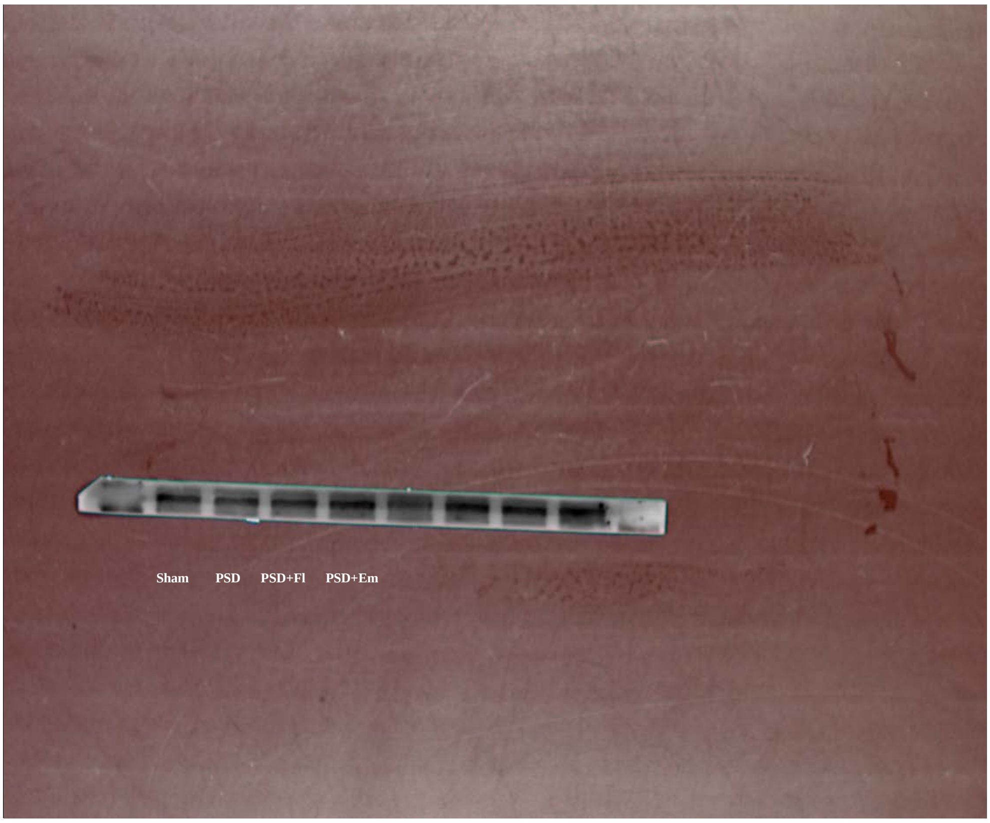

Furin-3′

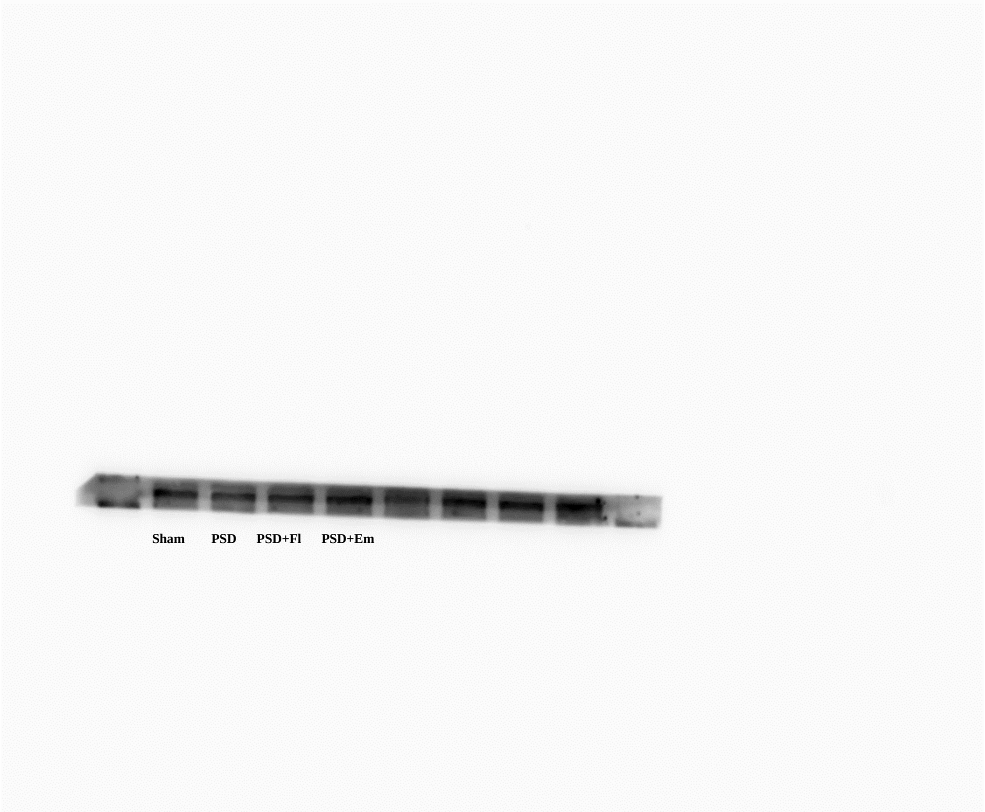

β-actin-1

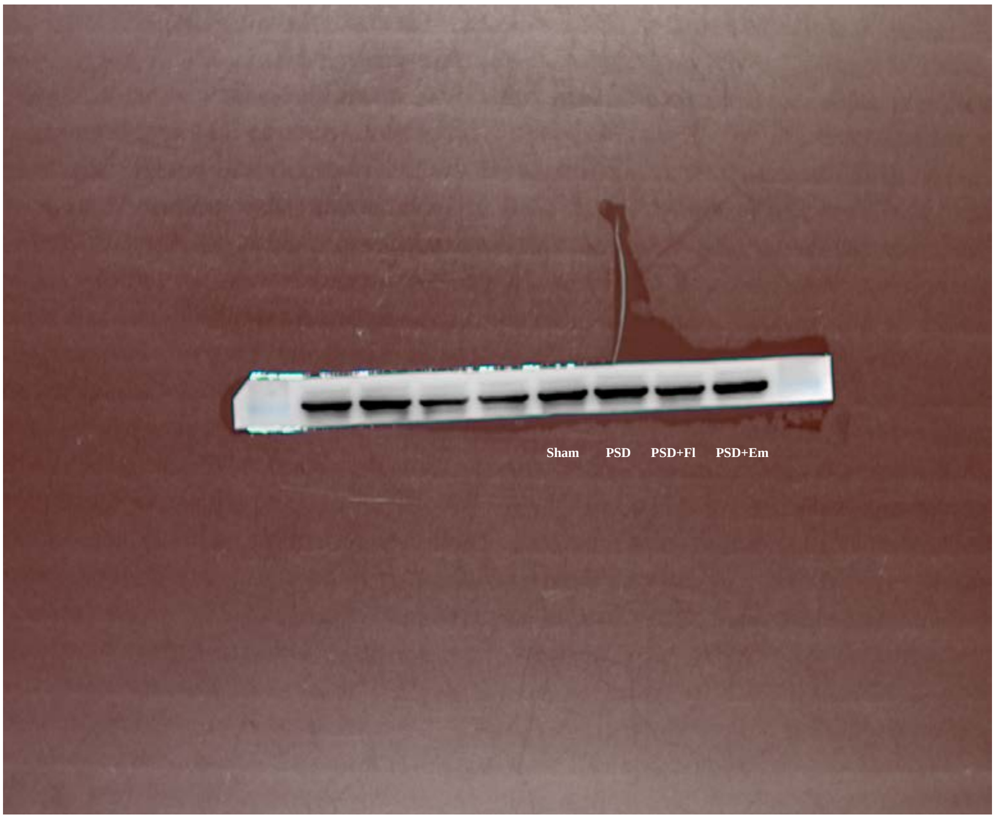

β-actin-1'

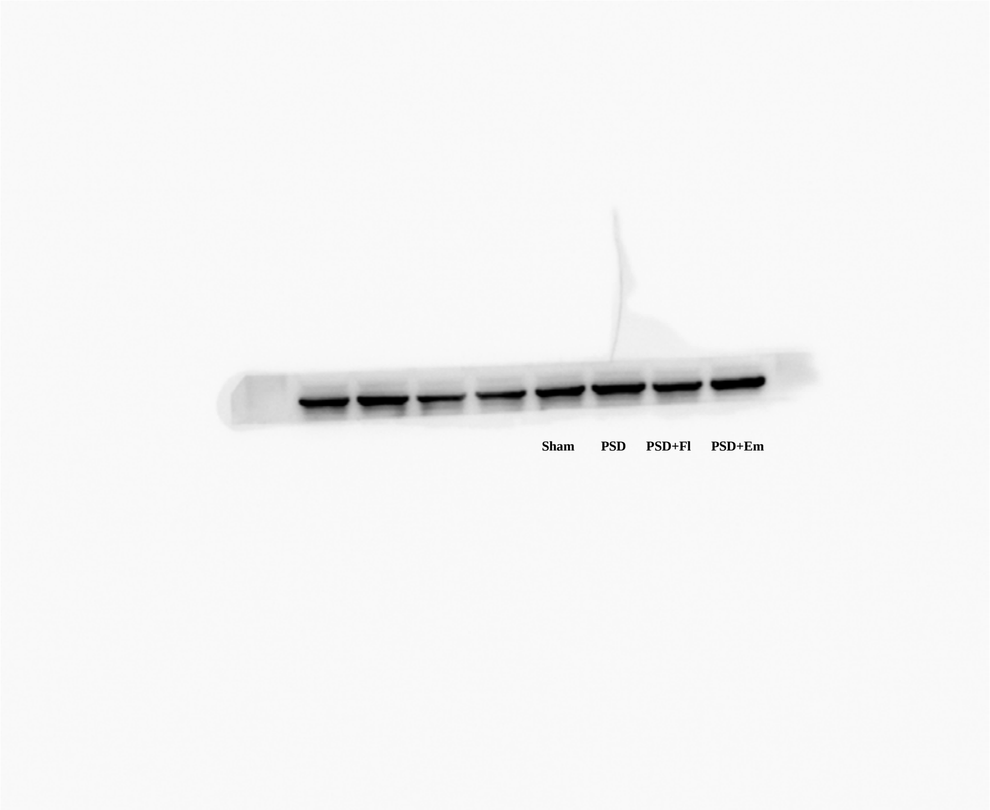

β-actin-2

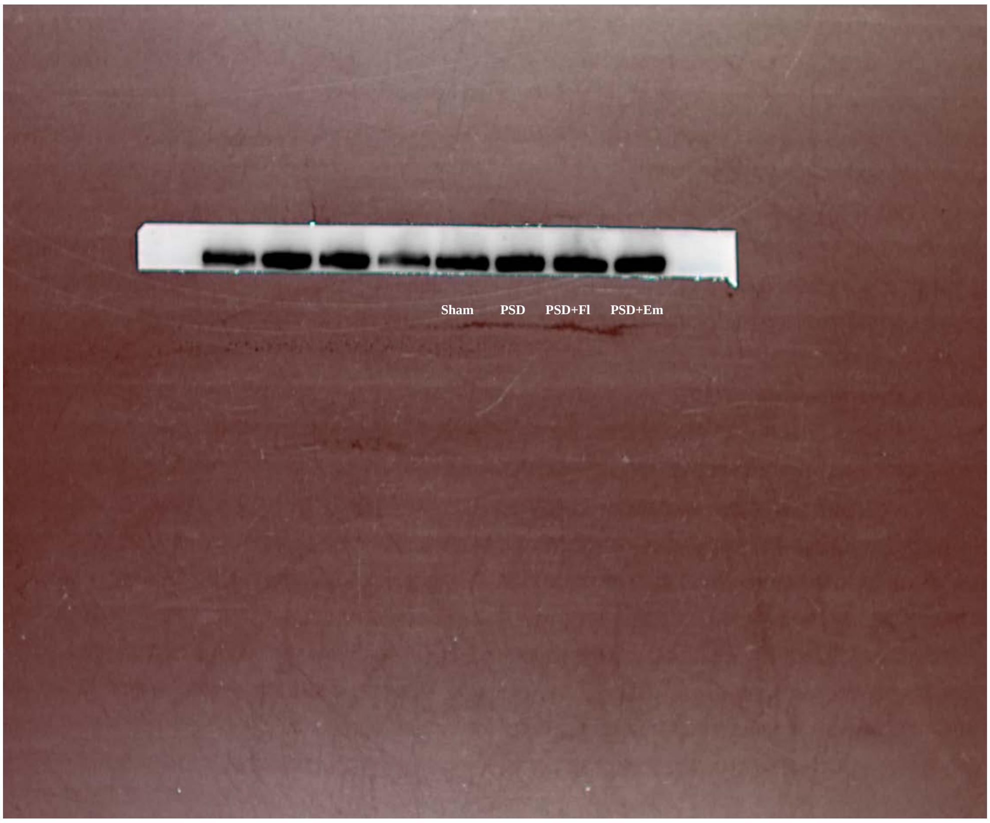

β-actin-2'

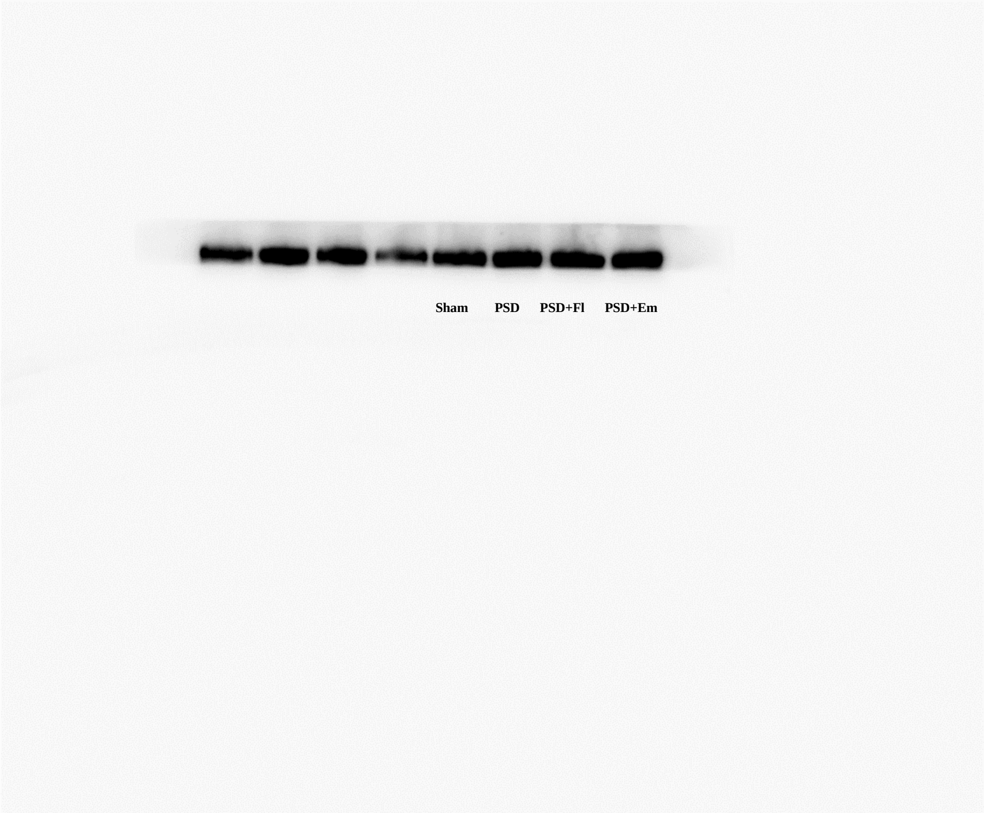

β-actin-3

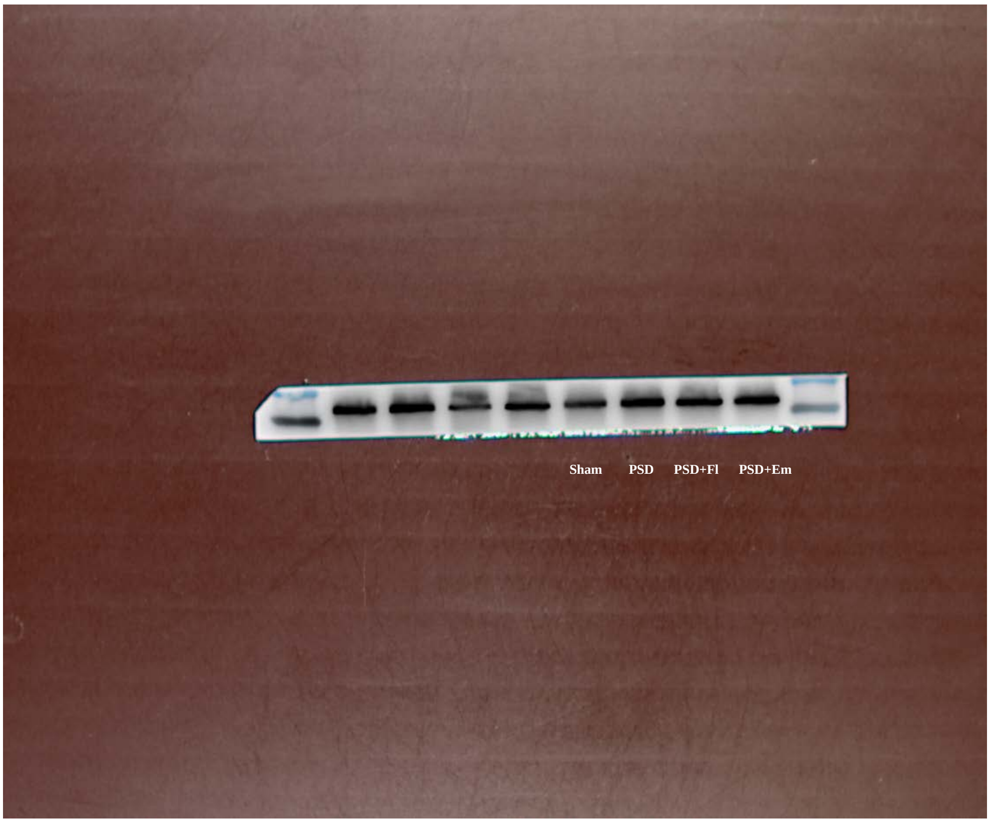

β-actin-3’

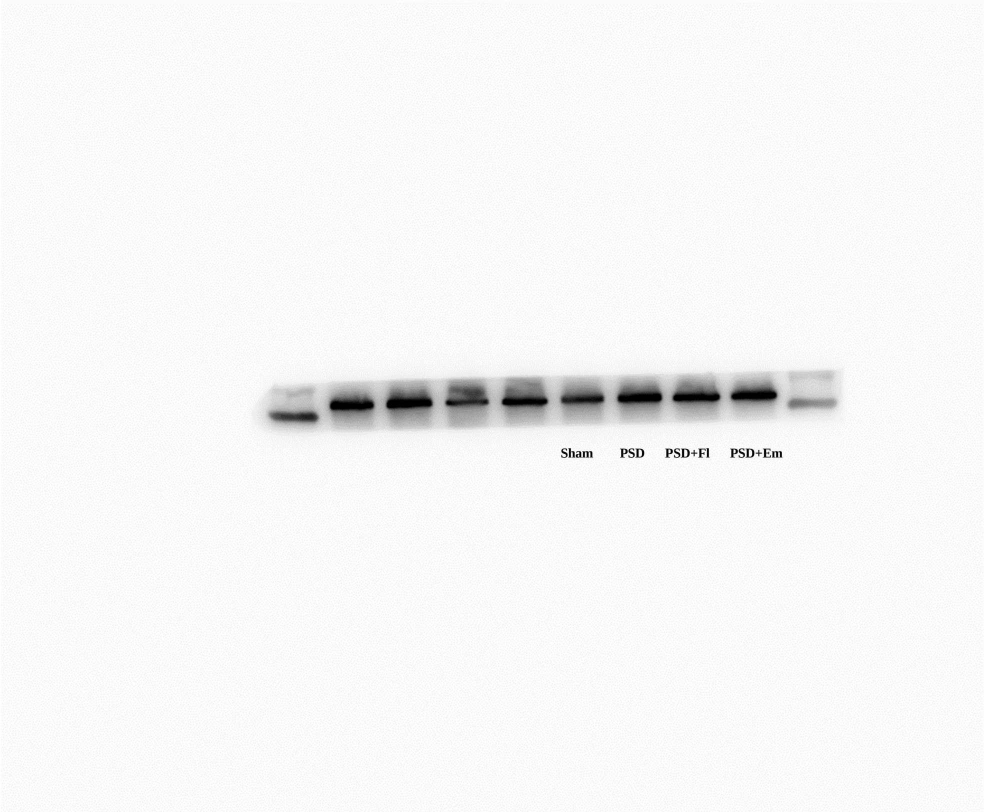

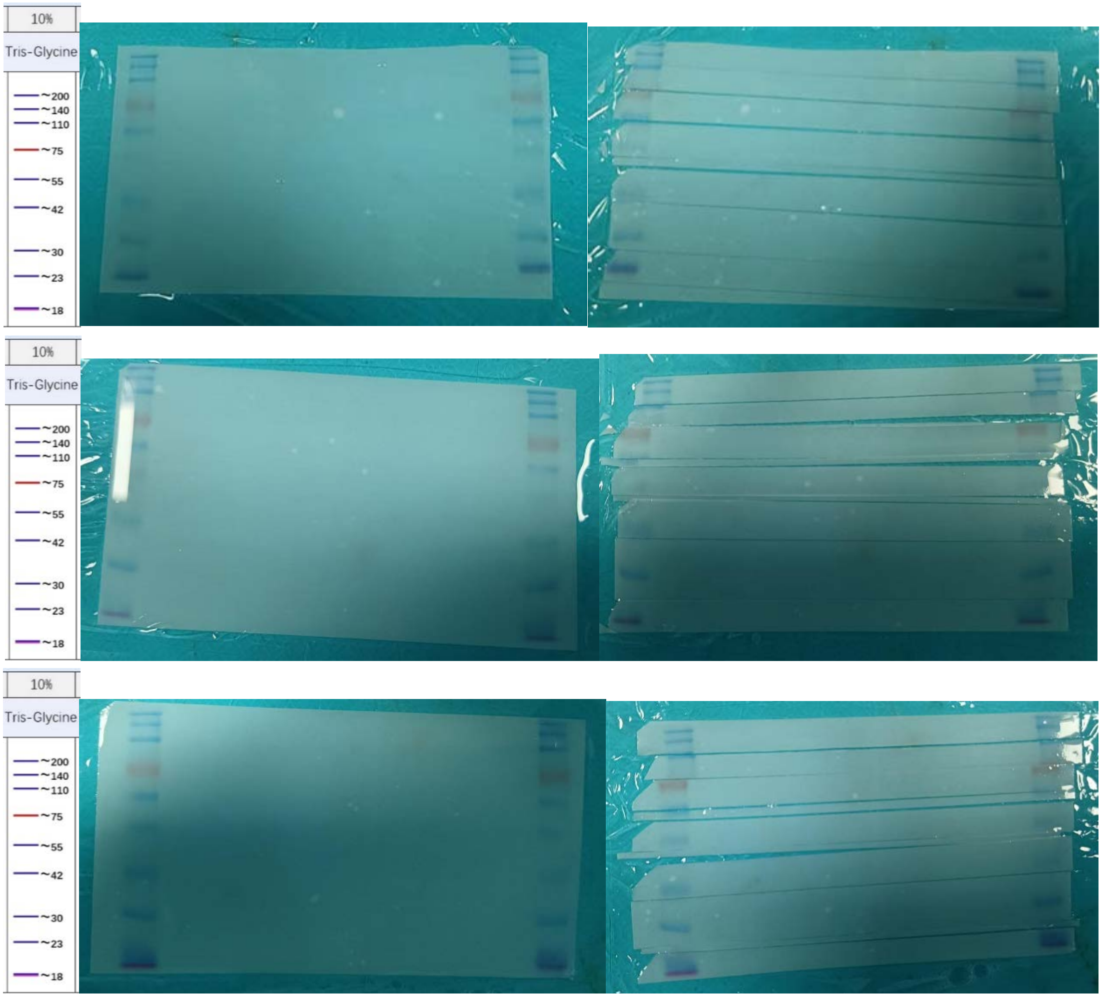

Figure 11E Complete unedited gel/imprint

MMP9-1

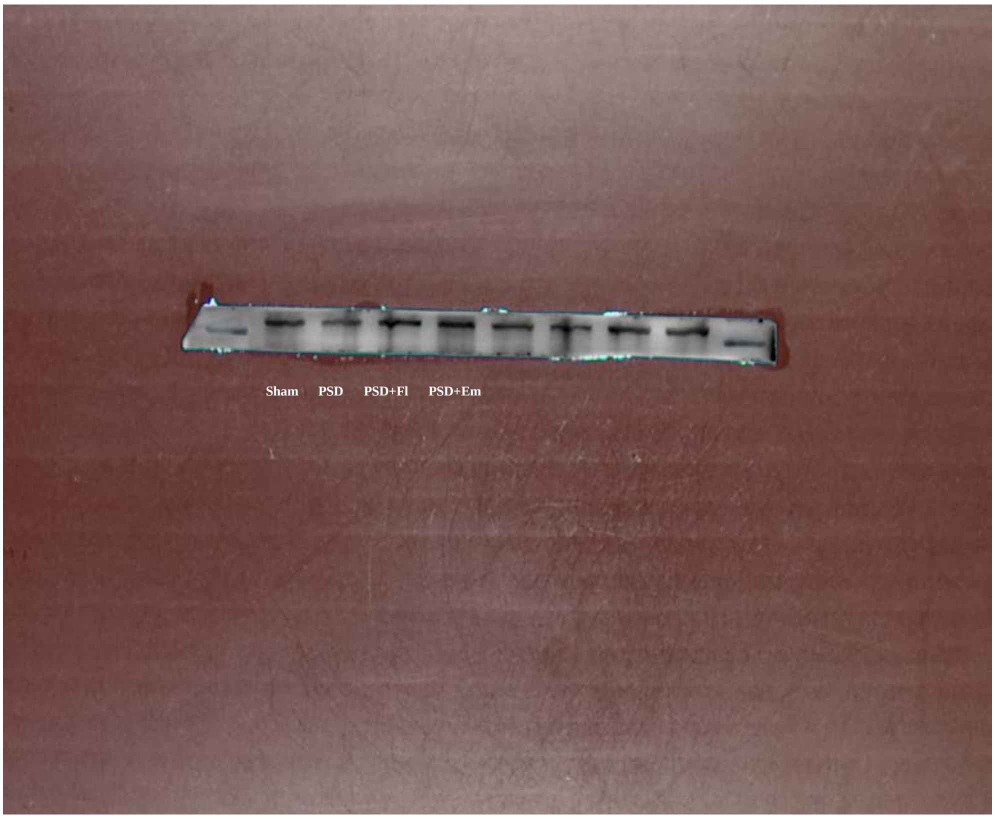

MMP9-1’

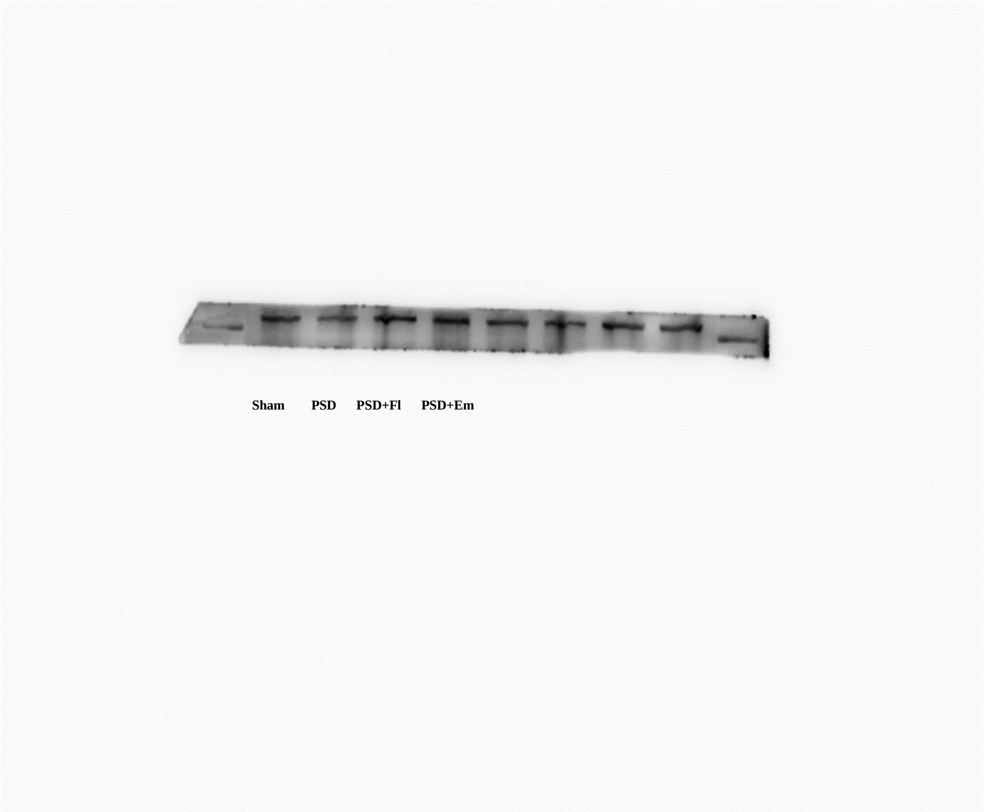

MMP9-2

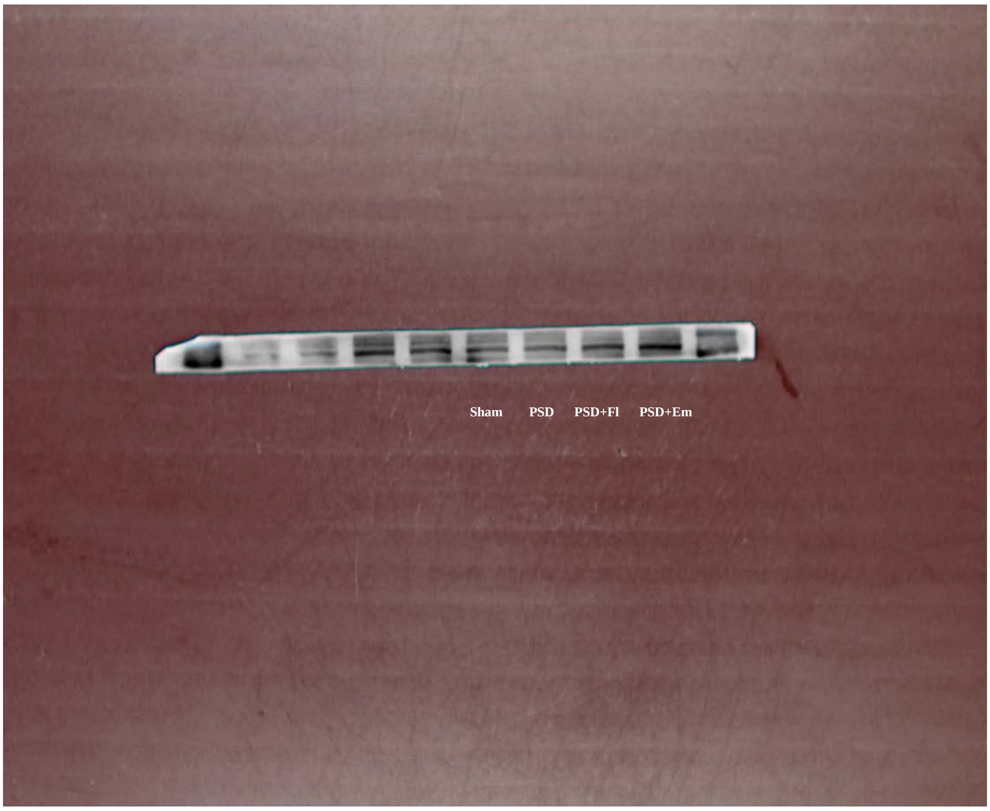

MMP9-2′

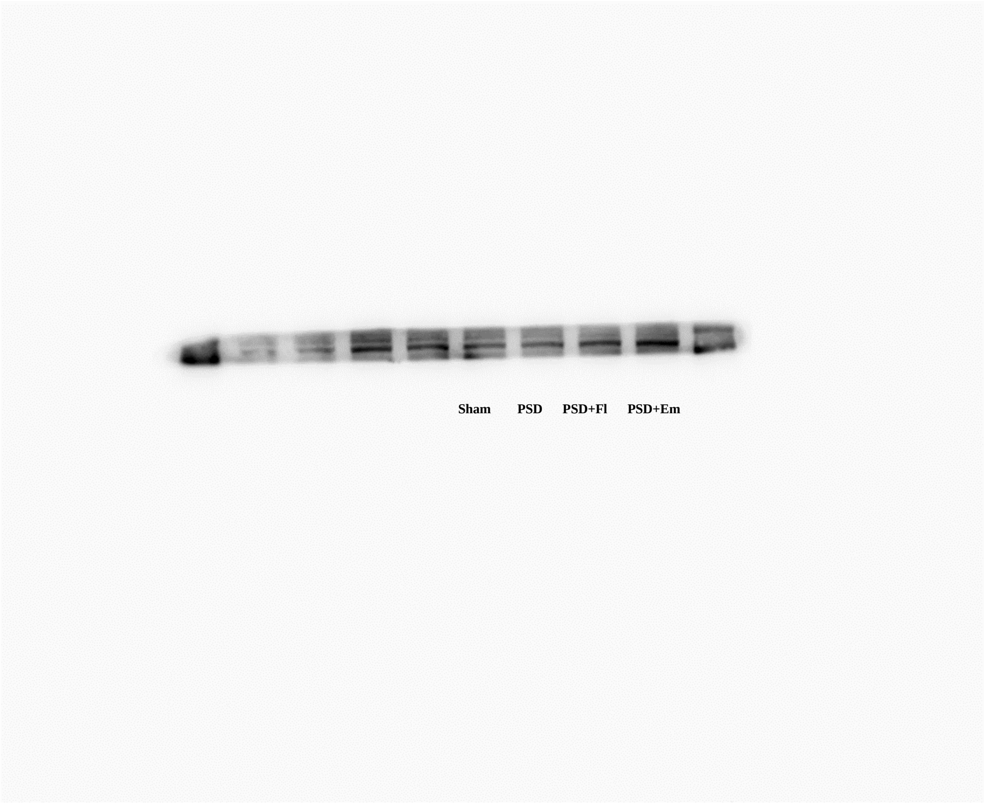

MMP9-3

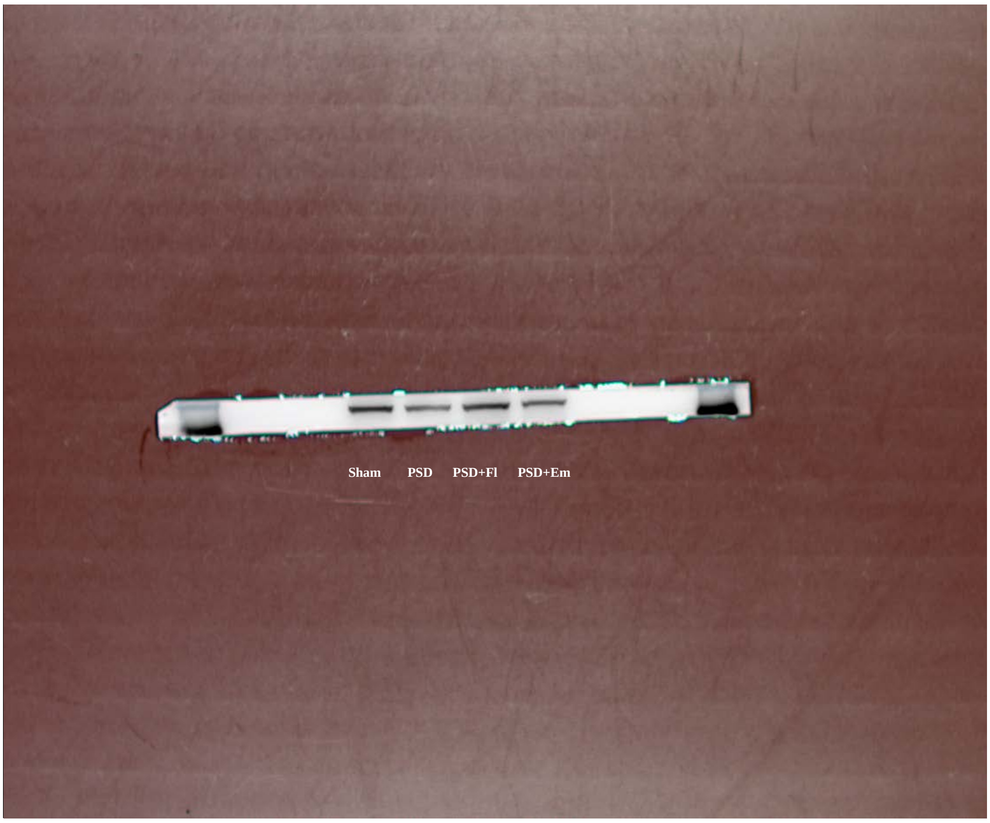

MMP9-3′

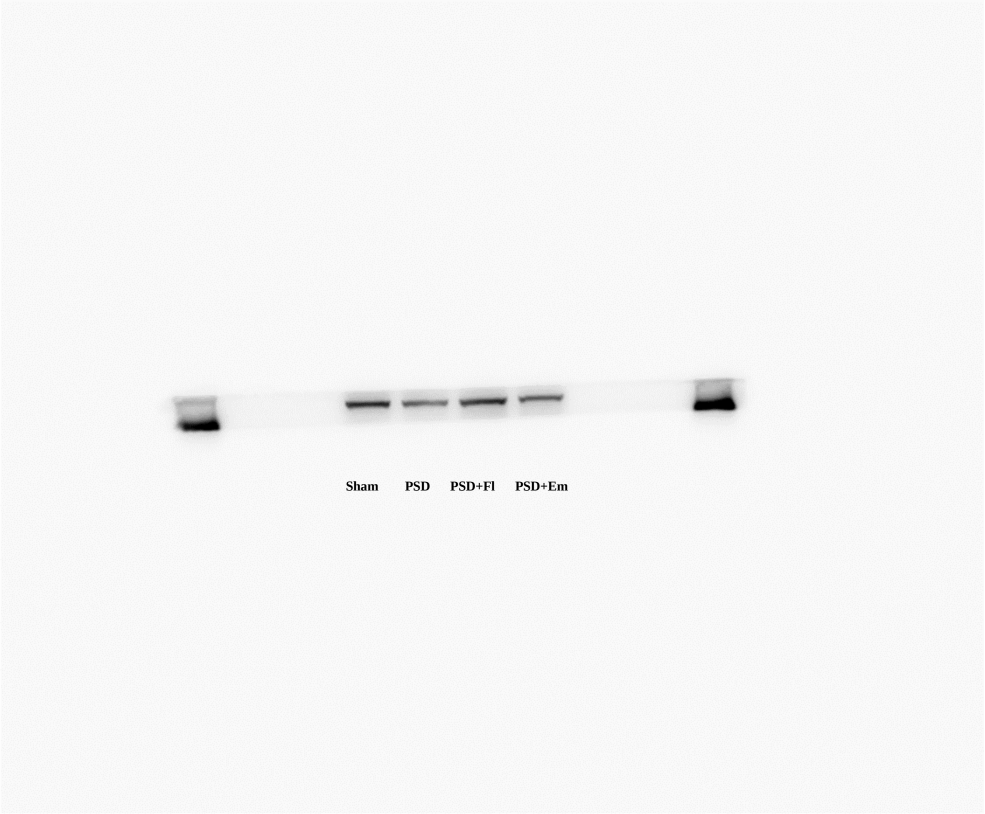

β-actin-1

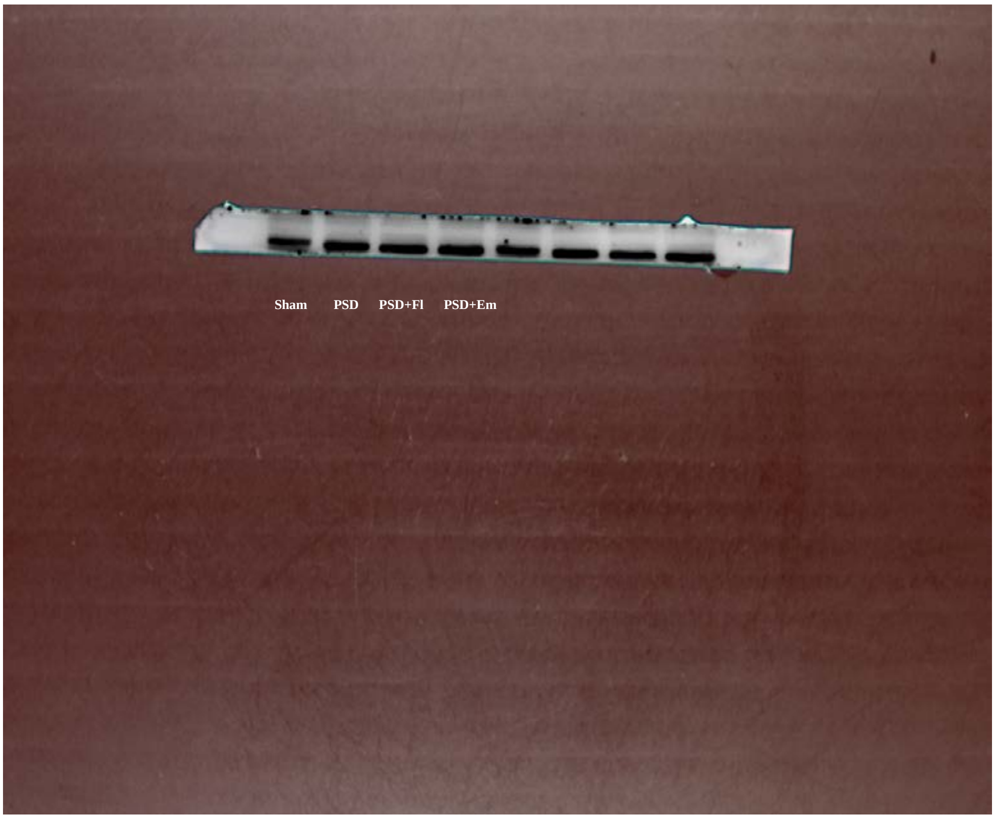

β-actin-1’

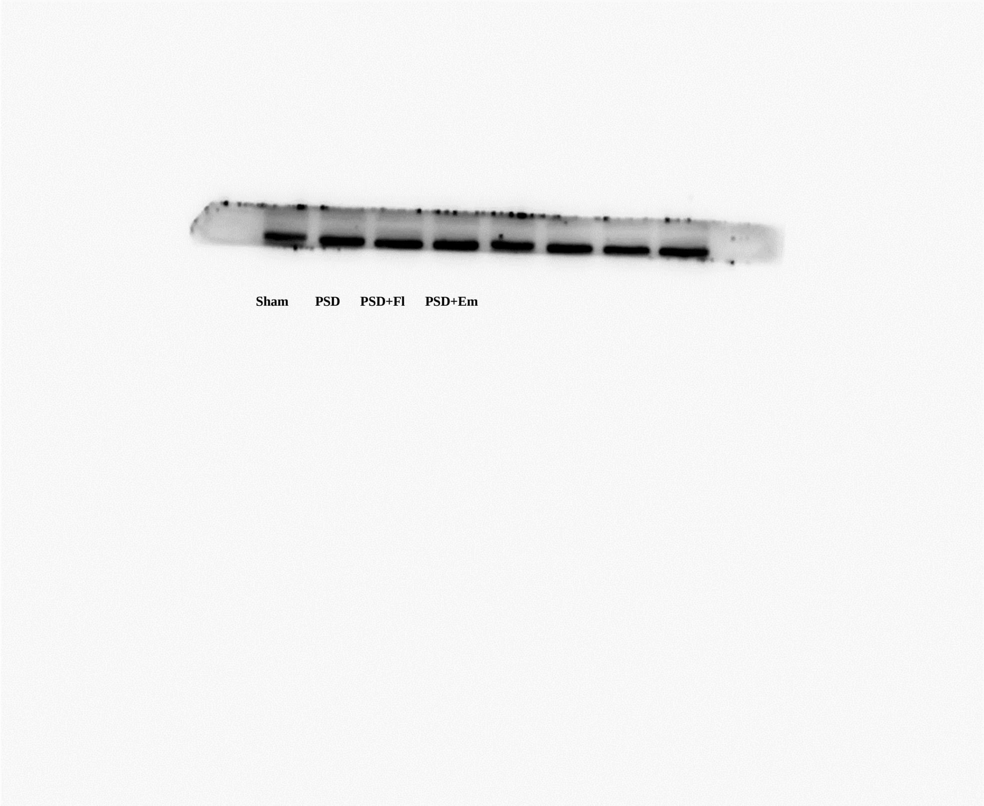

β-actin-2

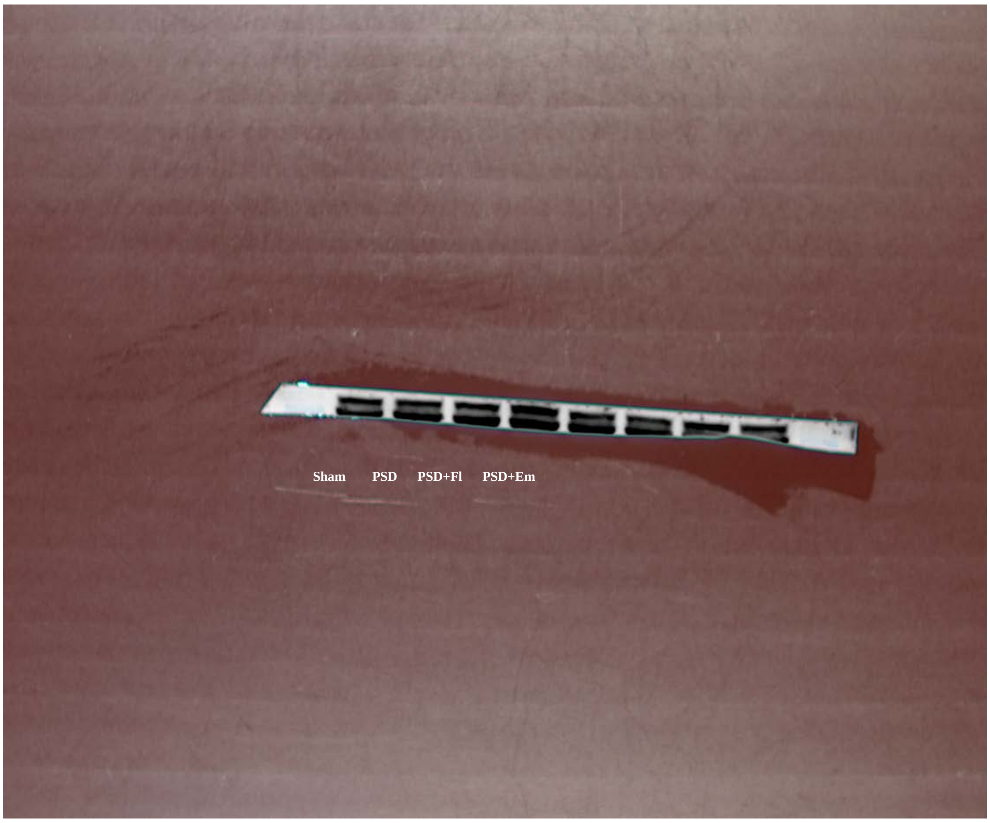

β-actin-2'

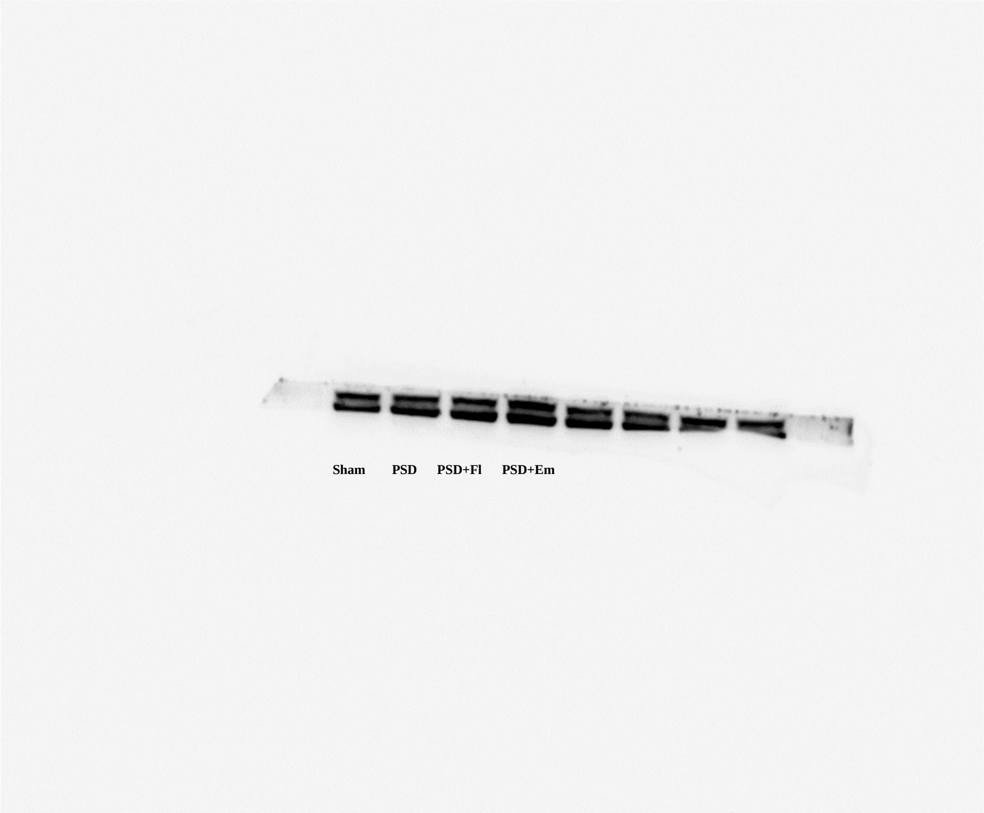

β-actin-3

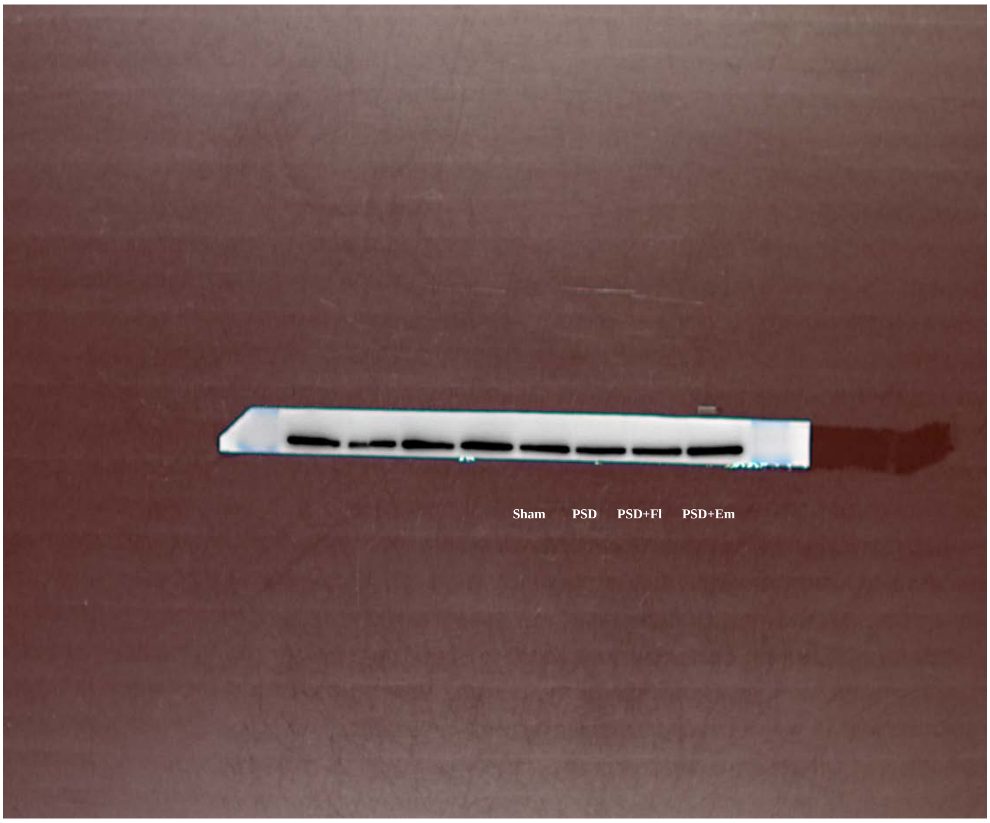

β-actin-3’

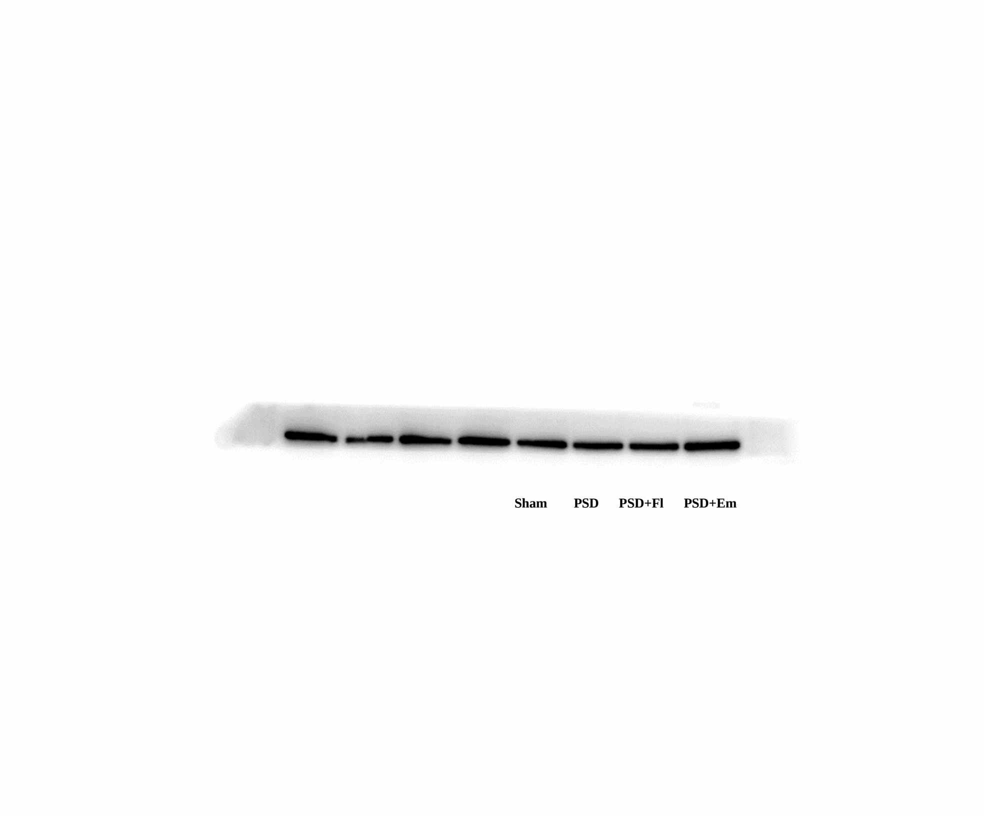

PC2-1

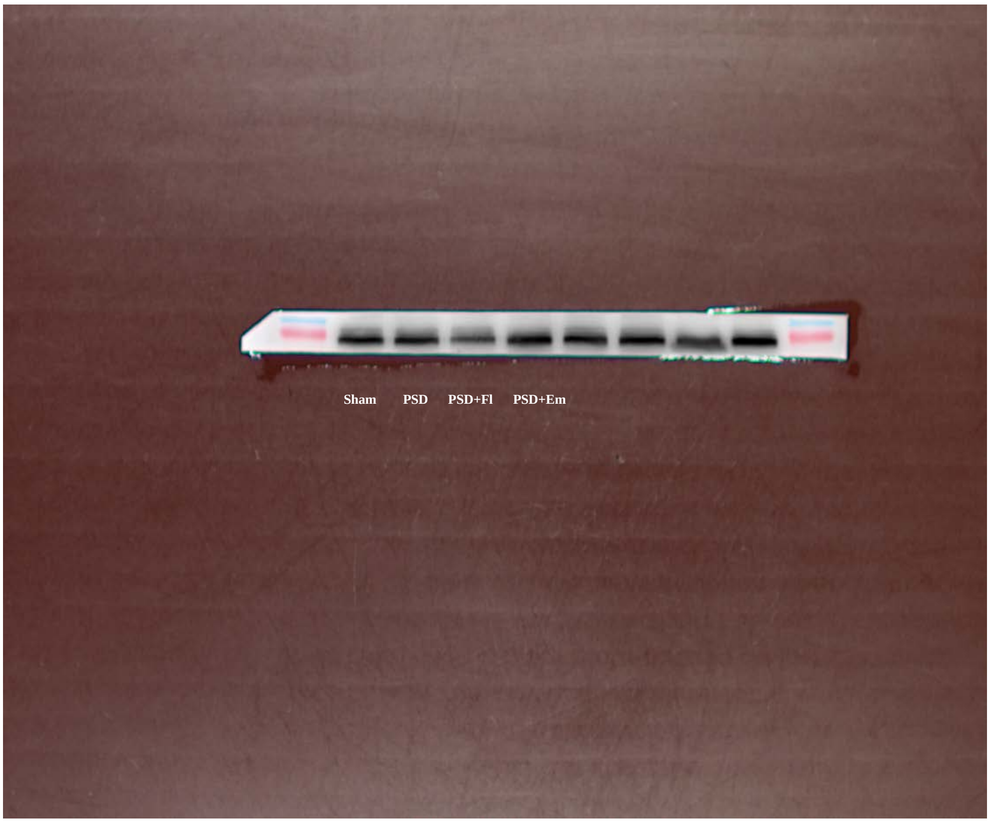

PC2-2

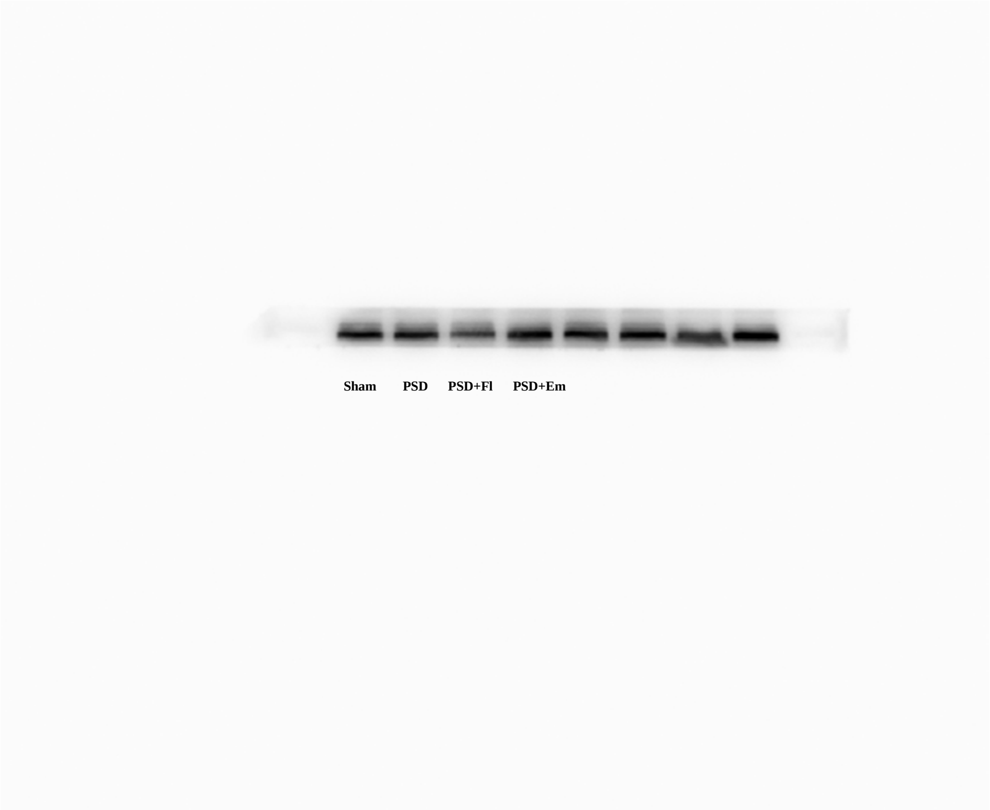

PC2-2

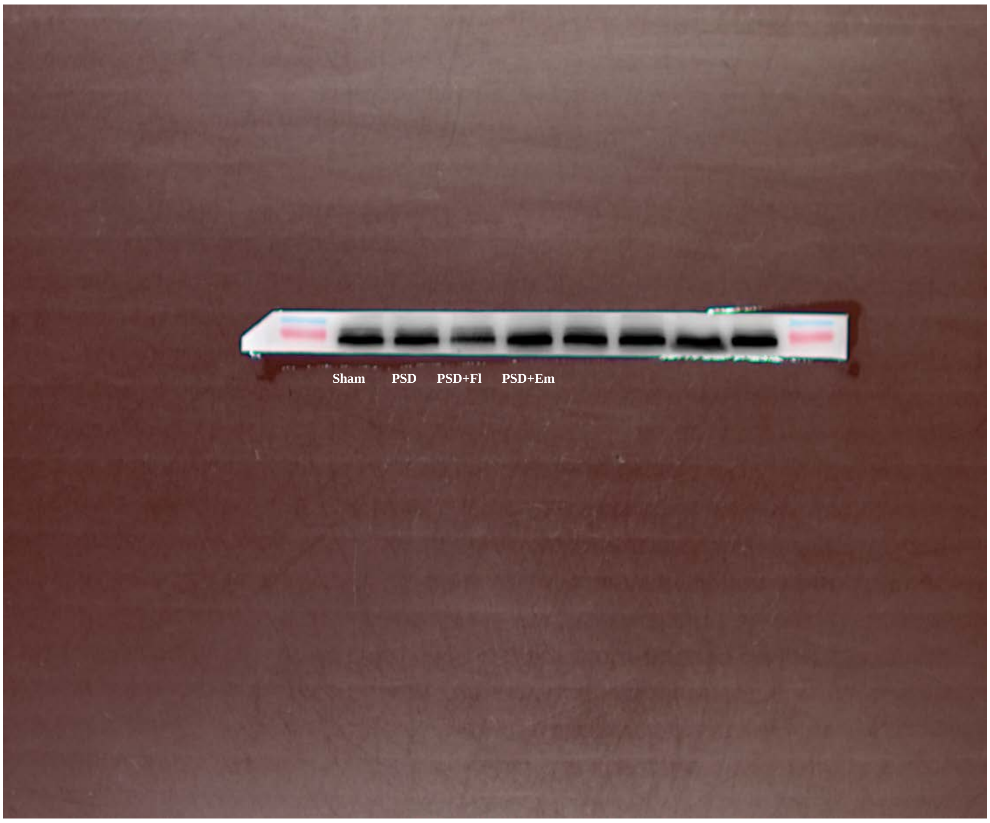

PC2-2’

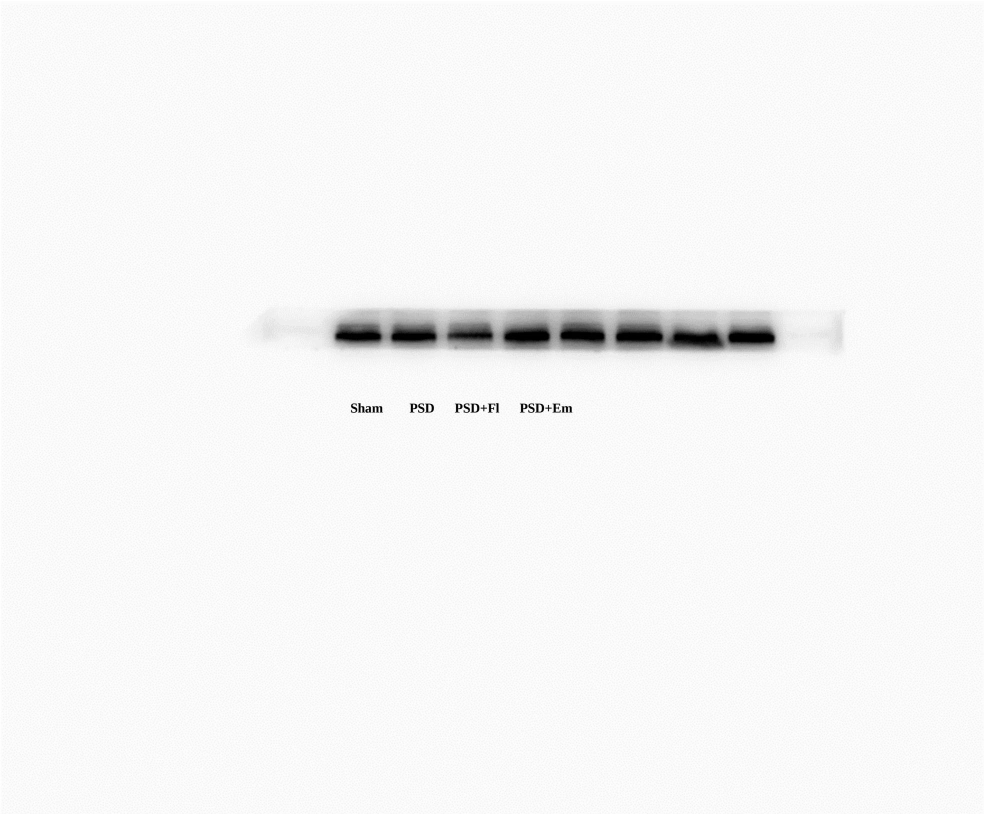

PC2-3

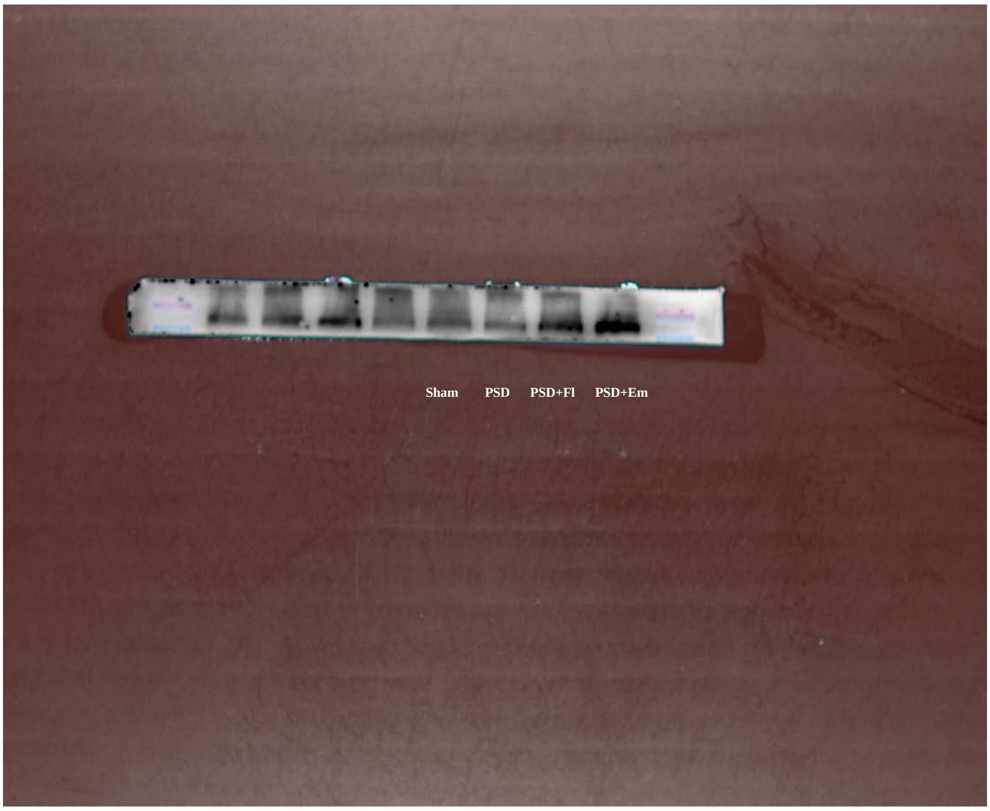

PC2-3’

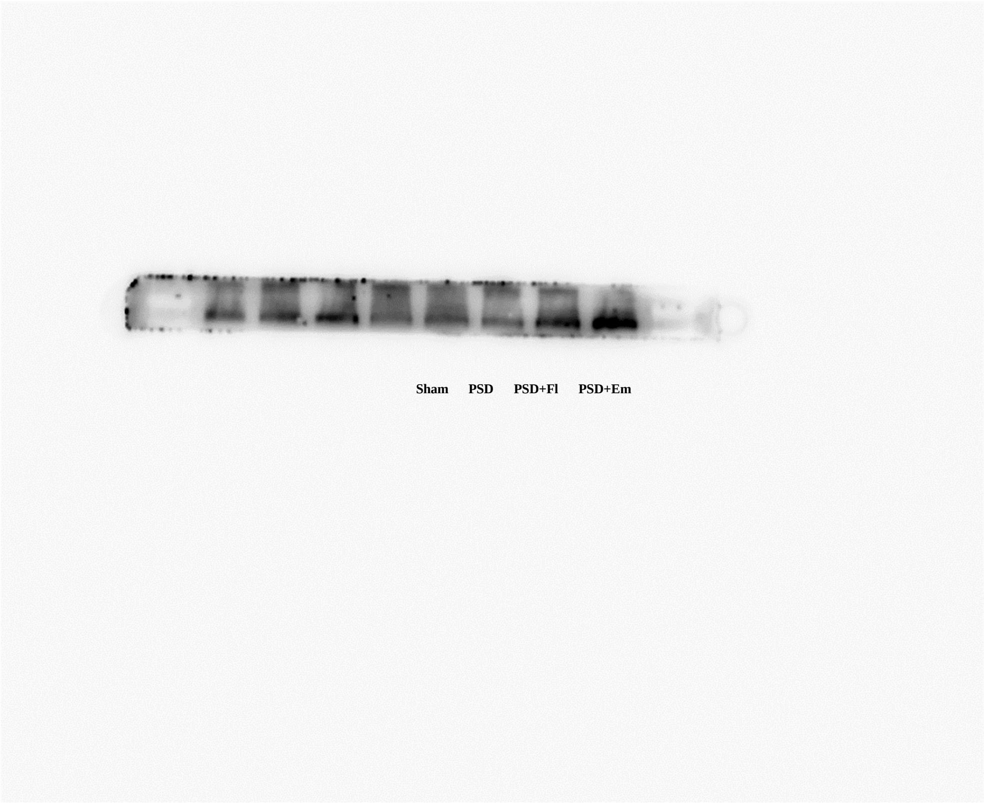

Supplement: Supplementary file 1 — Data S1: cns70581‐sup‐0001‐Supinfo.pdf. [file CNS-31-e70581-s001.pdf]
